# Supplementary material for: A glycine zipper motif is required for the translocation of a T6SS toxic effector into target cells
Source: EMBO Rep. 2023 Apr 17;24(6):e56849. doi: 10.15252/embr.202356849 (PMC10240207; doi:10.15252/embr.202356849)
Supplement: Supplementary file 1 — Appendix [file EMBR-24-e56849-s005.pdf]

Table of Content Appendix

Appendix Table S1. List of strains and plasmids --------------------------p1

Appendix Table S2. List of primers and plasmids -------------------------p4

Appendix References--p5

Appendix Table S1. List of strains and plasmids

Strain/plasmid Name	Relevant Characteristics	Source/Reference	EML No
A. tumefaciens			
C58	Wild type, virulent strain containing nopaline-type Ti plasmid pTiC58	Eugene Nester	EML530
ΔtssL	*tssL* deletion mutant	(Ma *et al*, 2009)	EML1073
Δtde1	Deletion of *atu4350*		
Δtdei	Deletion of *tde1-tdi1 (atu4350-atu4351)* and *tde2-tdi2 (atu3640-atu3639)* toxin immunity pairs	(Ma *et al*, 2014)	EML3559
ΔtdeiΔtssK	Deletion of *tssK* in *Δtdei*	This Study	EML5136
E. coli			
DH10B	F- *araDJ39 Δara, leu)7697 ΔlacX74 galU galK rpsL deoR φ80dlacZΔM15 endAI nupG recAI mcrA Δ (mrr hsdRMS mcrBC)*	Invitrogen	EML5396
BW25113	*rrnB Δ lacZ4787 hsdR514 Δ (araBAD)567 DE (rhaBAD)568 rph-1* (ME9062)	NBRP, Japan	EML5398
BW25113 (Δ*lacY*)	*lacY* deletion mutant of the BW25113	NBRP, Japan	EML5470
**Plasmids**			
pTrc200	Sm^R, Sp^R, pVS1 origin *lacIq*, *trc* promoter expression vector	(Schmidt-Eisenlohr *et al*, 1999)	EML5481
pRL662	Gm^R, non-conjugal transferable broad host range vector derived from pBBR1MCS-2,	(Vergunst *et al*, 2000)	EML5452
pRL662-*tdi1*-Strep	Gm^R, constitutive expression of Tdi1-Strep by *lac* promoter on the pRL662 plasmid	(Ma *et al.*, 2014)	EML3398
pTrc-*tde1(M)*-HA	Sp^R, C-terminal HA-tagged Tde1(M) driven by *trc* promoter on pTrc200	This Study	EML5445
pTrc-*N-tde1*-HA	Sp^R, N-terminus of Tde1 (1-49) with C-terminal HA tag driven by *trc* promoter on pTrc200	This Study	EML5482
pTrc-*N-tde1*^{GLGL}-HA	Sp^R, N-terminus of Tde1 (1-49) with amino acid substitutions (G39L, G43L) and HA tag on C-terminus driven by *trc* promoter on pTrc200	This Study	EML5464
pTrc-*tde1(M)*^{GLGL}-HA	Sp^R, C-terminal HA-tagged Tde1(M) with amino acid substitutions (G39L, G43L) driven by *trc* promoter on pTrc200	This study	EML5465
pTrc-*C1-tde1(M)*-HA	Sp^R, C-terminus of Tde1(M) (49-278) with C-terminal HA tag driven by *trc* promoter on pTrc200.	This study	EML5479
pTrc-*C2-tde1(WT)*-HA	Sp^R, C-terminus of Tde1 (98-278) with C-terminal HA tag driven by *trc* promoter on pTrc200.	This Study	EML5478
pYTA_*lacZ*	Amp^R, full length *lacZ* gene was cloned on to the YTA vector by TA cloning	This Study	EML1661

pBBRMCS2-*mCherry*	Km^R, full length *mCherry* gene was cloned on to pBBRMCS2 plasmid	This Study	EML3022
pTrc200-*lacY*	Sp^R, *lacY* from BW25113 with C-terminal HA tag driven by *trc* promoter on pTrc200 plasmid.	This Study	EML6350
pTrc-*N-tde1-sfGFP*	Sp^R, N-terminus of Tde1 (1-49) fused to the sfGFP on the C-terminal with a GGGS linker driven by *trc* promoter on pTrc200 plasmid	This Study	EML5489
pTrc-*N-tde1^GLGL^-sfGFP*	Sp^R, N-terminus of Tde1 (1-49) with point mutation (G39L, G43L) fused to the sfGFP on the C-terminal with a GGGS linker driven by *trc* promoter on pTrc200 plasmid	This Study	EML5490
pTrc-*tde1(M)-sfGFP*	Sp^R, full length of Tde1(M) fused to the sfGFP on the C-terminal with a GGGS linker driven by *trc* promoter on pTrc200 plasmid	This Study	EML5493
pTrc-*tde1(M)^GLGL^-sfGFP*	Sp^R, full length of Tde1(M) with point mutation on (G39L, G43L) fused to the sfGFP on the C-terminal with a GGGS linker driven by *trc* promoter on pTrc200 plasmid	This Study	EML5494
pTrc-*C1-tde1(M)*-HA	Sp^R, C-terminus of Tde1 (49-278) fused to the sfGFP on the C-terminal with a GGGS linker driven by *trc* promoter on pTrc200 plasmid	This Study	EML5492
pTrc-*C2-tde1(WT)*-HA	Sp^R, C-terminus of Tde1 (98-278) fused to the sfGFP on the C-terminal with a GGGS linker driven by *trc* promoter on pTrc200 plasmid	This Study	EML5491
pTrc200-*sfGFP*	Sp^R, sfGFP driven by *trc* promoter on pTrc200 plasmid	This Study	EML5495
pTrc-*tde1-tdi1*	Sp^R, expressopm of Tde1-Tdi1 driven by *trc* promoter on pTrc200 plasmid	(Wu et al., 2020)	EML4277
pTrc-*tde1^GLGL^-tdi1*	SpR, expression of Tde1-Tdi1 driven by trc promoter on pTrc200 plasmid	This Study	EML5513
pTrc-t*de1^G39L^-tdi1*	SpR, expression Tde1-Tdi1 with point mutation on (G39L) driven by trc promoter on pTrc200 plasmid	This Study	EML6358
pTrc-*tde1^G43L^-tdi1*	SpR, expression Tde1-Tdi1 with point mutation on (G43L) driven by trc promoter on pTrc200 plasmid	This Study	EML6359
pTrc-*tde1(M)^GLGL^-tdi1*	SpR, expression Tde1-Tdi1 with point mutation on (G43L) driven by trc promoter on pTrc200 plasmid	This Study	EML6360
pJN105	Gm^R, arabinose-inducible gene expression vector derived from pBBRMCS-1, araC-P_{BAD}	(Newman & Fuqua, 1999)	EML4220
pJN105-*tde1*	Gm^R, pJN105 expressing C-terminal 6xHis tagged Tde1	This study	EML6331
pJN105-*tde1^G39L^*	Gm^R, pJN105 expressing C-terminal 6xHis tagged Tde1 with the substitution of G39L	This study	EML3211

pJN105-*tde1*^{G43L}	Gm^R, pJN105 expressing C-terminal 6xHis tagged Tde1 with the substitution of G43L	This study	EML3212
pJN105-*tde1*^{GLGL}	Gm^R, pJN105 expressing C-terminal 6xHis tagged Tde1 with the substitutions of G39L and G43L	This study	EML6337
pJN105-*tde1(M)*	Gm^R, pJN105 expressing C-terminal 6xHis tagged Tde1 with the substitutions of H190A and D193A	This study	EML4289

Appendix Table S2. List of primers and plasmids

Name	Sequence (5' to 3')	Related construct(s)
NcoI_sfGFP_F<br>HindIII_ sfGFP _R	TGGACCCATGGACCGTAAAGGCGAAGAGCTGTTCA<br>TCGACAAGCTTTTTGTACAGTTCATCCA	pTrc200_*sfgfp*
NCoI_tde1_F	AAAAACCATGGACATGAGTGCGACGACAACTGTCG	pTrc200_*tde1(M)-HA*<br>pTrc200_*N-tde1-HA*<br>pTrc200_*N-tde1*^{GLGL}*-HA*<br>pTrc200_*tde1*^{G39L}*-Tdi1*<br>pTrc200_*N-tde1-sfgf*<br>pTrc200_*tde1(M)- sfgf*<br>pTrc200_*tde1(M)* ^{GLGL} *-sfgf*
XbaI-tde1_R	AAAAATCTAGACACCGGGACGTCAGGATTGCC	pTrc200_*tde1(M)-HA*<br>pTrc200_*C2-tde1(M)-HA*<br>pTrc200_*C1-tde1(M)-HA*<br>pTrc200_*tde1(M)* ^{GLGL} *-HA*
XbaI_N-tde1_R	AAAAATCTAGA A GCT CCC TCA GGC GGA CTA AAG CAA T	pTrc200_*N-tde1-HA*<br>pTrc200_*N-tde1*^{GLGL}*-HA*
NCoI_C2-tde_F	AAAAACCATGGACACGCCTAAGCAAGTTGAAGAGT TTAGG	pTrc200_*C2-tde1(M)-HA*
NCoI_C2-tde_R	AAAAACCATGGACGCGGGTAGAGCGGCTGCCTCC GCAA	pTrc200_*C2-tde1(M)-HA*
NcoI_C1_tde1_F	AAAAACCATGGACGCGGGTAGAGCGGCTGCCTCC GCAA	pTrc200_*C1-tde1(M)-HA*
G39L_G43L_F<br>G39L_G43L_R	TGGAGGTACAGCGGTTTTAGCATGGATACTTGGGC AAGTCGGCGCTAT<br>ATAGCGCCGACTTGCCCAAGTATCCATGCTAAAAC CGCTGTACCTCCAG	pTrc200_*N-tde1*^{GLGL}*-HA*<br>pTrc200_*tde1(M)*^{GLGL}*-HA*<br>pTrc200_*tde1(M)*^{GLGL}*-tdi1*
NcoI_lacY_F<br>XbaI_lacY_R	AAAAACCATGGACATGTACTATTTAAAAAACACAAA C<br>AAAAATCTAGAAGCGACTTCATTCACCTGACGAC	pTrc200_*lacY-HA*
*N-tde1*_GGGS _ sfGFP _F<br>sfGFP _GGGS_N-tde1_R	TTAGTCCGCCTGAGGGAGCTTCTAGAGGTGGTGGT TCTCGTAAAGGCGAAGAGCTGTT<br>ACAGCTCTTCGCCTTTACGAGAACCACCACCTCTA GAAGCTCCCTCAGGCGGACTAA	pTrc200_*N-tde1-sfGFP*
Hind III_sfGFP_R	AAA AAA AGC TTT TAT TTG TAC AGT TCA TCC ATA C	pTrc200_*N-tde1-sfgf*<br>pTrc200_*N-tde1*^{GLGL}*-sfgf*<br>pTrc200_*tde1(M)- sfgf*<br>pTrc200_*tde1(M)*^{GLG}*L-sfgfp*<br>pTrc200_*C1-tde1(M)-sfgfp*
pET-rbs-F1-XbaI<br>pET-R1-SacI	CCCCTCTAGAAATAATTTTGTTTAAC<br>GGAAGAGCTCCCTTTCGGGCTTTGTTAG	pJN105_*tde1*
Tde1_G39L_F<br>Tde1_G39L_R	ACAGCGGTTCTCGCATGGATAGGTGGGCAATC<br>GACTTGCCCACCTATCCATGCGAGAACCGCTGT	pTrc-*tde1 G*^{39L} *-tdi1*<br>pJN105_tde1^{G39L}
Tde1_G43L_F<br>Tde1_G43L_R	ACAGCGGTTGGAGCATGGATACTCGGG CAAGTC<br>GACTTGCCCGAGTATCCATGCTCCAACCGCTGT	pTrc-*tde1*^{G43L} *-tdi1*<br>pJN105_*tde1*^{G43L}

Appendix References

Ma LS, Hachani A, Lin JS, Filloux A, Lai EM (2014) Agrobacterium tumefaciens deploys a superfamily of type VI secretion DNase effectors as weapons for interbacterial competition in planta. *Cell Host Microbe* 16: 94-104

Ma LS, Lin JS, Lai EM (2009) An IcmF family protein, ImpLM, is an integral inner membrane protein interacting with ImpKL, and its walker a motif is required for type VI secretion system-mediated Hcp secretion in Agrobacterium tumefaciens. *J Bacteriol* 191: 4316-4329

Newman JR, Fuqua C (1999) Broad-host-range expression vectors that carry the L-arabinose-inducible Escherichia coli araBAD promoter and the araC regulator. *Gene* 227: 197-203

Schmidt-Eisenlohr H, Domke N, Baron C (1999) TraC of IncN plasmid pKM101 associates with membranes and extracellular high-molecular-weight structures in Escherichia coli. *Journal of Bacteriology* 181: 5563-5571

Vergunst AC, Schrammeijer B, den Dulk-Ras A, de Vlaam CM, Regensburg-Tuink TJ, Hooykaas PJ (2000) VirB/D4-dependent protein translocation from Agrobacterium into plant cells. *Science* 290: 979-982
