# Supplementary material for: A glycine zipper motif is required for the translocation of a T6SS toxic effector into target cells
Source: EMBO Rep. 2023 Apr 17;24(6):e56849. doi: 10.15252/embr.202356849 (PMC10240207; doi:10.15252/embr.202356849)
Supplement: Supplementary file 2 — Expanded View Figures PDF [file EMBR-24-e56849-s007.pdf]

Expanded View Figures

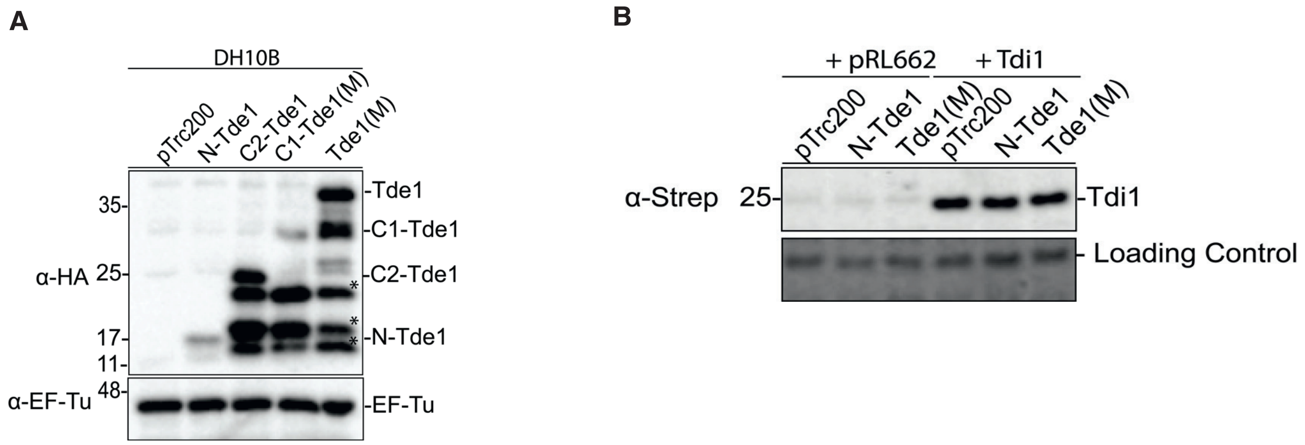

Figure EV1. Western blot analysis of Tde1 variants and Tdi1 in *Escherichia coli* growth inhibition assay.

A Western blot for the detection of the expression of HA-tagged Tde1 variants expressed in *E. coli*. *Other HA-tagged truncated bands, related to Fig 1B.

B Western blot for the detection of Tdi1 from *E. coli* cells co-expressing HA-tagged Tde1 variants and strep-tagged Tdi1, related to Fig 1C. The loading control is a nonspecific band from the western blot of anti-strep.

© 2023 The Authors

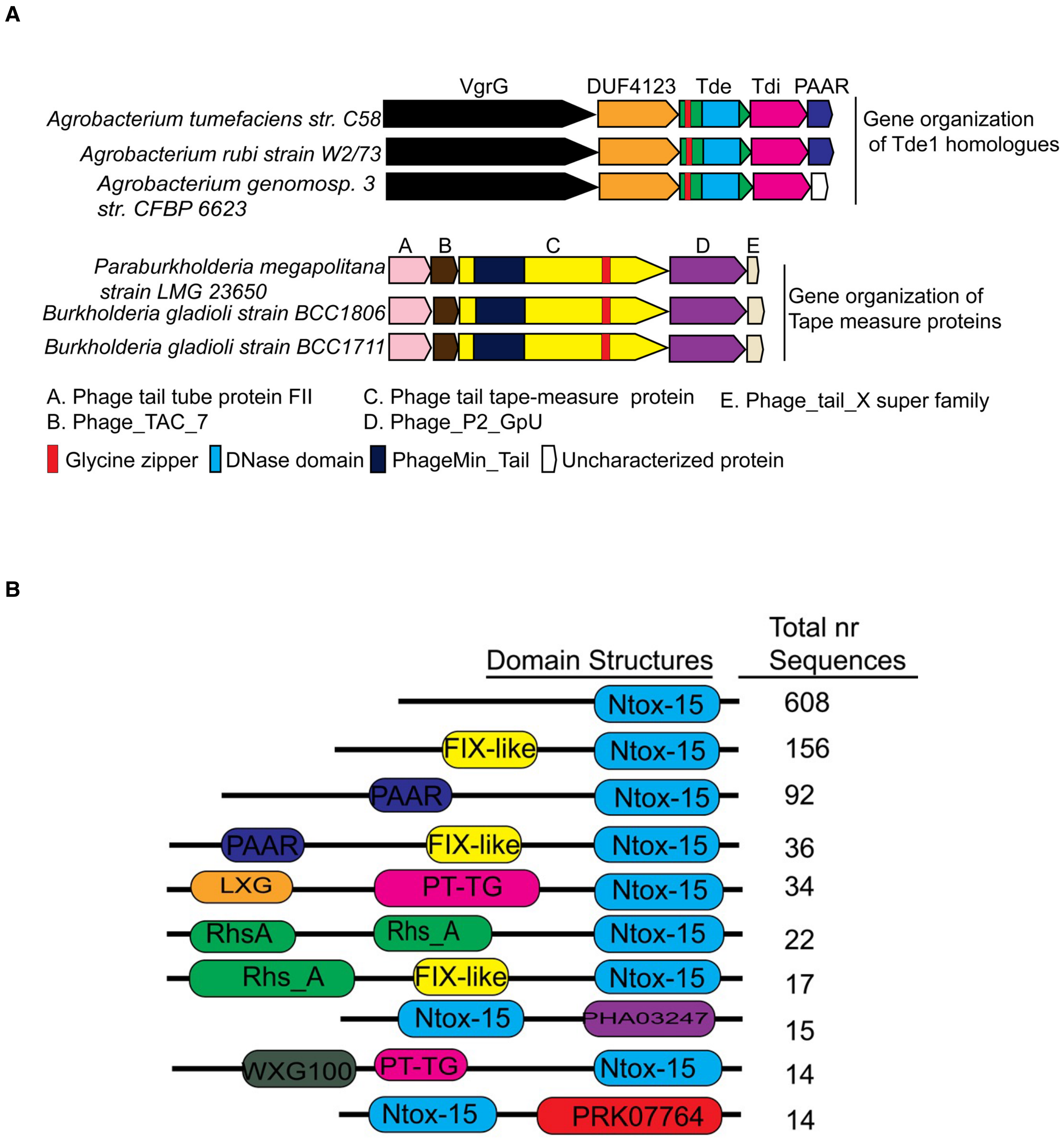

Figure EV2. Genetic organizations and domain architecture of Tde homologs.

A Genetic organizations of genes encoding representative Tde1 orthologues and Tape Measure Proteins (TMPs) with sequence similarity to the N-terminus of Tde1. The proteins encoded from the upstream and downstream of *tde1* and *tmp* genes are shown with their identified domain organizations.

B Domain architecture of the Ntox15-containing proteins. Top 10 classes of the Ntox15-containing proteins are shown with the identifiable domains (not to scale). The number of proteins in each class was indicated on the right based on the information on June 29, 2022. The *Agrobacterium tumefaciens* Tde1 belonged to the first class where the N-terminal region lacks an identifiable domain.

 © 2023 The Authors

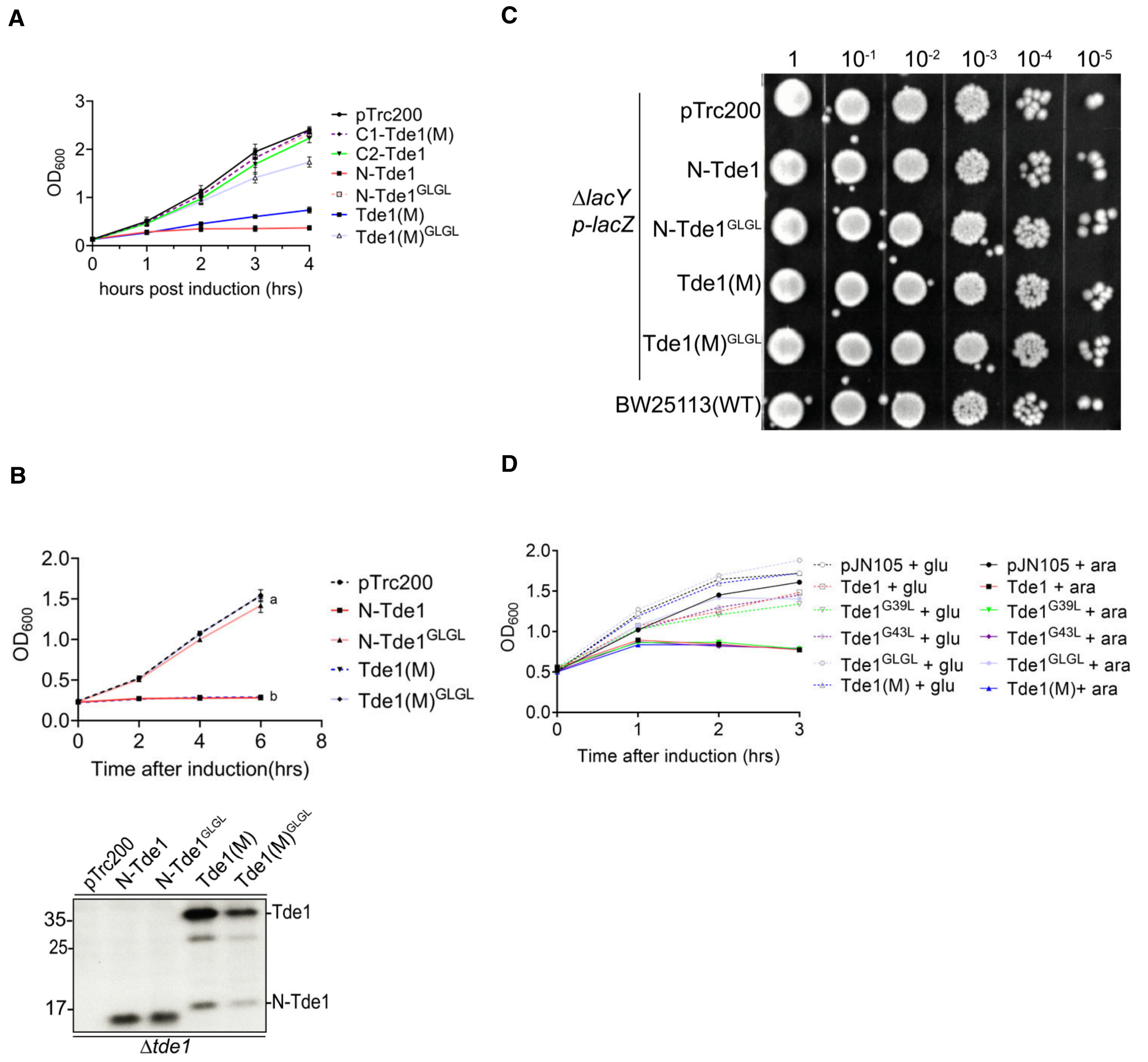

Figure EV3. Growth inhibition assays of Tde1 glycine zipper variants in *Agrobacterium tumefaciens* and *Escherichia coli*.

A Growth inhibition assay of *E. coli* DH10B cells harboring pTrc200 vector or each of its derivatives expressing Tde1 variants with IPTG-inducible expression, monitored by OD_{600}, related to Fig 2A. Graphs show mean ± SD of three biological replicates ($n = 3$), each averaged with 3 technical repeats.

B Growth curve and western blot analyses of *A. tumefaciens* C58 Δ*tde1* carrying pTrc200 or its derivatives expressing HA-tagged Tde1 variants. The growth curve was detected every 2 h in 523 media supplemented with 1 mM IPTG. Graphs show mean ± SD of three biological replicates ($n = 3$), each averaged with 3 technical repeats. One-way ANOVA was used for the analysis of statistical significance followed by the Tukey's multiple comparison. Different letters indicate statistically different groups of strains (P value $= 6.47 \times 10^{-11}$). The proteins collected at the end point (6 h) were analyzed for western blotting with antibodies against HA. Representative results of three biological repeats were shown. Protein markers are indicated in kDa.

C Viability assay for *E. coli* cells derived from the ONPG uptake assay after 1 h IPTG induction, related to Fig 2B.

D The growth curve analysis of *E. coli* cells used for *in vivo* plasmid DNA degradation assay. The turbidity of *E. coli* BW25113 expressing Tde1 and its variants carried out for the *in vivo* plasmid DNA degradation assay was measured. The *E. coli* cells were supplemented with 0.5% glucose (glu) or 0.2% L-arabinose (ara) for the repression or induction of Tde1 and its variants. The OD_{600} values were measured by DEN-600 photometer (Biosan, Latvia) every hr.

© 2023 The Authors

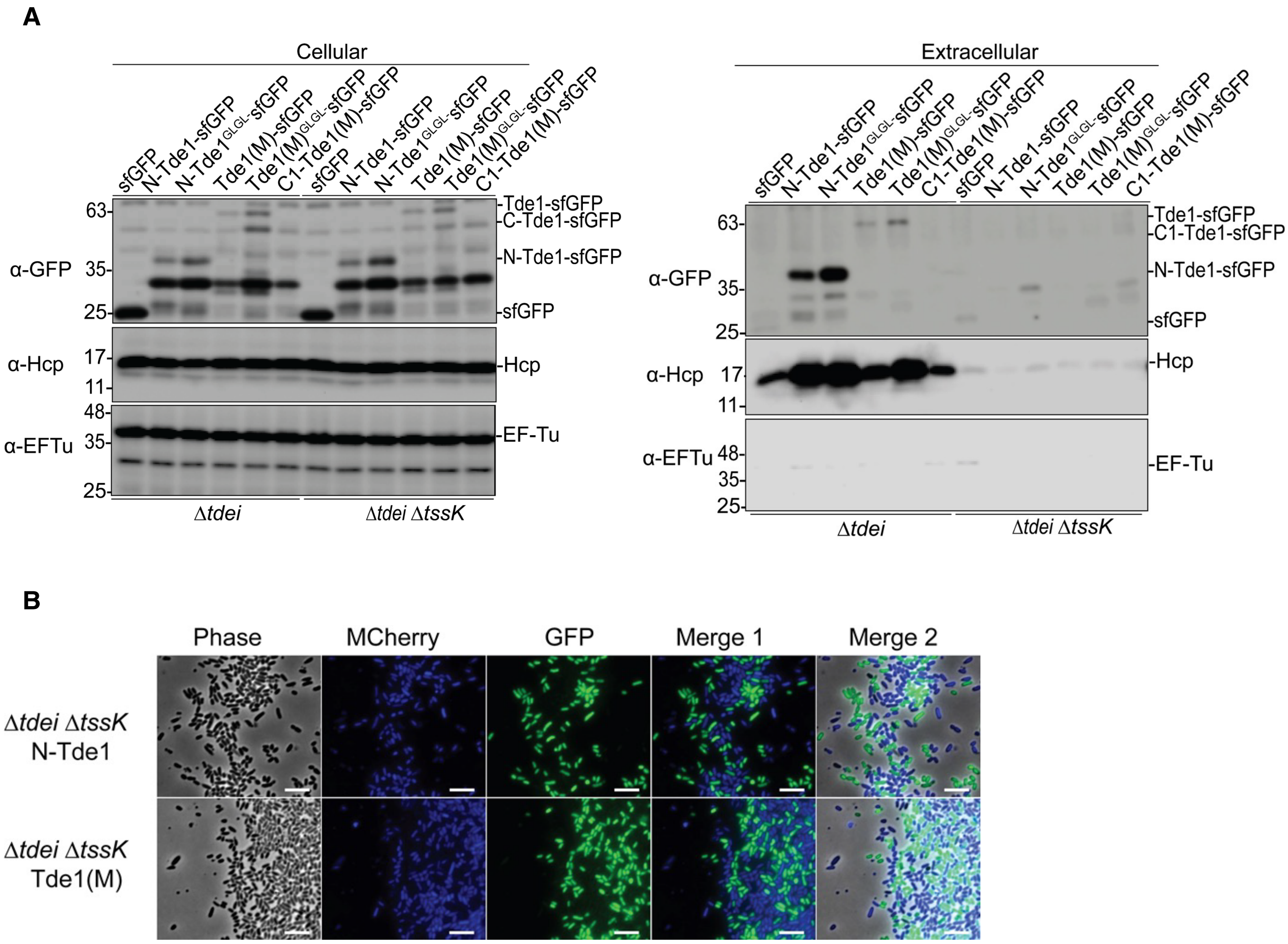

Figure EV4. Secretion assay for sfGFP-fused Tde1 variants and fluorescence microscopy for negative controls of translocation assay.

A Secretion assay for Tde1 variants fused with sfGFP. Western blot for the cellular and extracellular fractions of *Agrobacterium tumefaciens* C58 Δ*tdei* and Δ*tdei*Δ*tssK* expressing the Tde1 variants fused with sfGFP were detected by anti-GFP antibody. Representative western blot results of three biological repeats were shown with antibody against GFP, Hcp, or EF-Tu where EF-Tu served as a loading and nonsecreted protein control. Hcp secretion served as a positive control for active T6SS secretion. Protein markers are indicated in kDa.

B *A. tumefaciens* C58 Δ*tdei*Δ*tssK* expressing N-Tde1-sfGFP or Tde1(M)-sfGFP (in green) and *E. coli* DH10B carrying mCherry (false colored in blue) were co-cultured for 20 h. No cyan fluorescence with merged blue and green signals could be detected when attacker cells are T6SS-inactive, which served as negative controls for the translocation assay (Scale bar = 5 μm).

 © 2023 The Authors

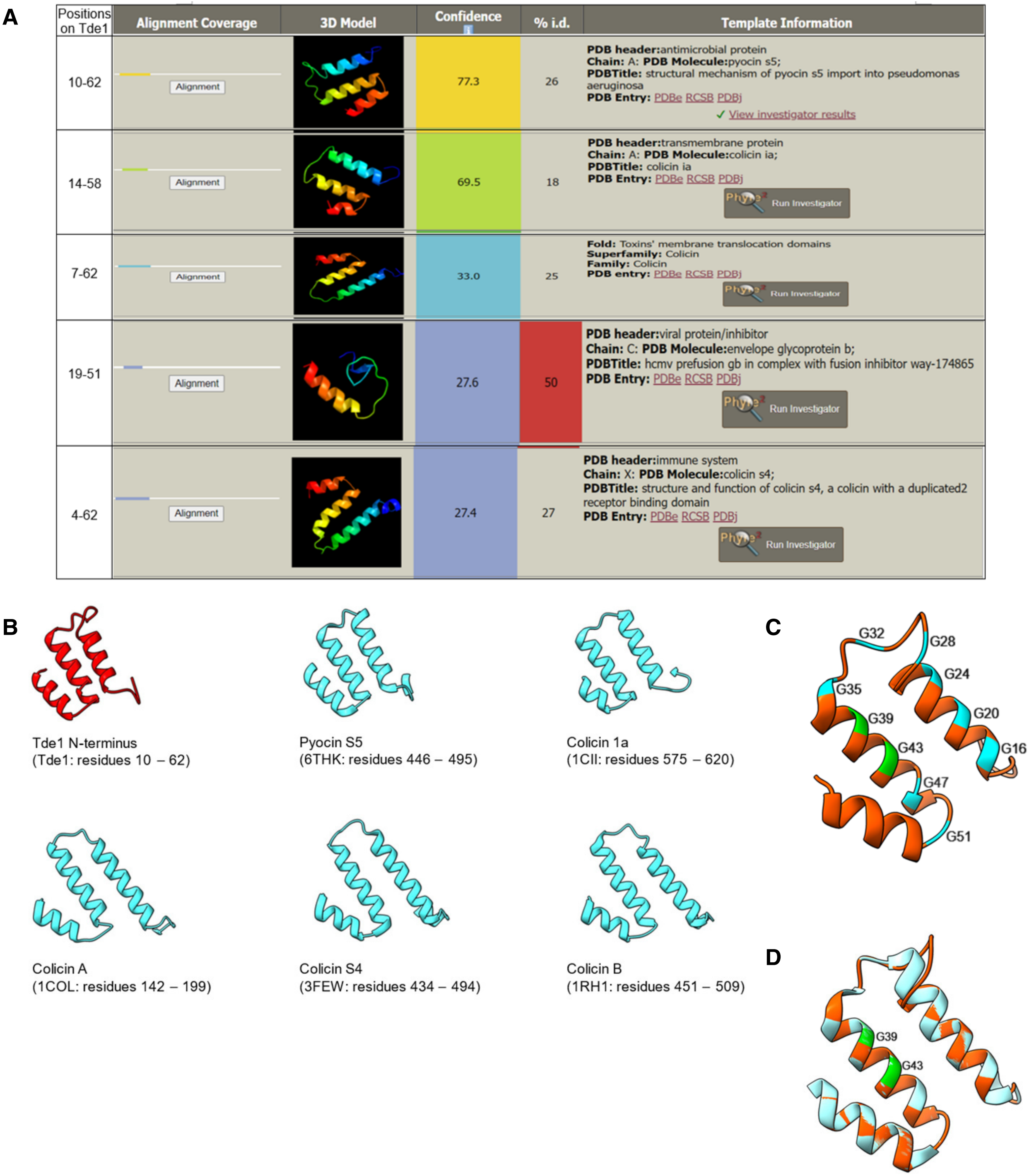

Figure EV5. Structural prediction of the Tde1 N-terminus with similarity to pyocin S5 and colicins.

- A Predicted results of N-terminal Tde1 (1–97) as a query reveal structural similarity to pyocin S5 and colicin Ia with high confidence.
- B N-terminal Tde1 with structural similarity to pore-forming domain of the pyocin S5, colicines, and other membrane perturbing proteins based on Phyre2 prediction.
- C Cartoon model of the Tde1 (residue 10–62) by using on the basis of the crystal structure of Pyocin S5 (PDB 6THK) with 77.3% of confidence level. All glycine residues of the predicted glycine zipper motif of Tde1 were indicated.
- D Superimposition of N-Tde1 and pore-forming domain of pyocin S5. Tde1 N-terminus is in red, and the partially pore-forming domain of pyocin S5 is in teal; G^{39} and G^{43} in the putative glycine zipper motif are highlighted in green.

Data information: All data were analyzed by Phyre2 server.
