# Supplementary figures and images for: A glycine zipper motif is required for the translocation of a T6SS toxic effector into target cells
Source: EMBO Rep. 2023 Apr 17;24(6):e56849. doi: 10.15252/embr.202356849 (PMC10240207; doi:10.15252/embr.202356849)

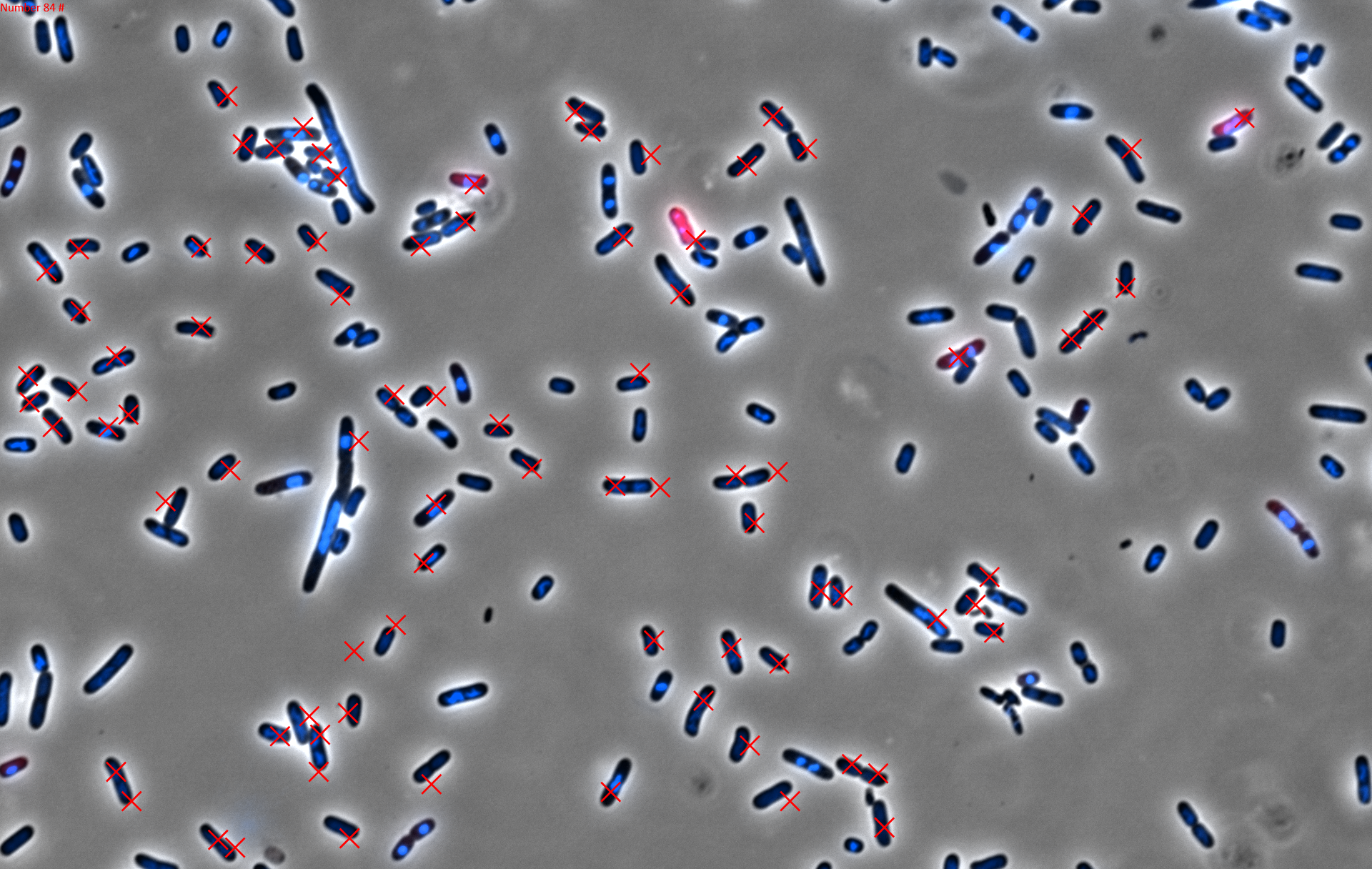

Supplement: Supplementary file 5 — Source Data for Figure 2 [file EMBR-24-e56849-s009.zip › 2C. Image and numerical data Micr.image+quantif/2C. Micr.image/pTrc200/pTrc200_1.tif]

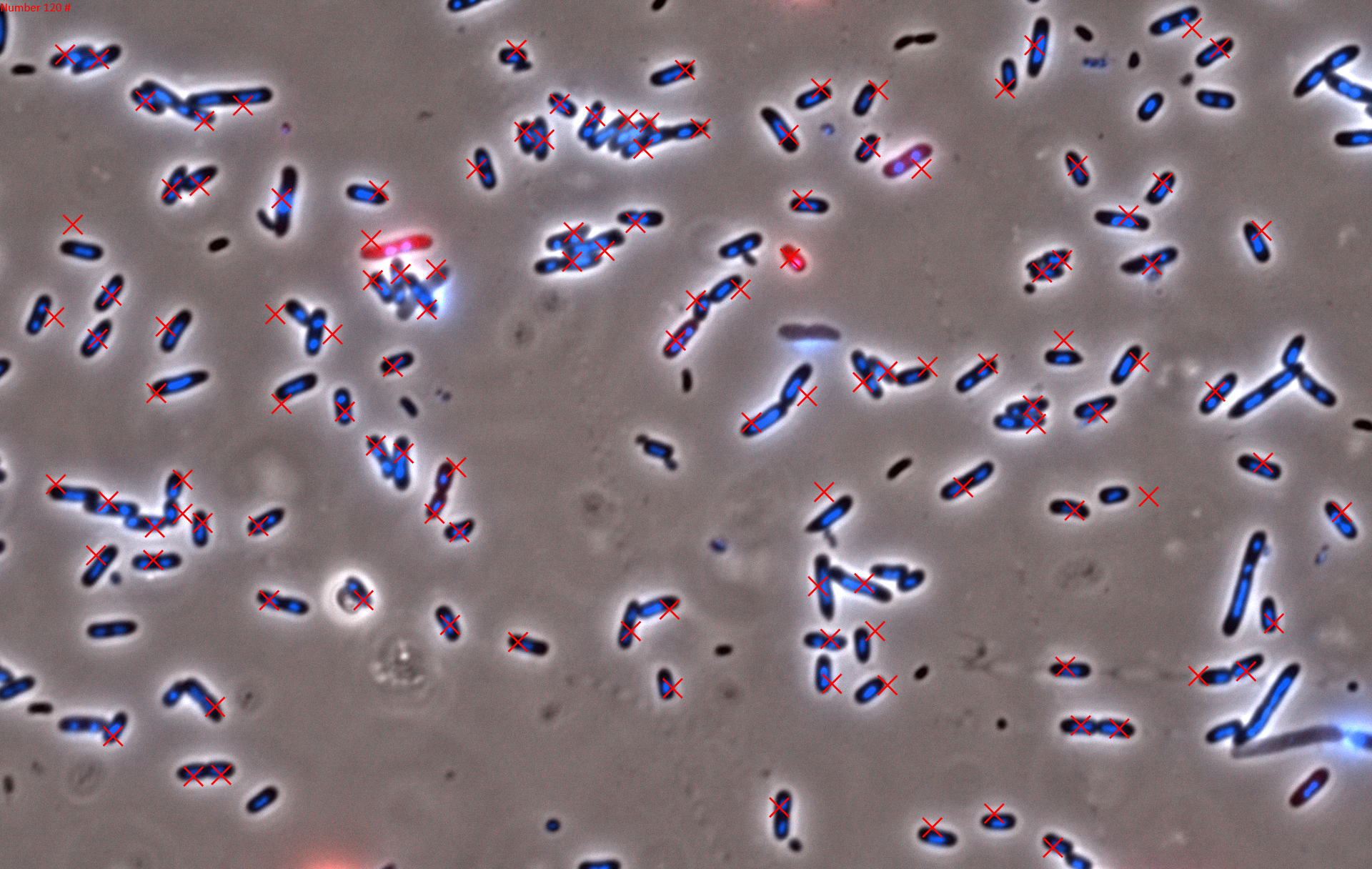

Supplement: Supplementary file 5 — Source Data for Figure 2 [file EMBR-24-e56849-s009.zip › 2C. Image and numerical data Micr.image+quantif/2C. Micr.image/pTrc200/pTrc200_2.tif]

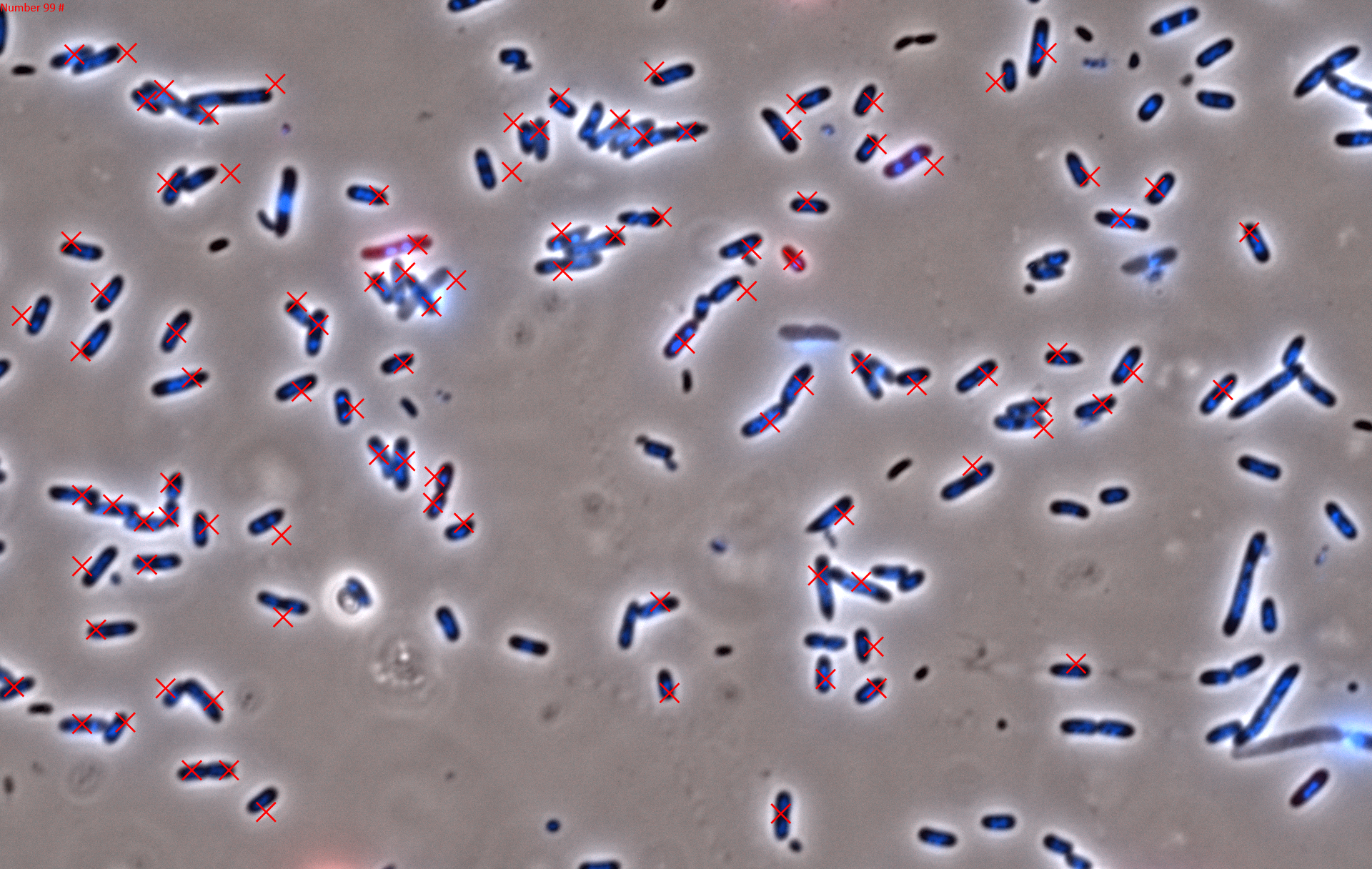

Supplement: Supplementary file 5 — Source Data for Figure 2 [file EMBR-24-e56849-s009.zip › 2C. Image and numerical data Micr.image+quantif/2C. Micr.image/pTrc200/pTrc200_3.tif]

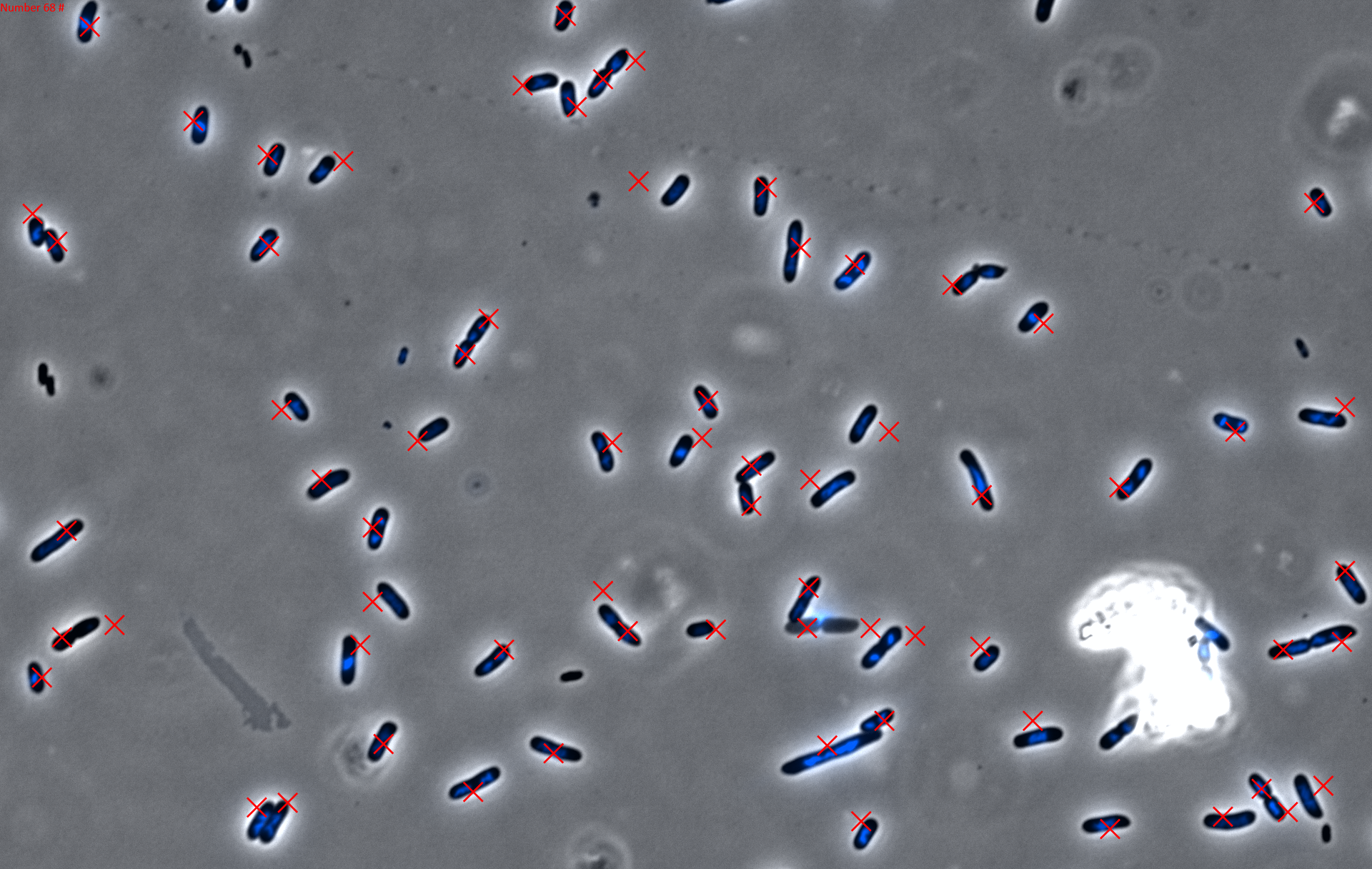

Supplement: Supplementary file 5 — Source Data for Figure 2 [file EMBR-24-e56849-s009.zip › 2C. Image and numerical data Micr.image+quantif/2C. Micr.image/pTrc200/pTrc200_6.tif]

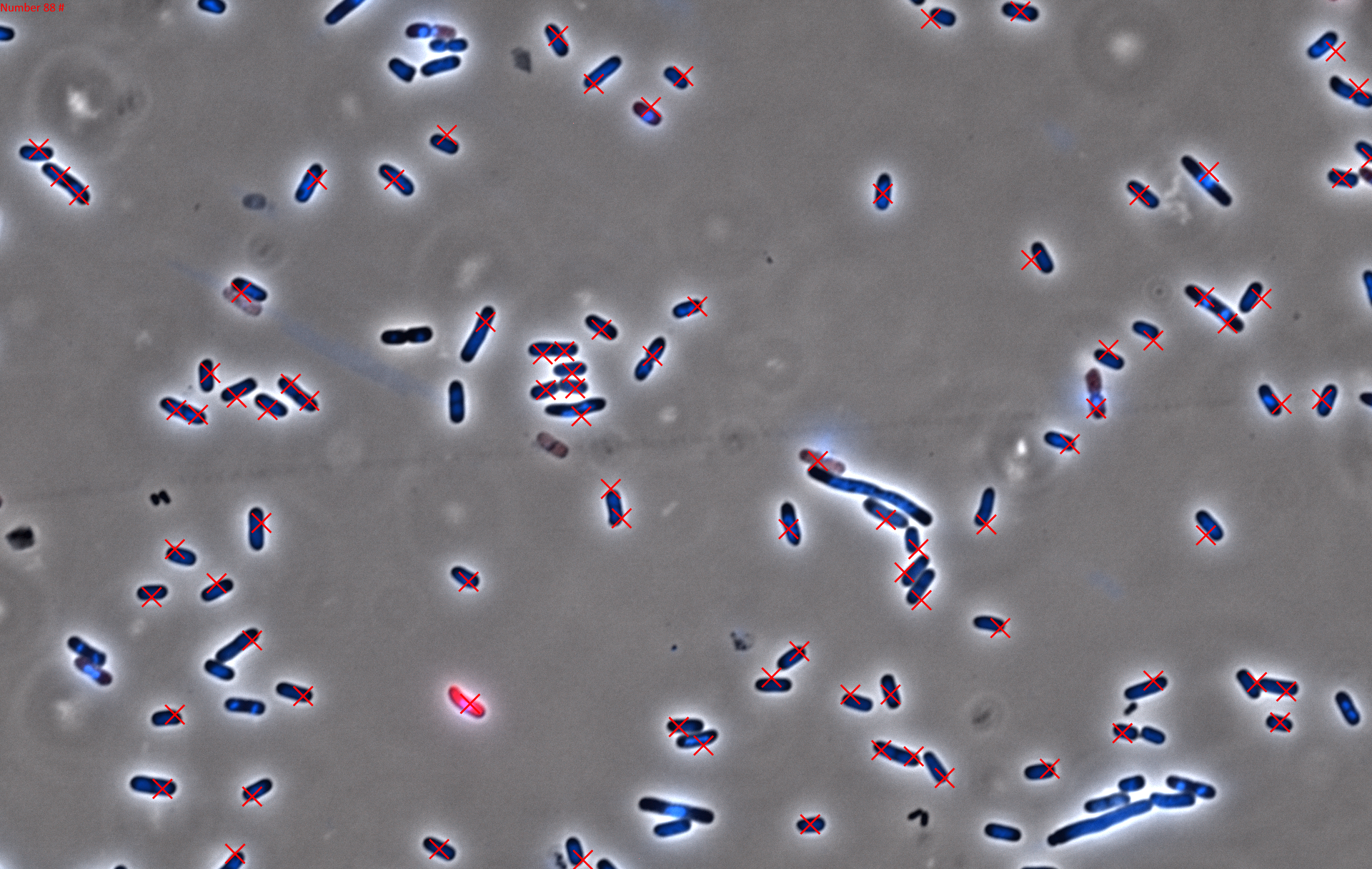

Supplement: Supplementary file 5 — Source Data for Figure 2 [file EMBR-24-e56849-s009.zip › 2C. Image and numerical data Micr.image+quantif/2C. Micr.image/pTrc200/pTrc200_4.tif]

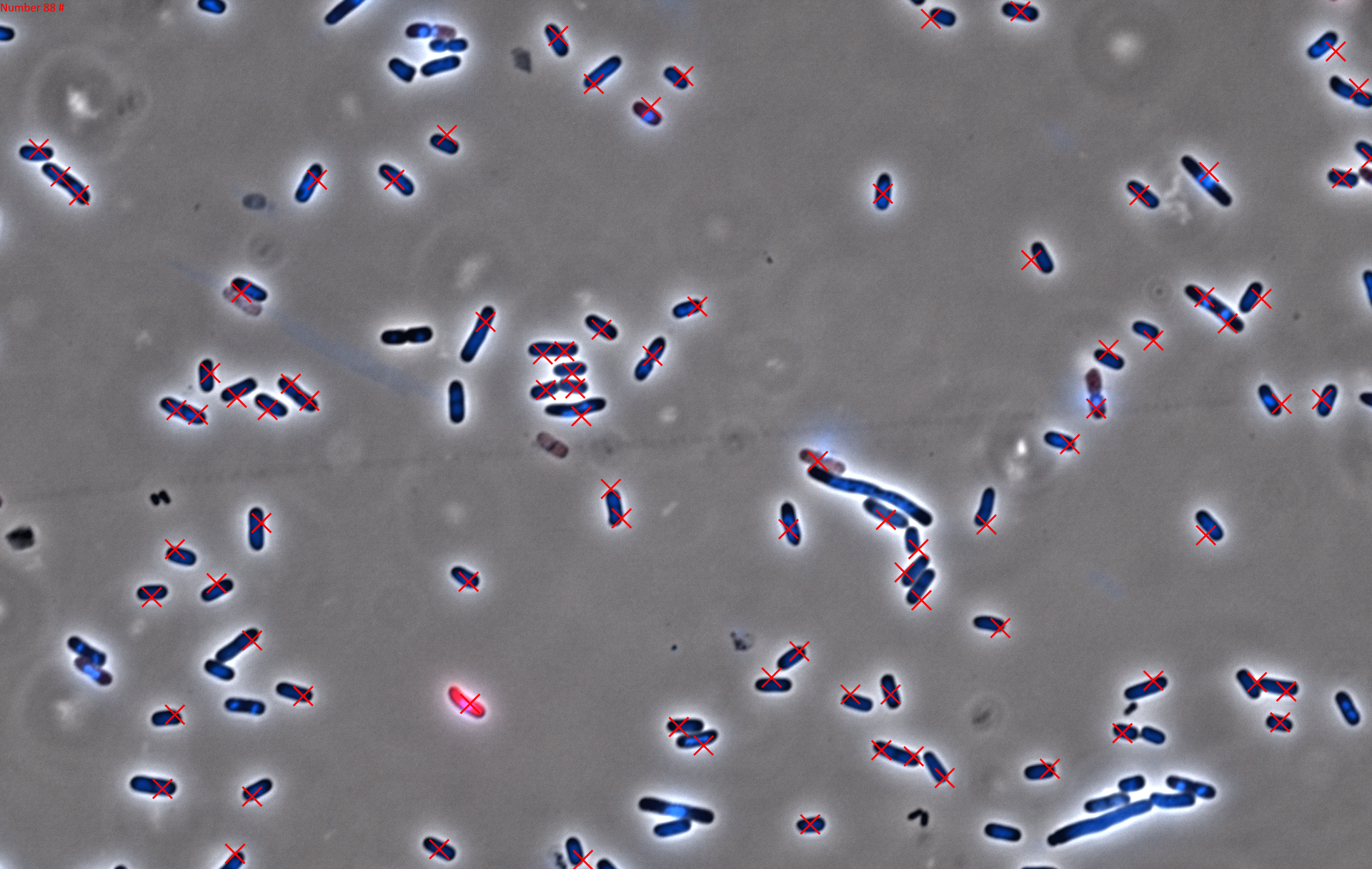

Supplement: Supplementary file 5 — Source Data for Figure 2 [file EMBR-24-e56849-s009.zip › 2C. Image and numerical data Micr.image+quantif/2C. Micr.image/pTrc200/pTrc200_5.tif]

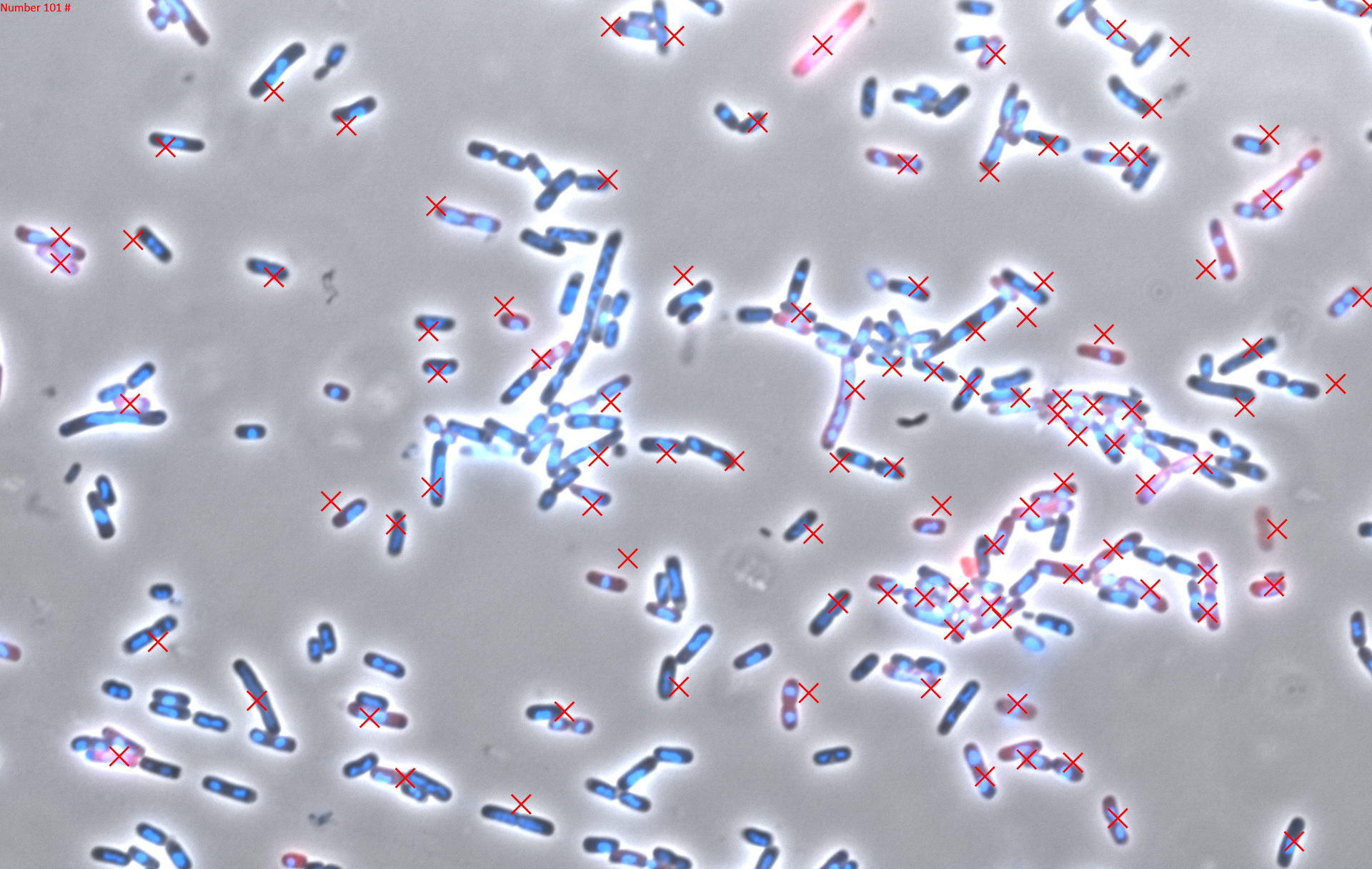

Supplement: Supplementary file 5 — Source Data for Figure 2 [file EMBR-24-e56849-s009.zip › 2C. Image and numerical data Micr.image+quantif/2C. Micr.image/Tde1(M)/Tde1(M)_1.tif]

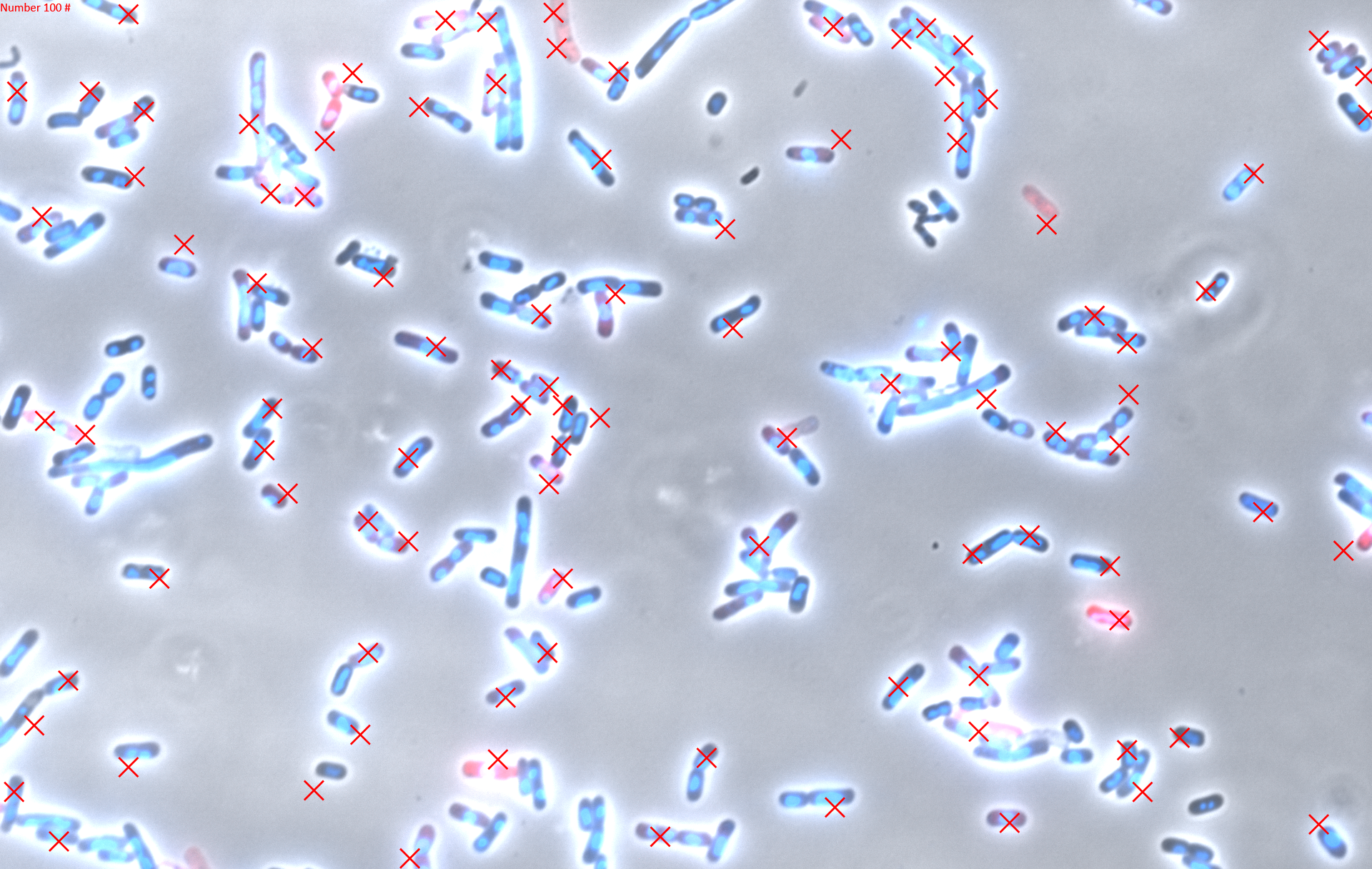

Supplement: Supplementary file 5 — Source Data for Figure 2 [file EMBR-24-e56849-s009.zip › 2C. Image and numerical data Micr.image+quantif/2C. Micr.image/Tde1(M)/Tde1(M)_2.tif]

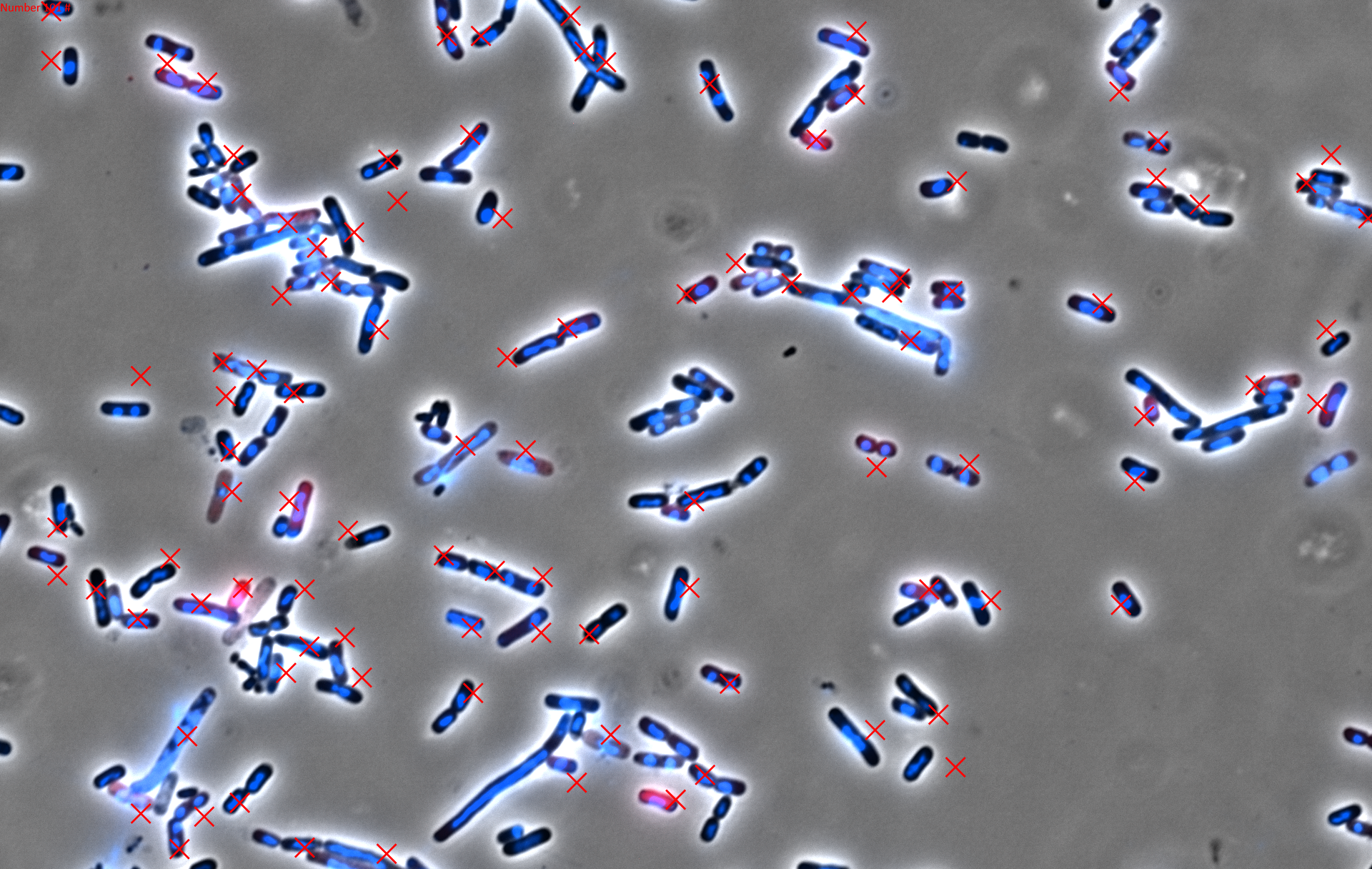

Supplement: Supplementary file 5 — Source Data for Figure 2 [file EMBR-24-e56849-s009.zip › 2C. Image and numerical data Micr.image+quantif/2C. Micr.image/Tde1(M)/Tde1(M)_3.tif]

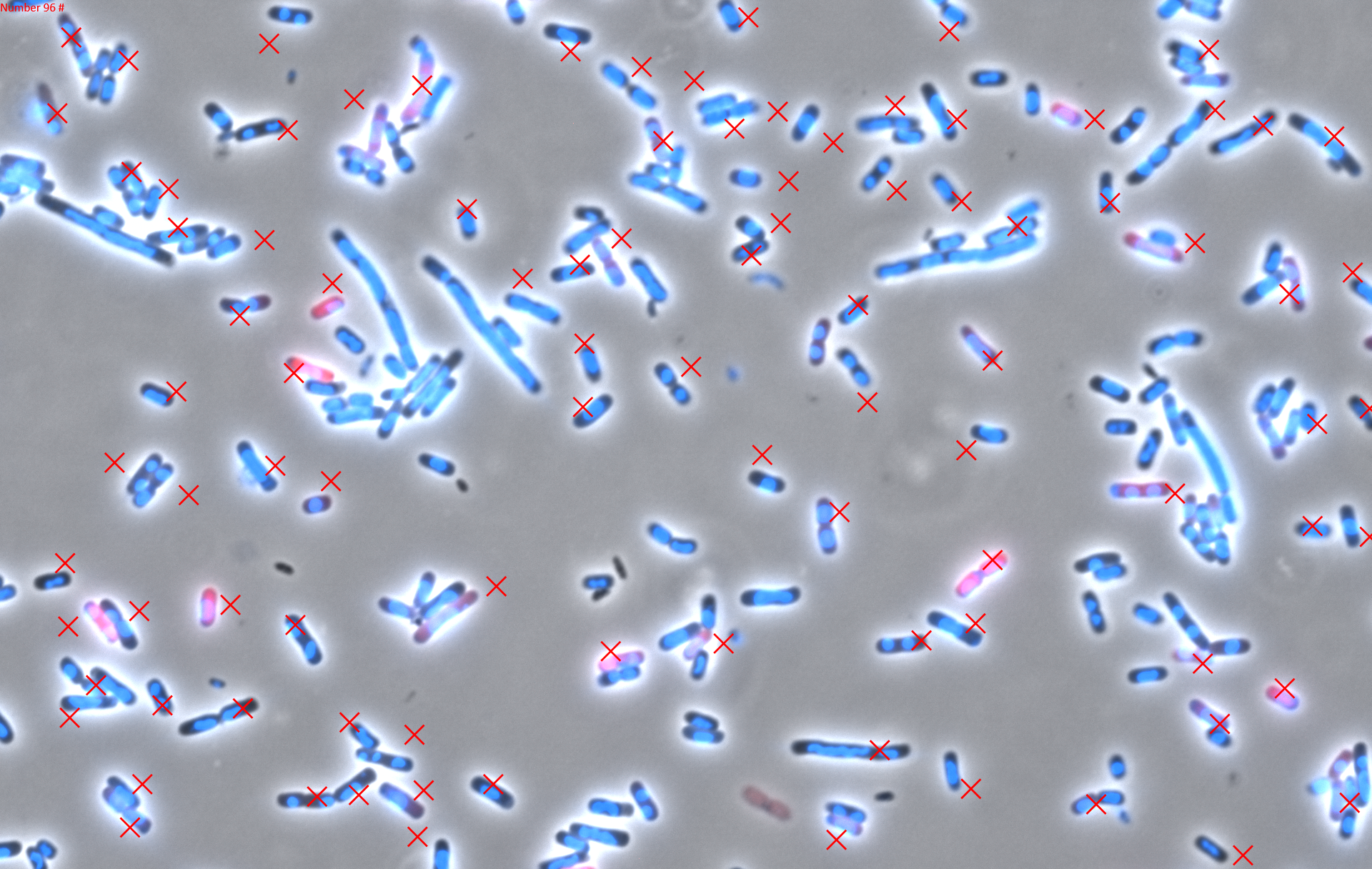

Supplement: Supplementary file 5 — Source Data for Figure 2 [file EMBR-24-e56849-s009.zip › 2C. Image and numerical data Micr.image+quantif/2C. Micr.image/Tde1(M)/Tde1(M)_6.tif]

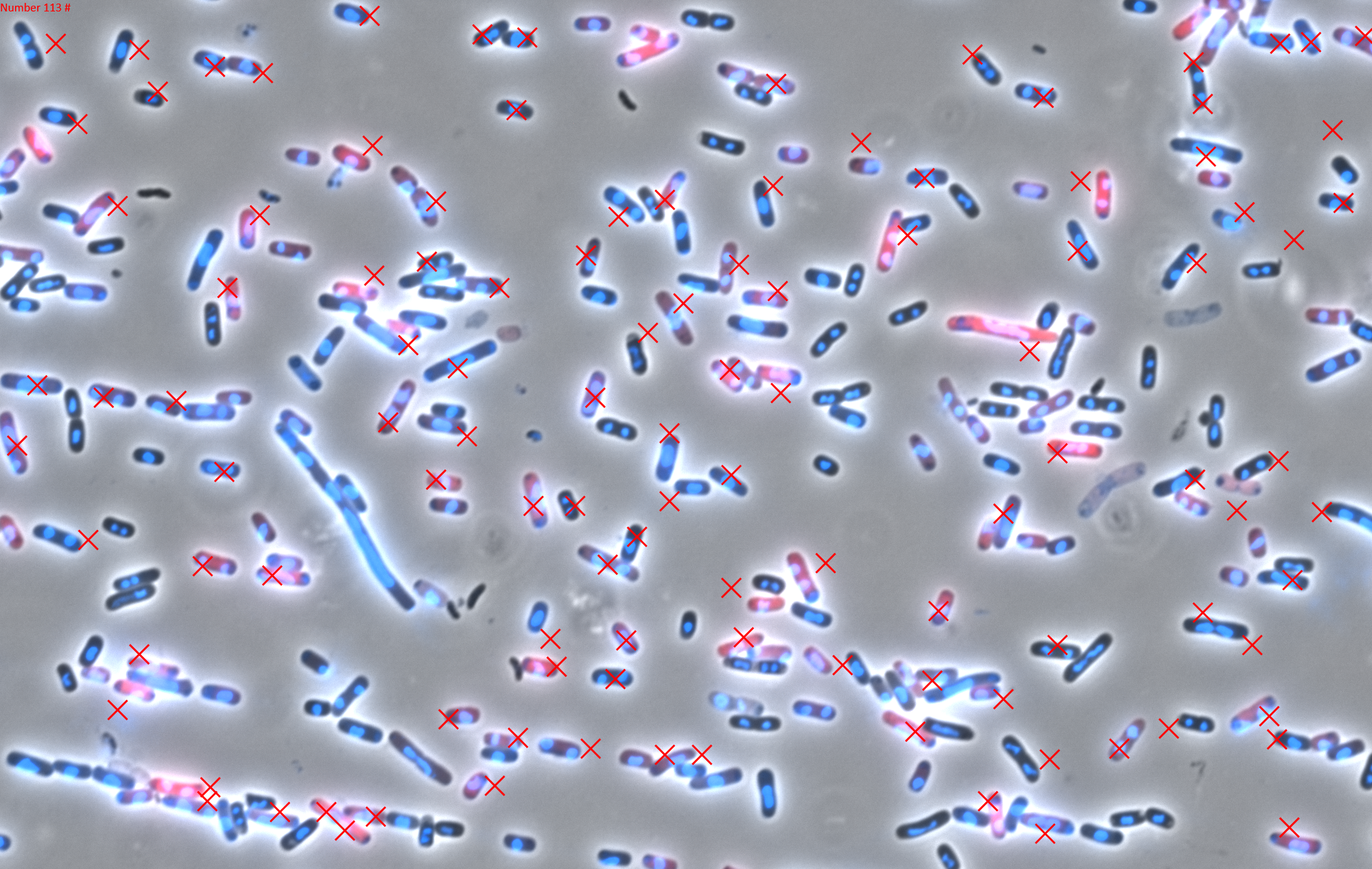

Supplement: Supplementary file 5 — Source Data for Figure 2 [file EMBR-24-e56849-s009.zip › 2C. Image and numerical data Micr.image+quantif/2C. Micr.image/Tde1(M)/Tde1(M)_4.tif]

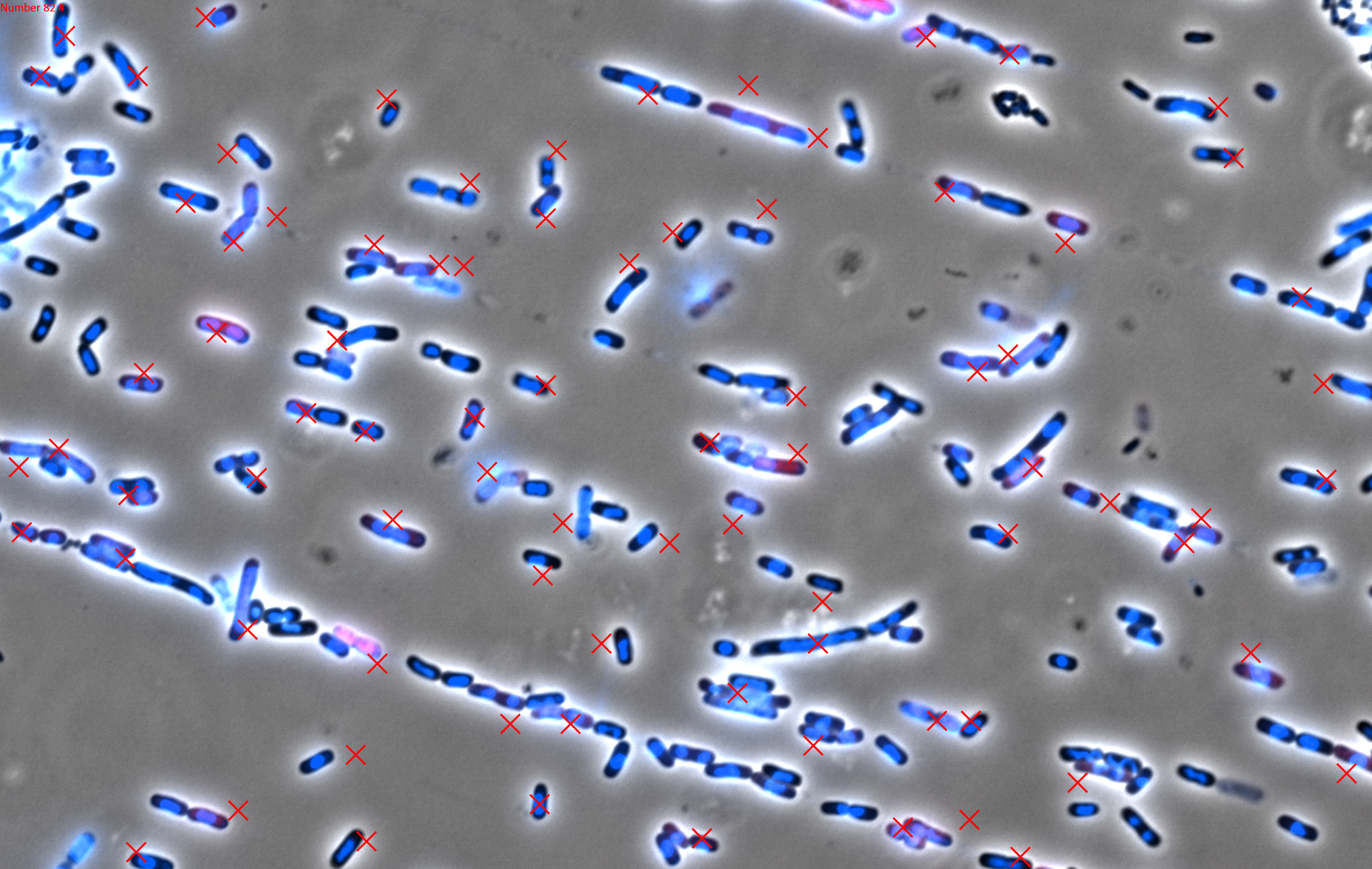

Supplement: Supplementary file 5 — Source Data for Figure 2 [file EMBR-24-e56849-s009.zip › 2C. Image and numerical data Micr.image+quantif/2C. Micr.image/Tde1(M)/Tde1(M)_5.tif]

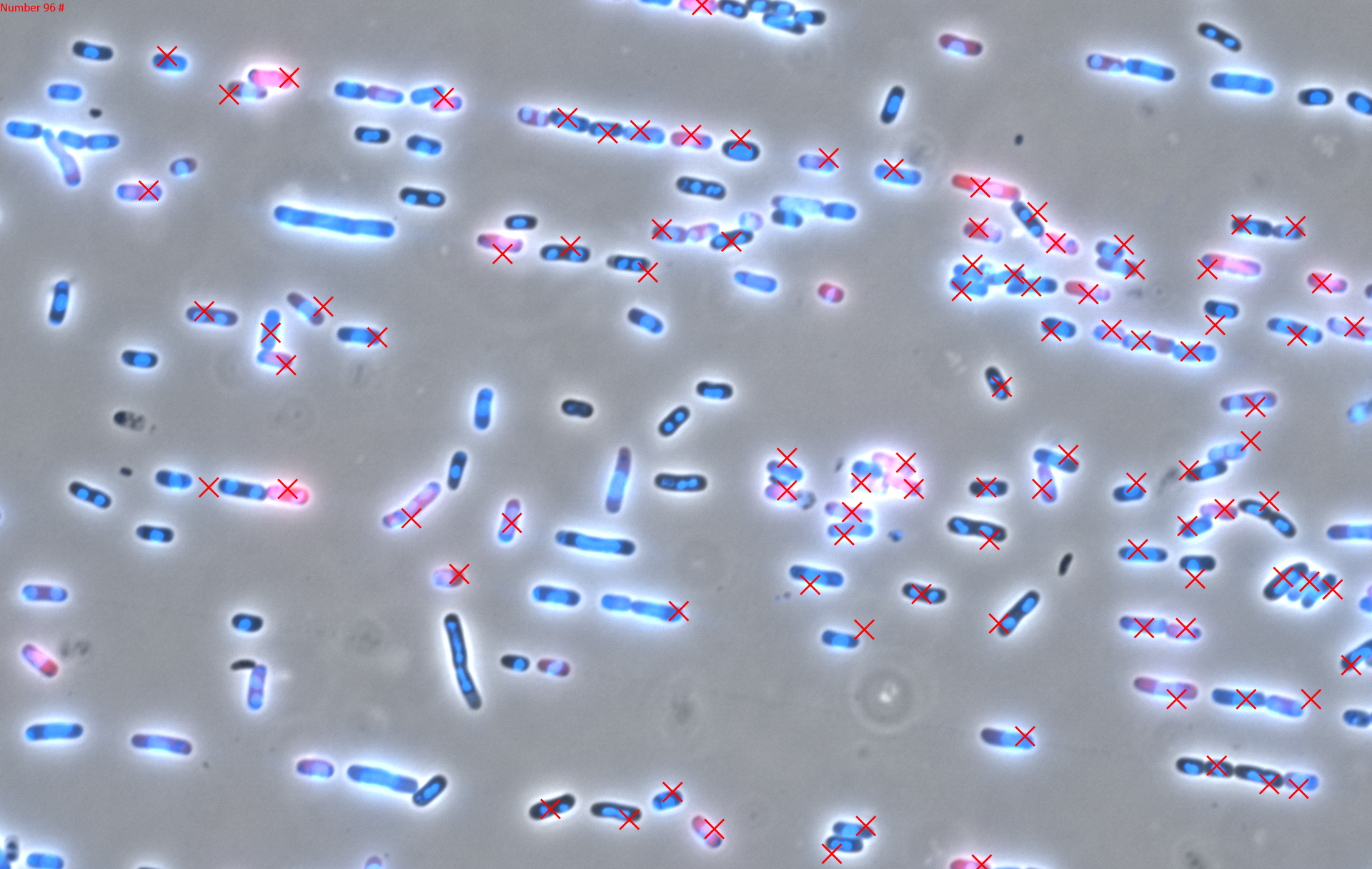

Supplement: Supplementary file 5 — Source Data for Figure 2 [file EMBR-24-e56849-s009.zip › 2C. Image and numerical data Micr.image+quantif/2C. Micr.image/N-Tde1/N-Tde1_1.tif]

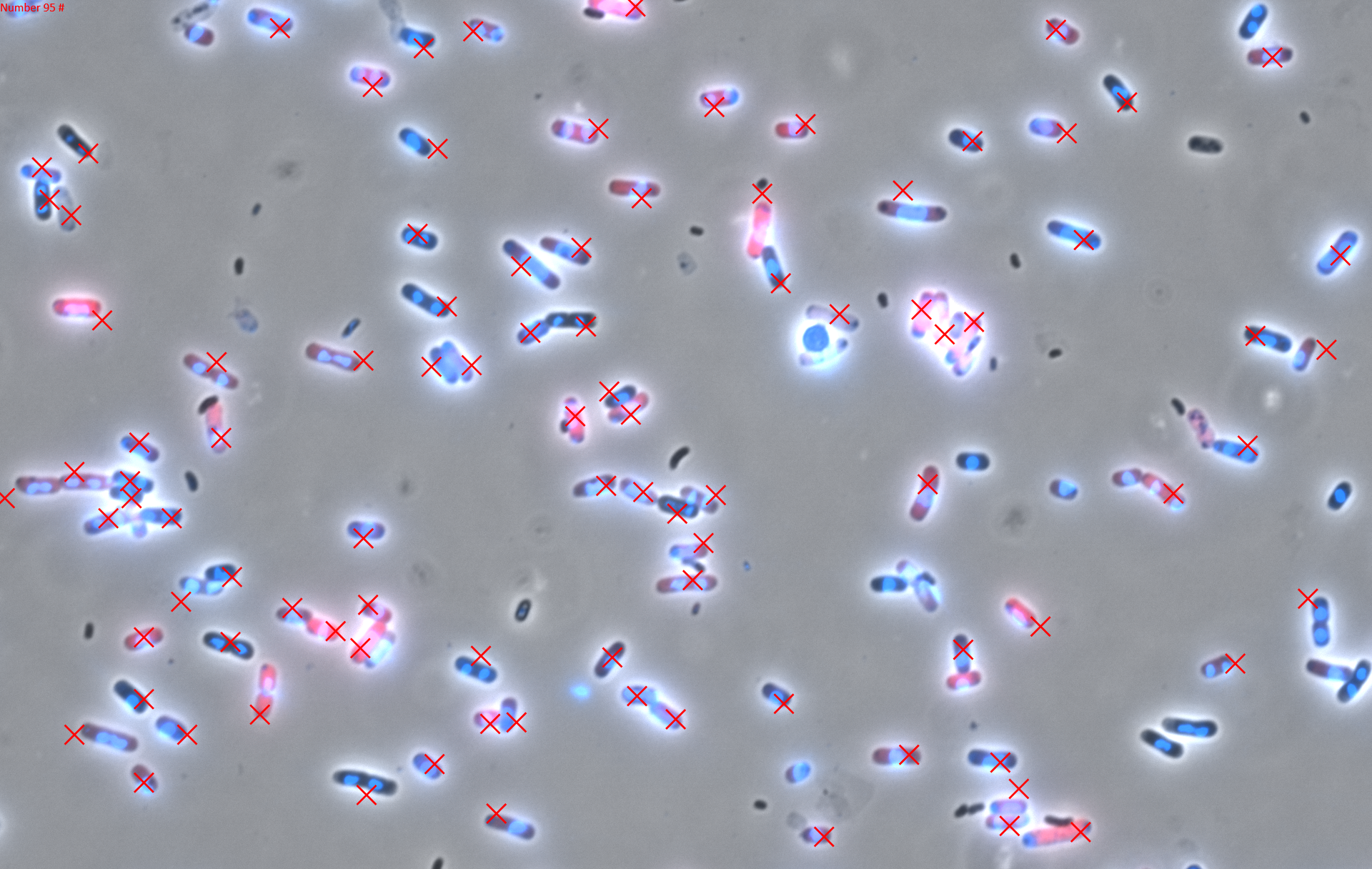

Supplement: Supplementary file 5 — Source Data for Figure 2 [file EMBR-24-e56849-s009.zip › 2C. Image and numerical data Micr.image+quantif/2C. Micr.image/N-Tde1/N-Tde1_2.tif]

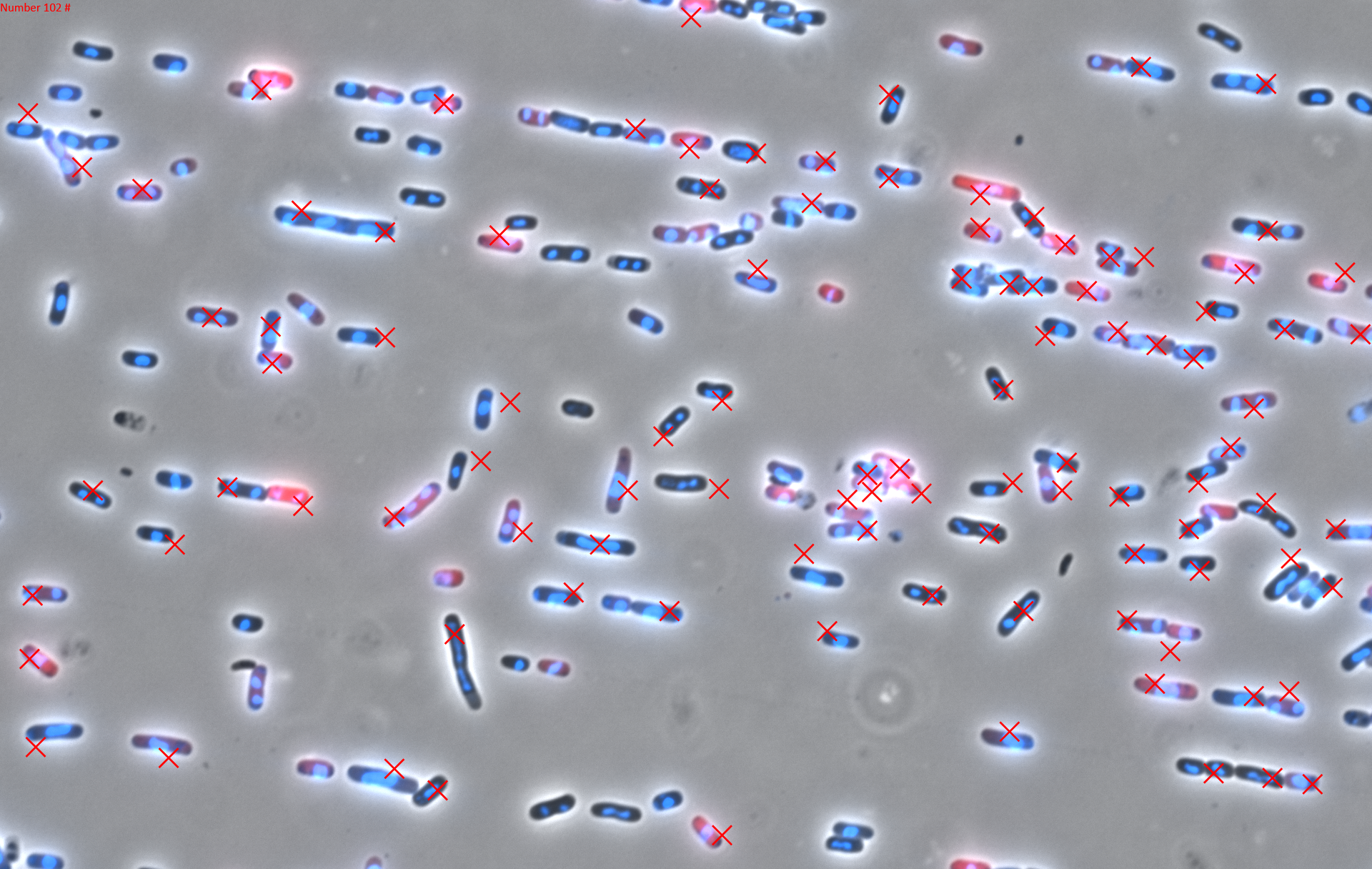

Supplement: Supplementary file 5 — Source Data for Figure 2 [file EMBR-24-e56849-s009.zip › 2C. Image and numerical data Micr.image+quantif/2C. Micr.image/N-Tde1/N-Tde1_3.tif]

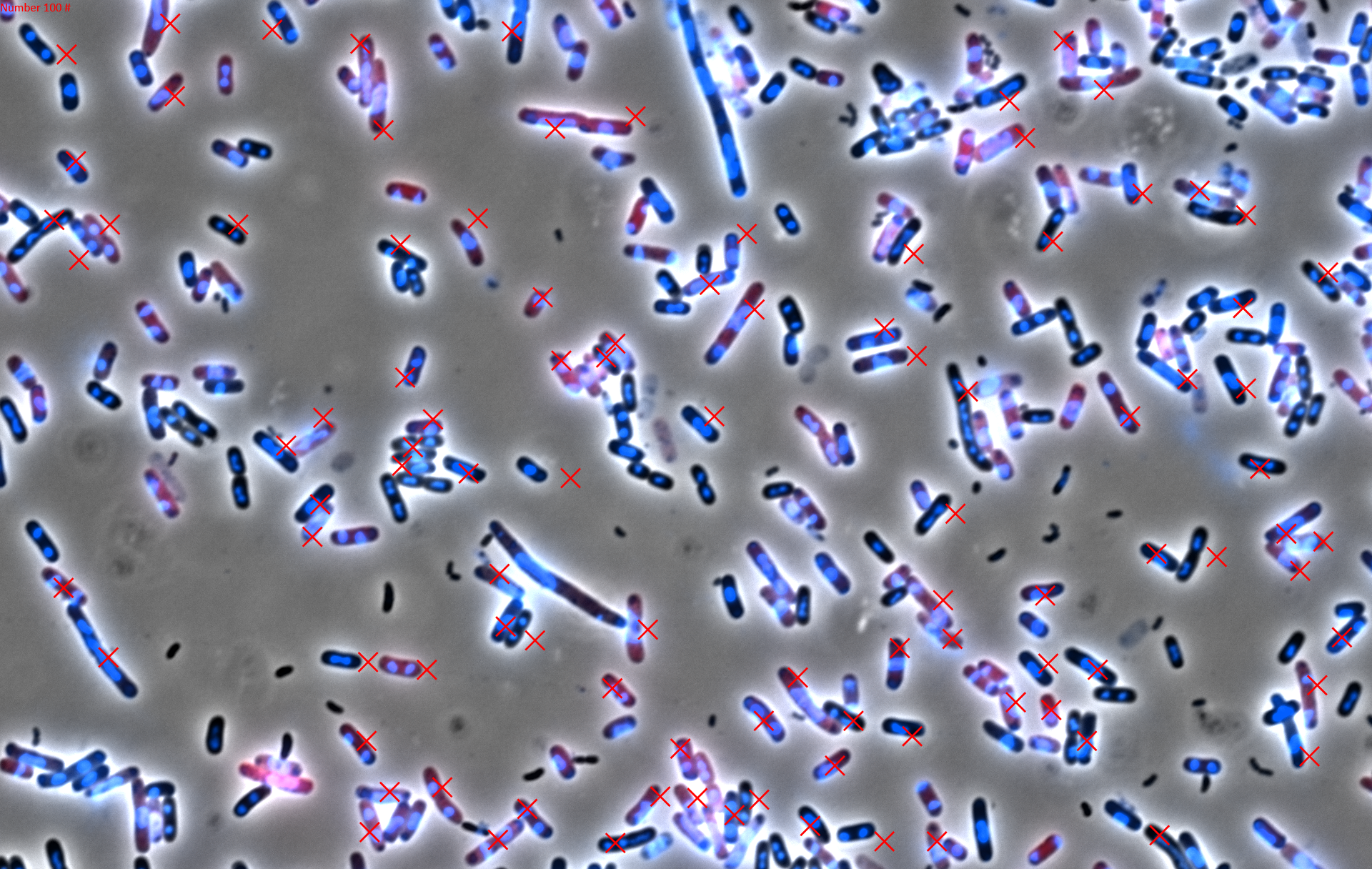

Supplement: Supplementary file 5 — Source Data for Figure 2 [file EMBR-24-e56849-s009.zip › 2C. Image and numerical data Micr.image+quantif/2C. Micr.image/N-Tde1/N-Tde1_6.tif]

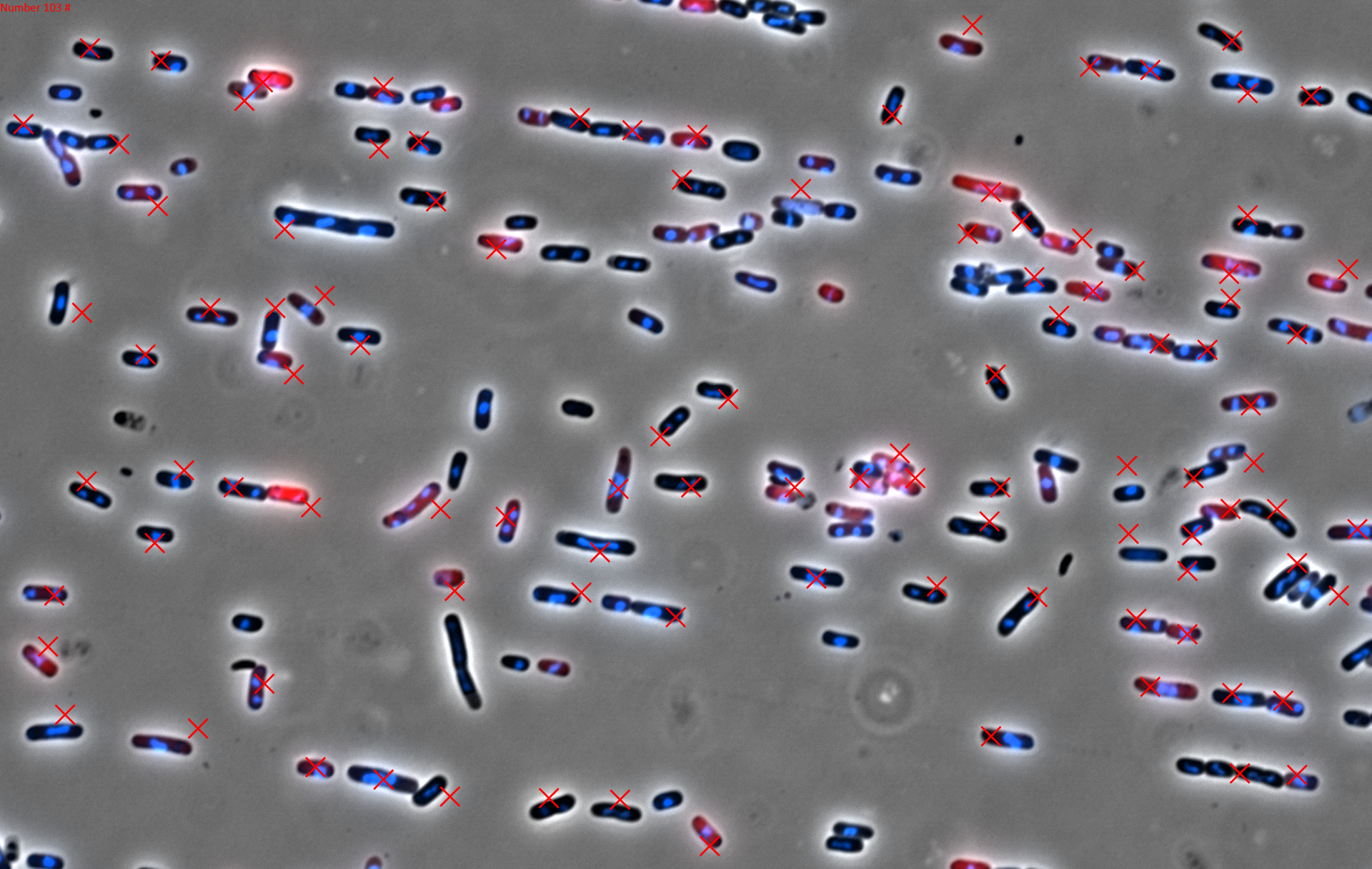

Supplement: Supplementary file 5 — Source Data for Figure 2 [file EMBR-24-e56849-s009.zip › 2C. Image and numerical data Micr.image+quantif/2C. Micr.image/N-Tde1/N-Tde1_4.tif]

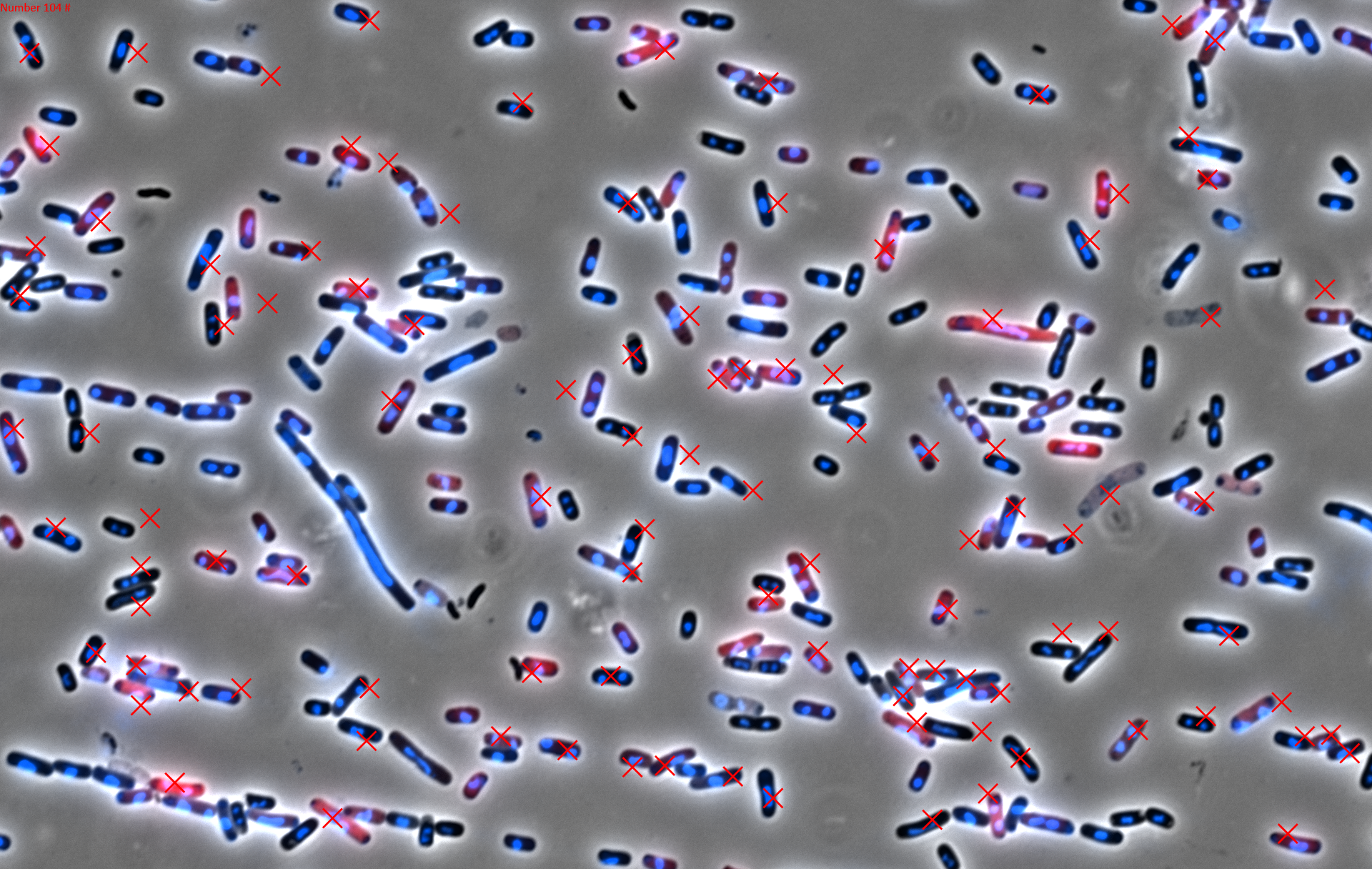

Supplement: Supplementary file 5 — Source Data for Figure 2 [file EMBR-24-e56849-s009.zip › 2C. Image and numerical data Micr.image+quantif/2C. Micr.image/N-Tde1/N-tde1_5.tif]

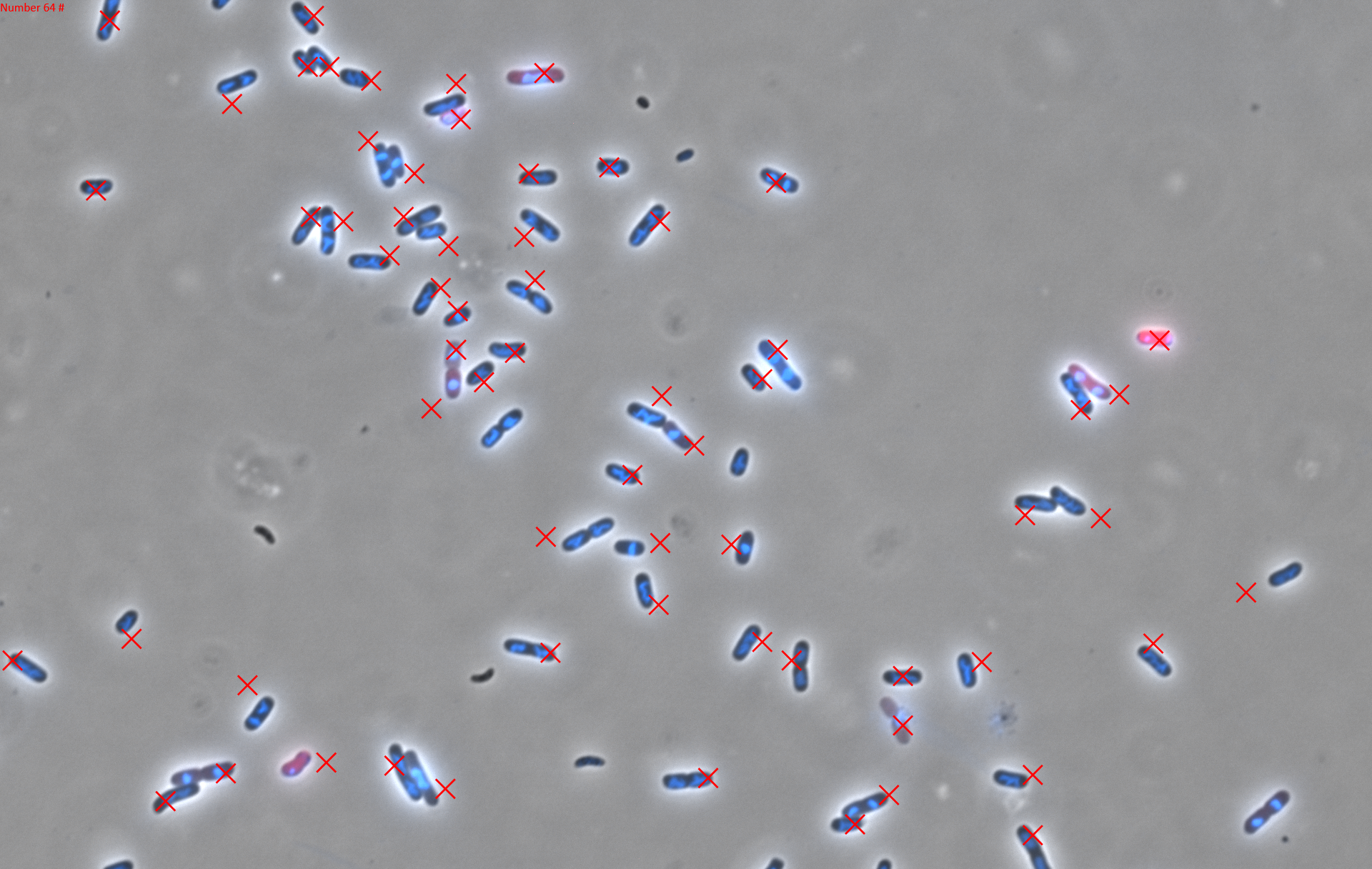

Supplement: Supplementary file 5 — Source Data for Figure 2 [file EMBR-24-e56849-s009.zip › 2C. Image and numerical data Micr.image+quantif/2C. Micr.image/N-Tde1GLGL/N-Tde1GLGL_6.tif]

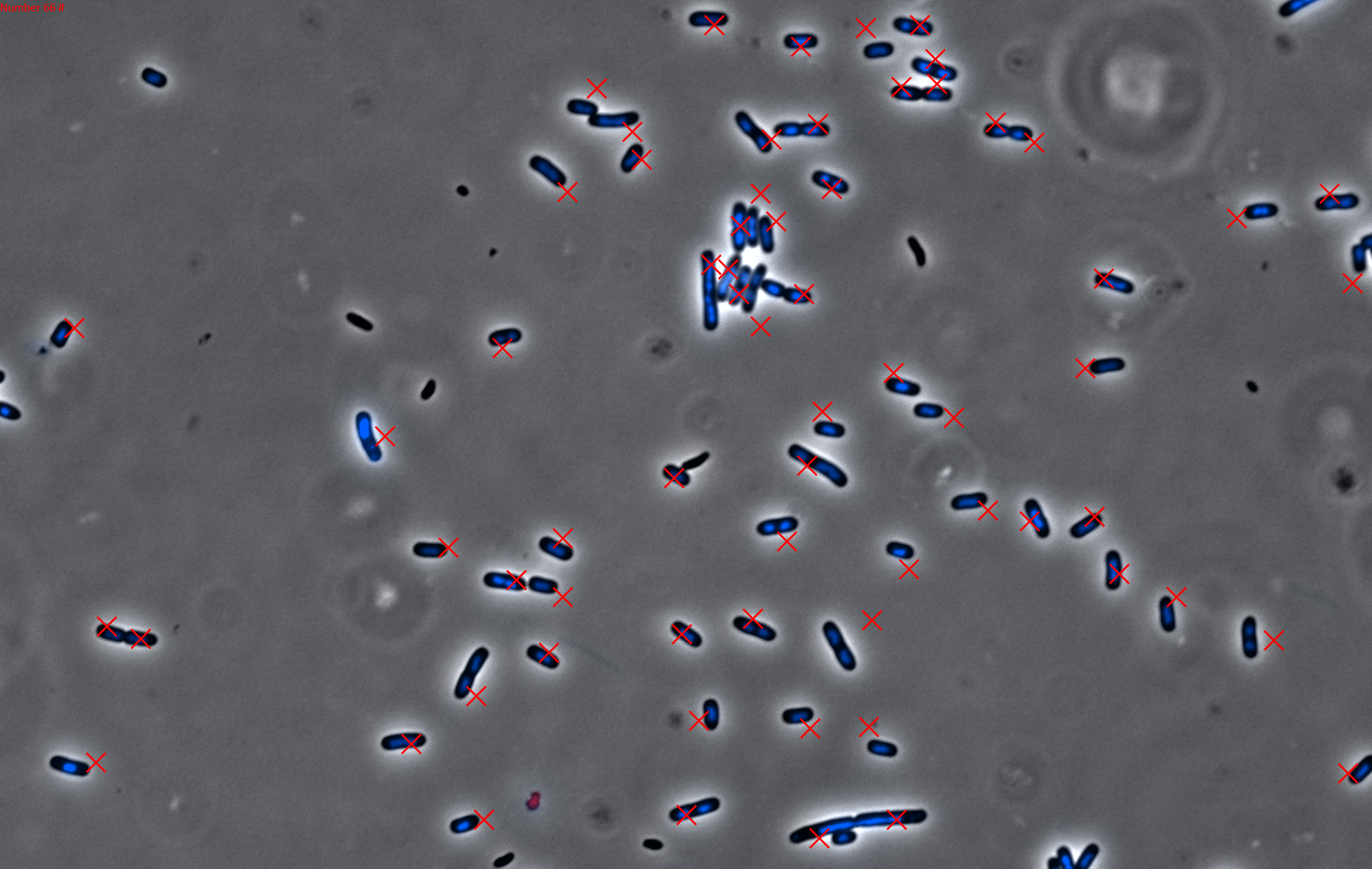

Supplement: Supplementary file 5 — Source Data for Figure 2 [file EMBR-24-e56849-s009.zip › 2C. Image and numerical data Micr.image+quantif/2C. Micr.image/N-Tde1GLGL/N-Tde1GLGL_4.tif]

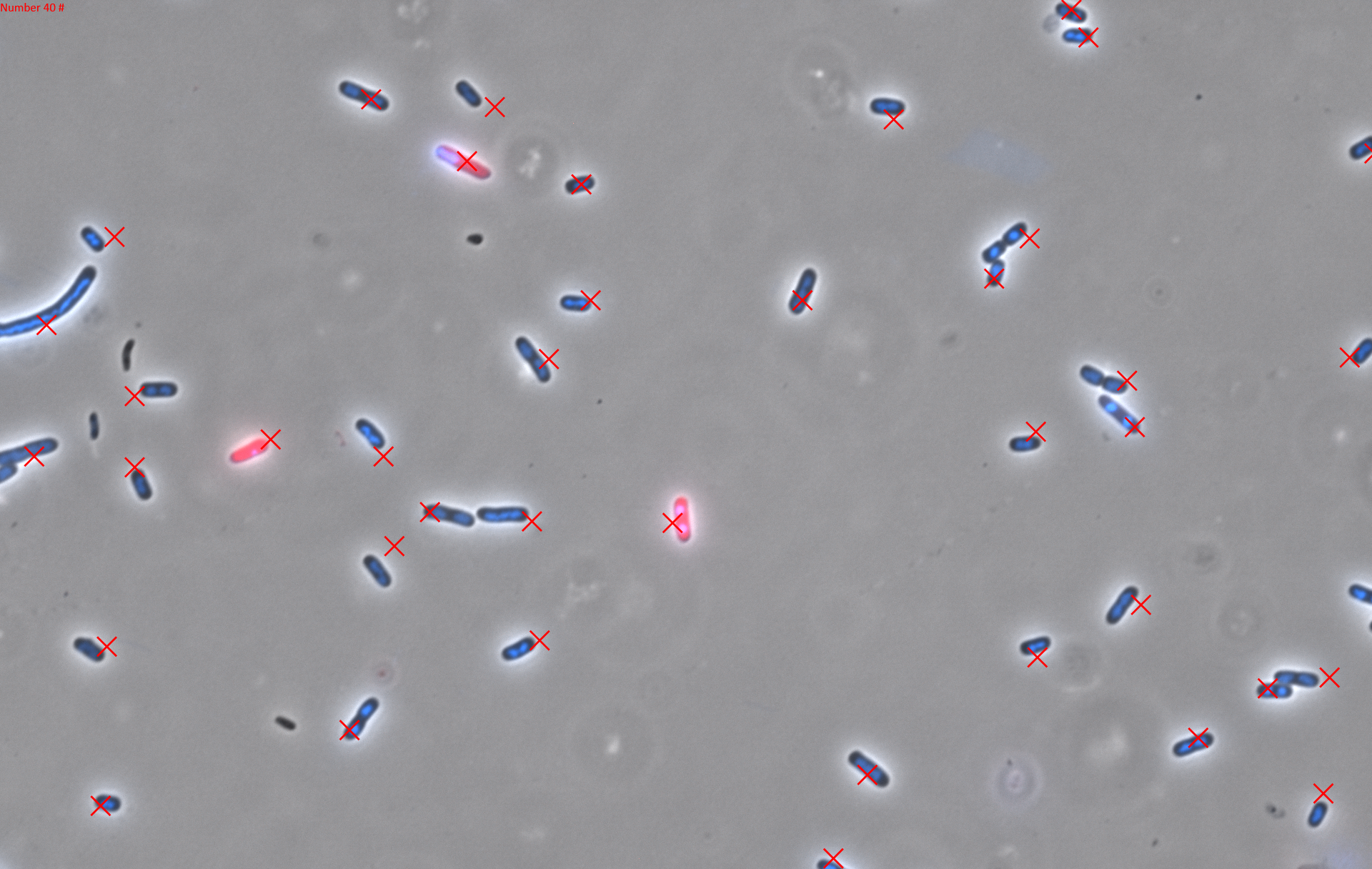

Supplement: Supplementary file 5 — Source Data for Figure 2 [file EMBR-24-e56849-s009.zip › 2C. Image and numerical data Micr.image+quantif/2C. Micr.image/N-Tde1GLGL/N-Tde1GLGL_5.tif]

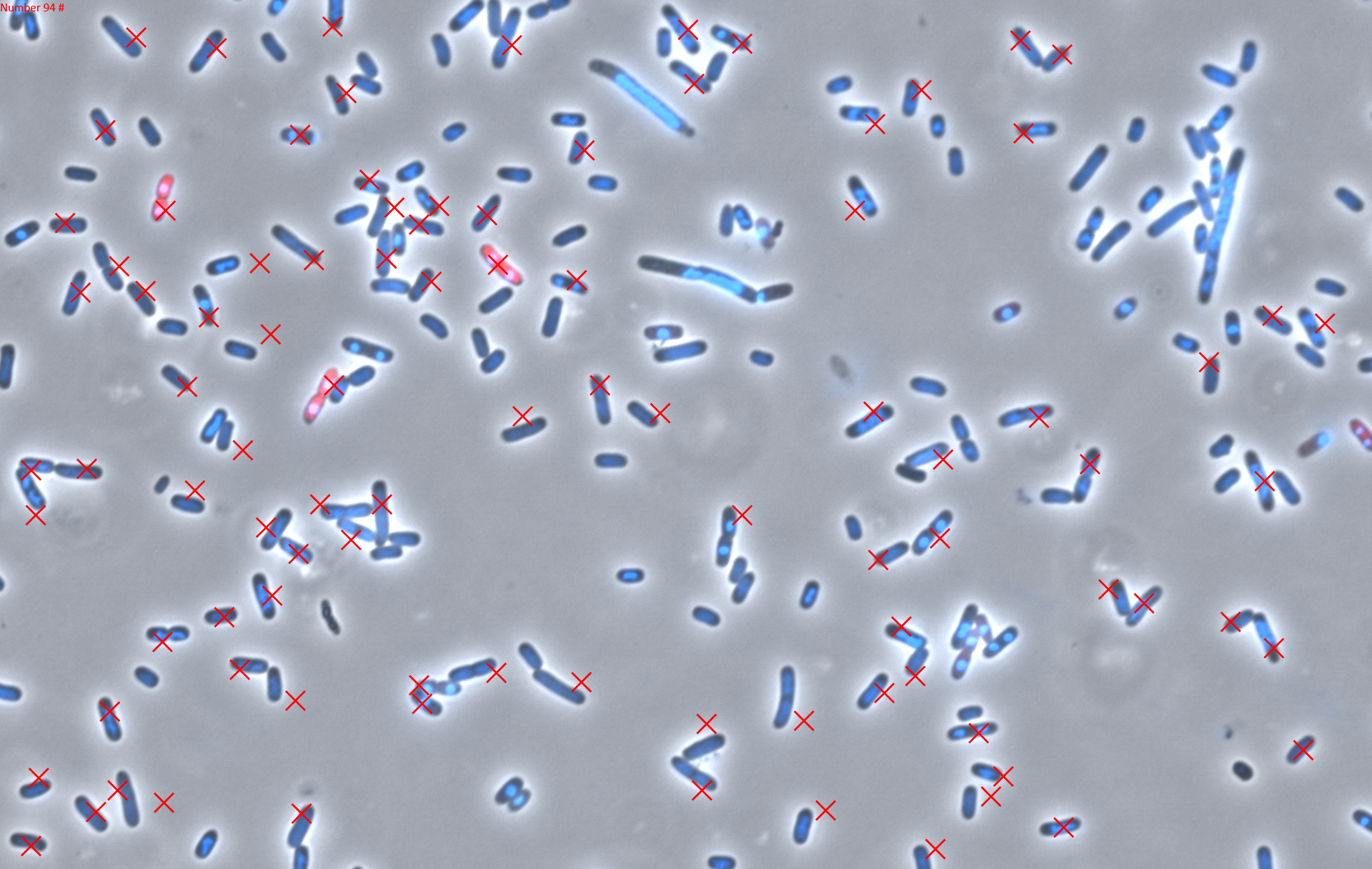

Supplement: Supplementary file 5 — Source Data for Figure 2 [file EMBR-24-e56849-s009.zip › 2C. Image and numerical data Micr.image+quantif/2C. Micr.image/N-Tde1GLGL/N-Tde1GLGL_1.tif]

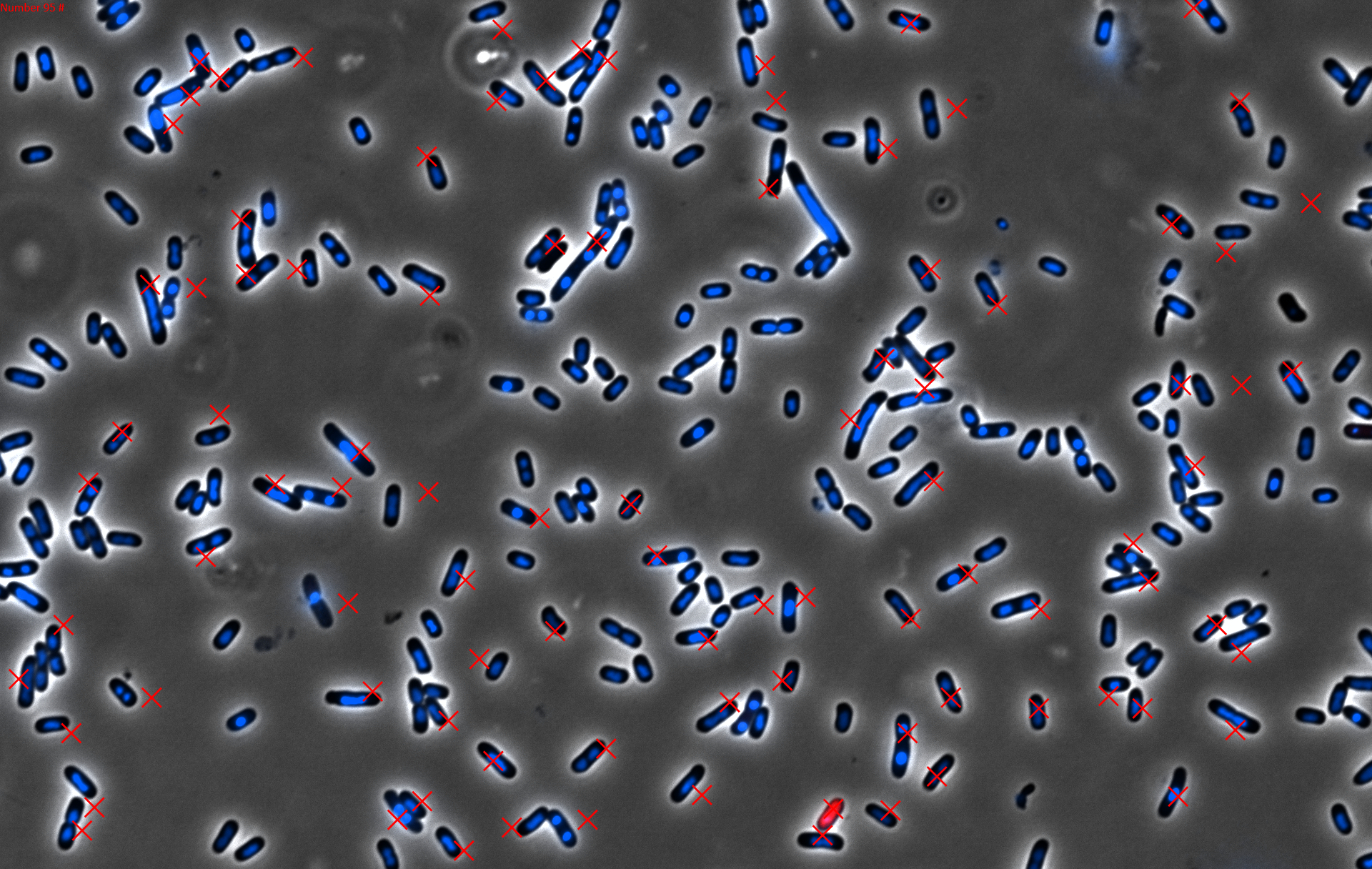

Supplement: Supplementary file 5 — Source Data for Figure 2 [file EMBR-24-e56849-s009.zip › 2C. Image and numerical data Micr.image+quantif/2C. Micr.image/N-Tde1GLGL/N-Tde1GLGL_2.tif]

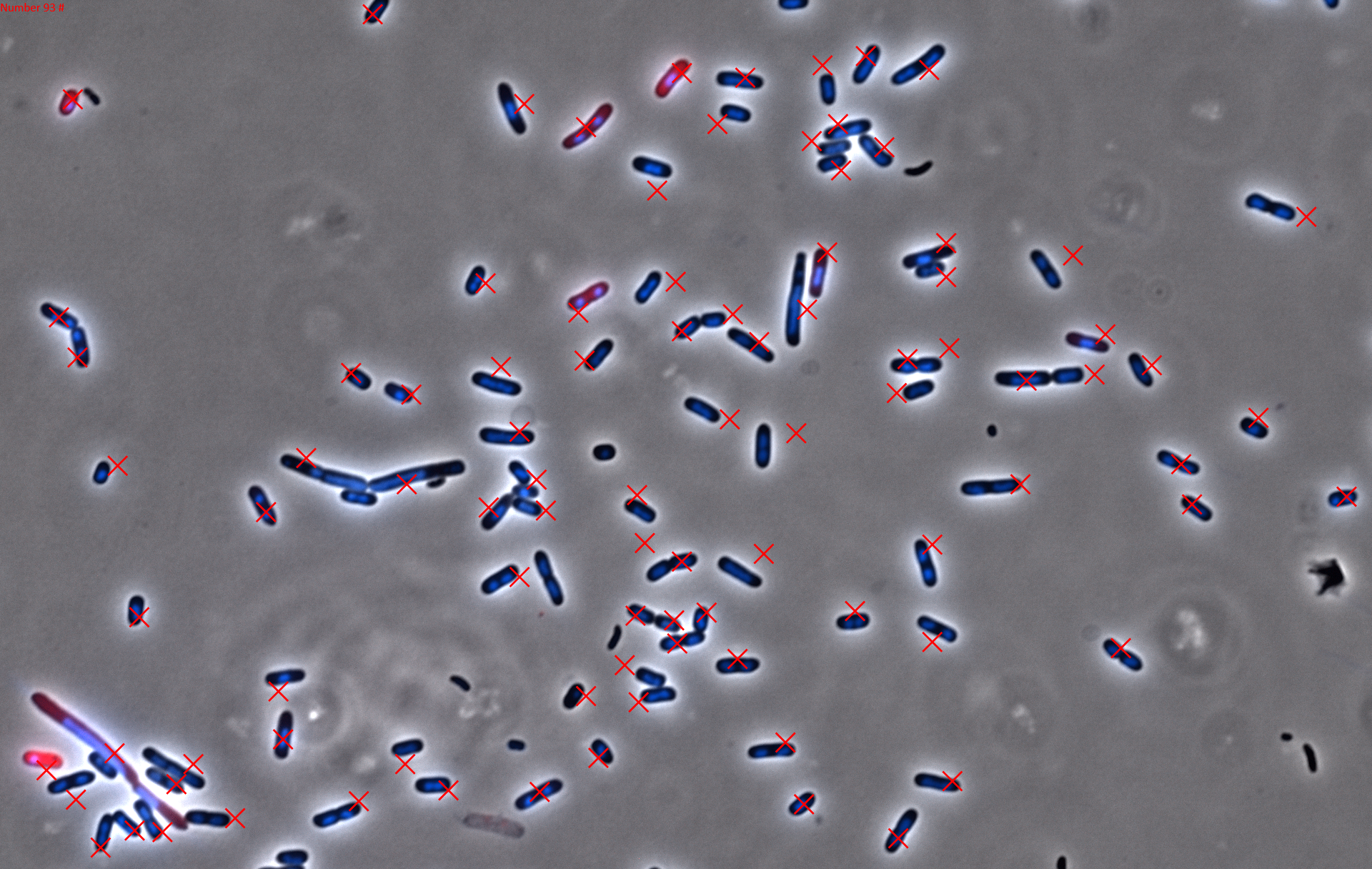

Supplement: Supplementary file 5 — Source Data for Figure 2 [file EMBR-24-e56849-s009.zip › 2C. Image and numerical data Micr.image+quantif/2C. Micr.image/N-Tde1GLGL/N-tde1GLGL_3.tif]

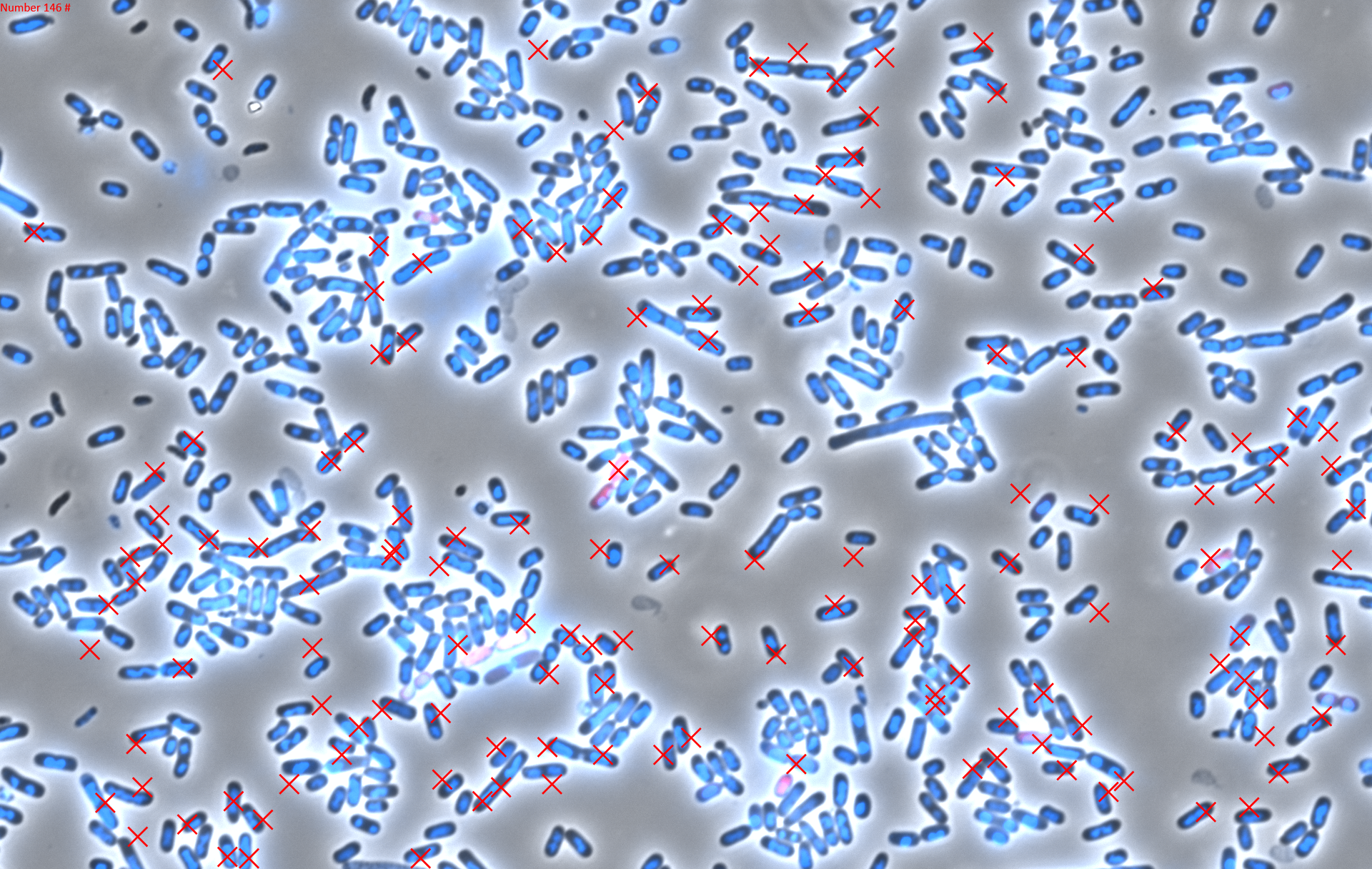

Supplement: Supplementary file 5 — Source Data for Figure 2 [file EMBR-24-e56849-s009.zip › 2C. Image and numerical data Micr.image+quantif/2C. Micr.image/Tde1(M)GLGL/Tde1(M)glgl_1.tif]

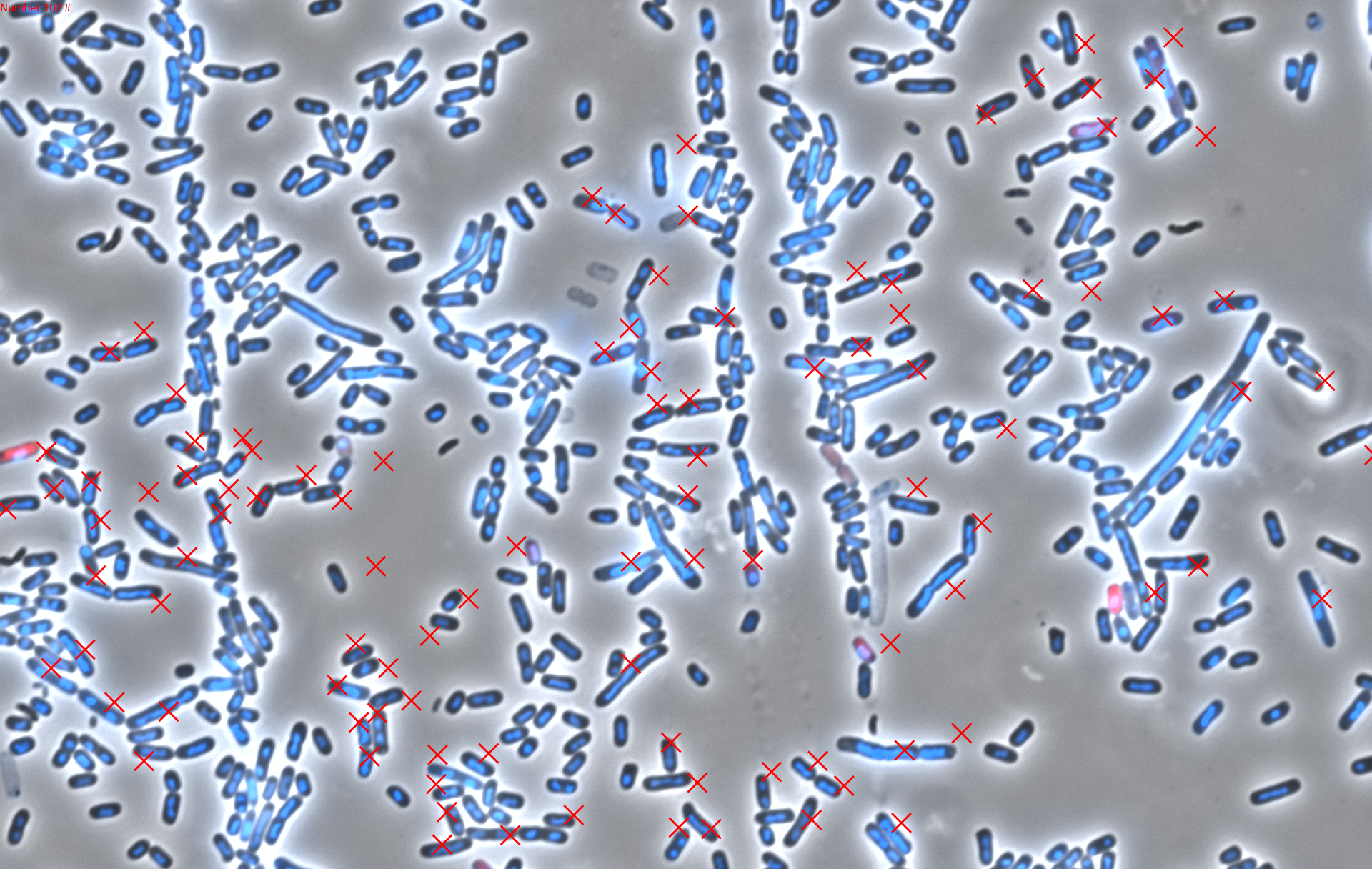

Supplement: Supplementary file 5 — Source Data for Figure 2 [file EMBR-24-e56849-s009.zip › 2C. Image and numerical data Micr.image+quantif/2C. Micr.image/Tde1(M)GLGL/Tde1(M)GLGL_3.tif]

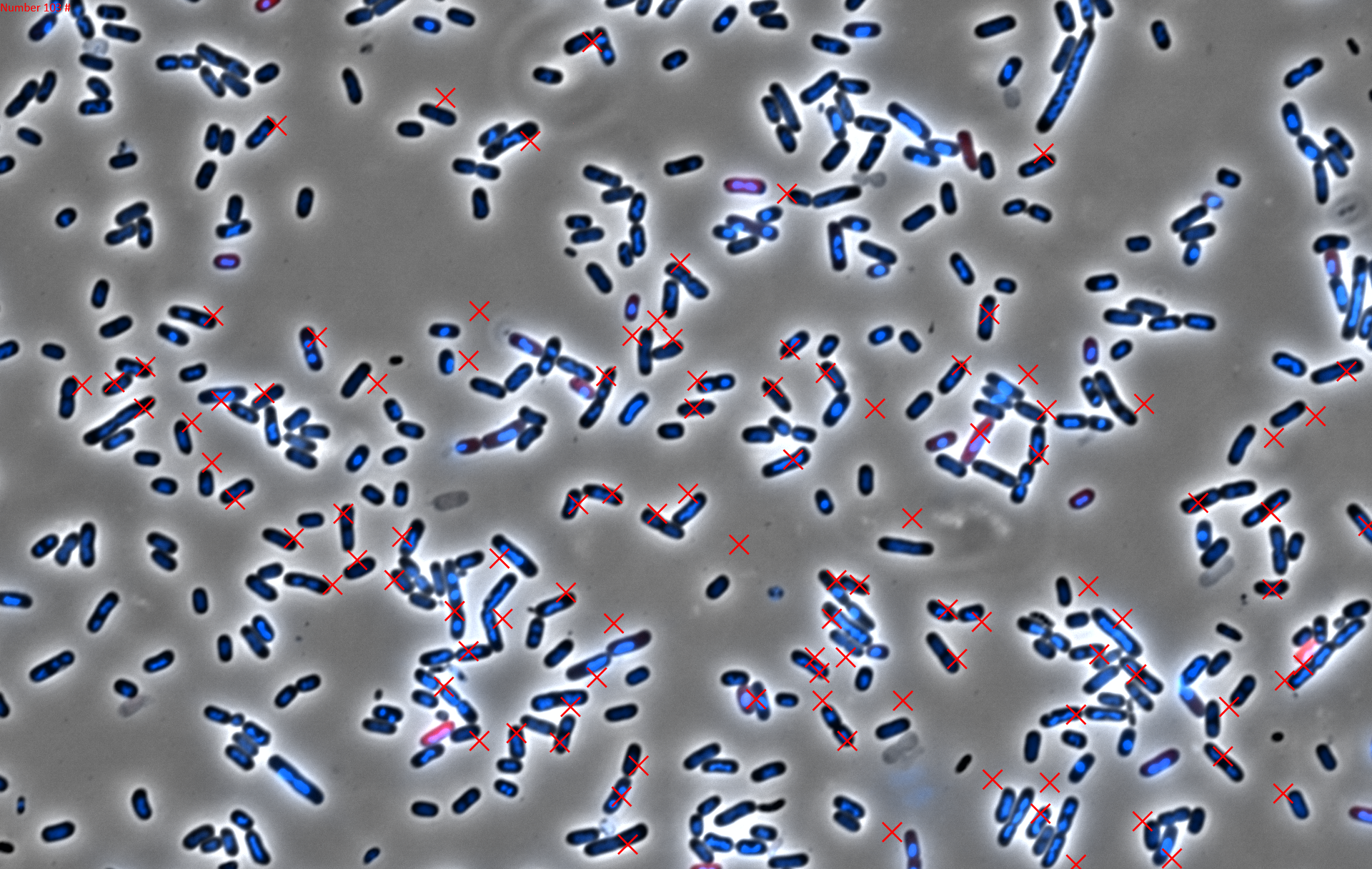

Supplement: Supplementary file 5 — Source Data for Figure 2 [file EMBR-24-e56849-s009.zip › 2C. Image and numerical data Micr.image+quantif/2C. Micr.image/Tde1(M)GLGL/Tde1(M)GLGL_2.tif]

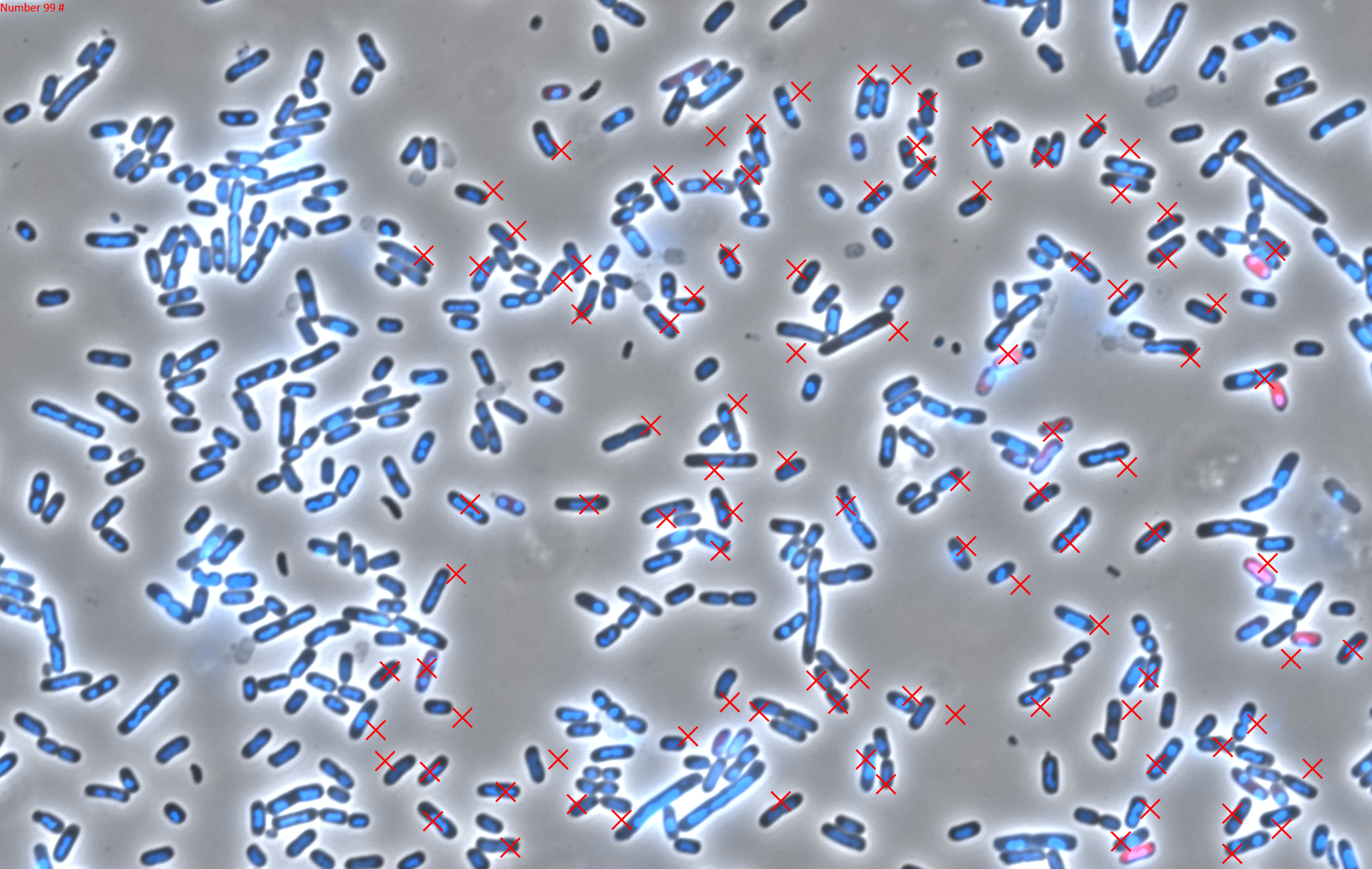

Supplement: Supplementary file 5 — Source Data for Figure 2 [file EMBR-24-e56849-s009.zip › 2C. Image and numerical data Micr.image+quantif/2C. Micr.image/Tde1(M)GLGL/Tde1(M)GLGL_6.tif]

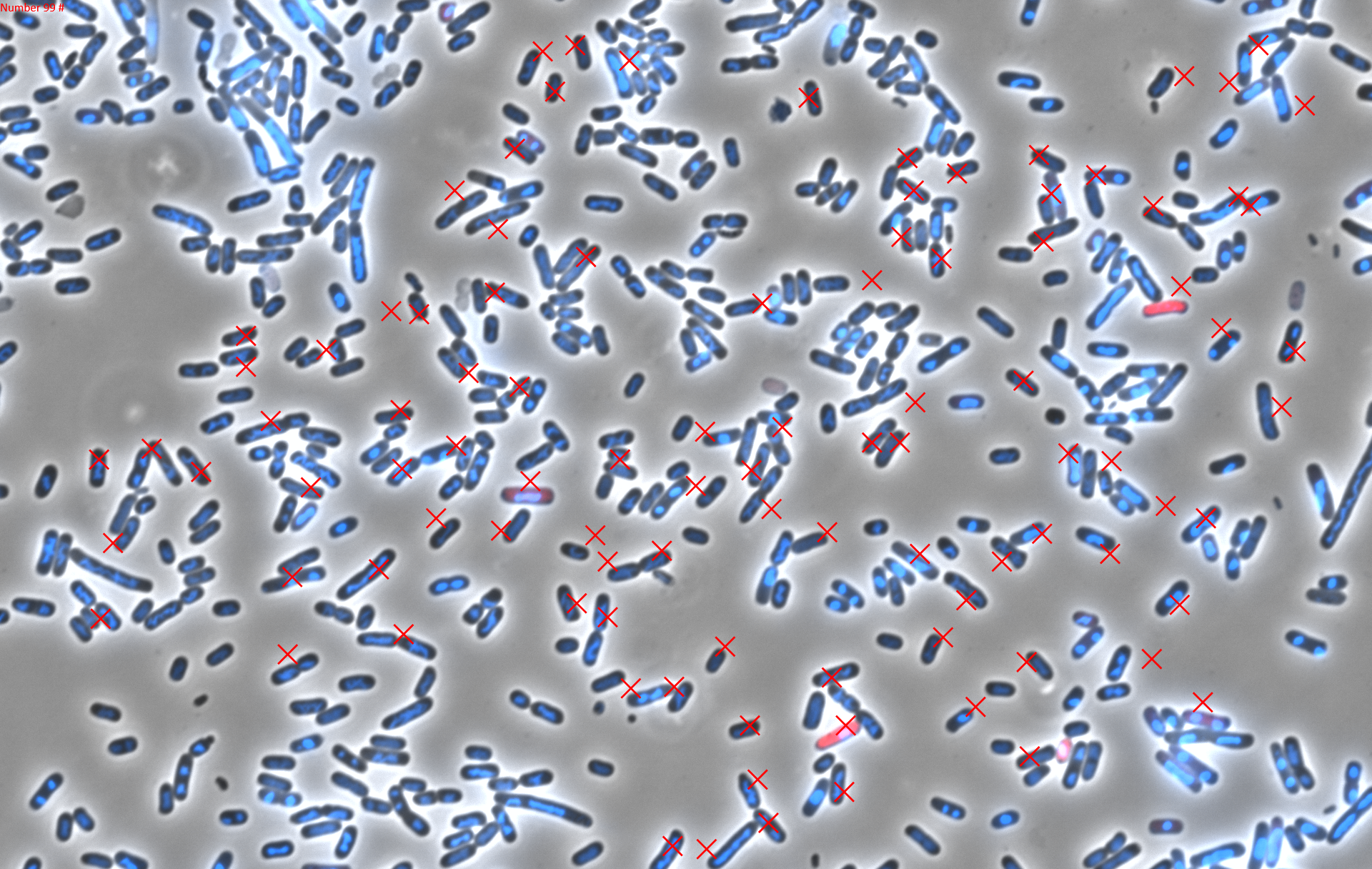

Supplement: Supplementary file 5 — Source Data for Figure 2 [file EMBR-24-e56849-s009.zip › 2C. Image and numerical data Micr.image+quantif/2C. Micr.image/Tde1(M)GLGL/Tde1(M)GLGL_5.tif]

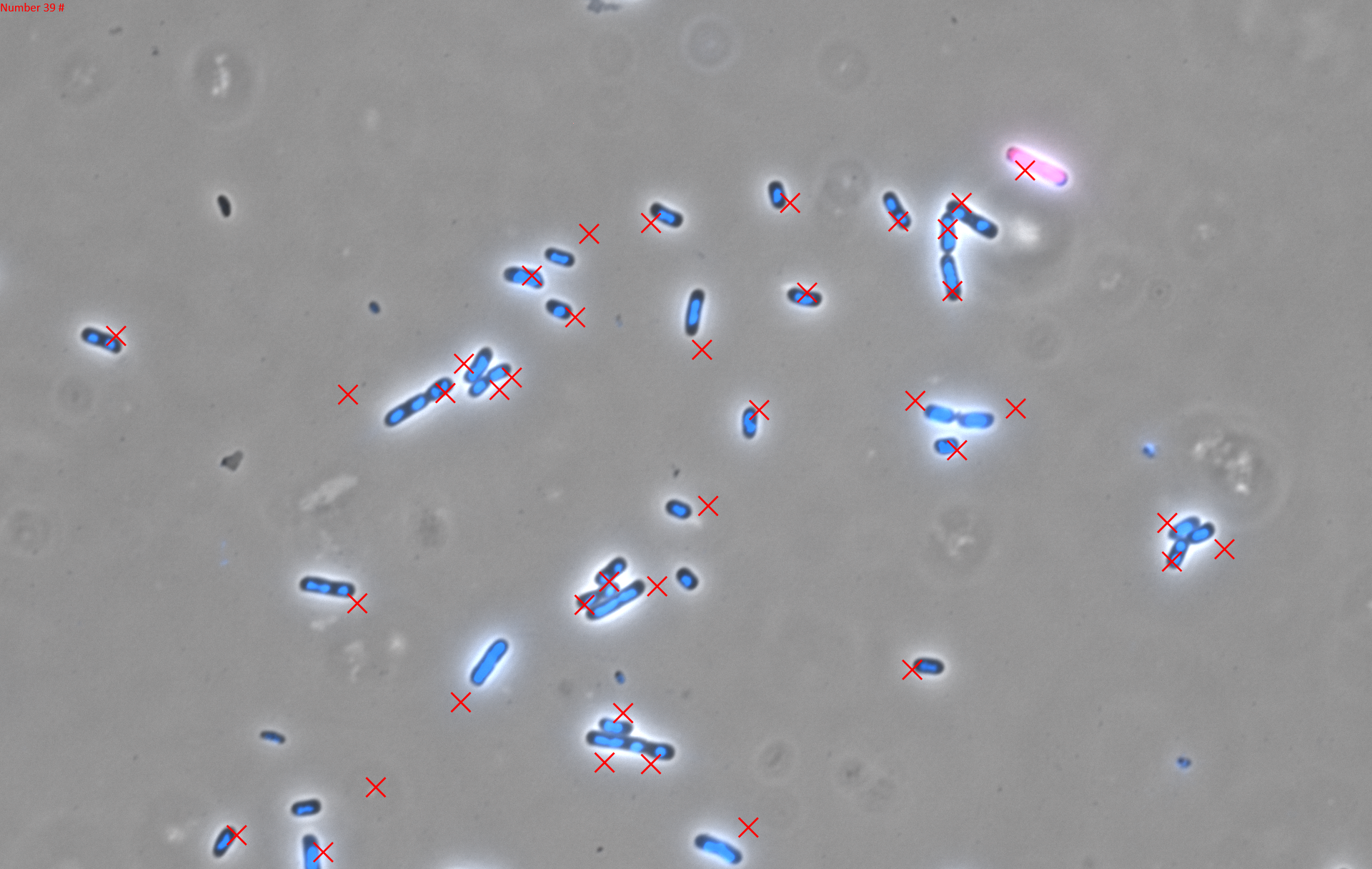

Supplement: Supplementary file 5 — Source Data for Figure 2 [file EMBR-24-e56849-s009.zip › 2C. Image and numerical data Micr.image+quantif/2C. Micr.image/Tde1(M)GLGL/Tde1(M)GLGL_4.tif]

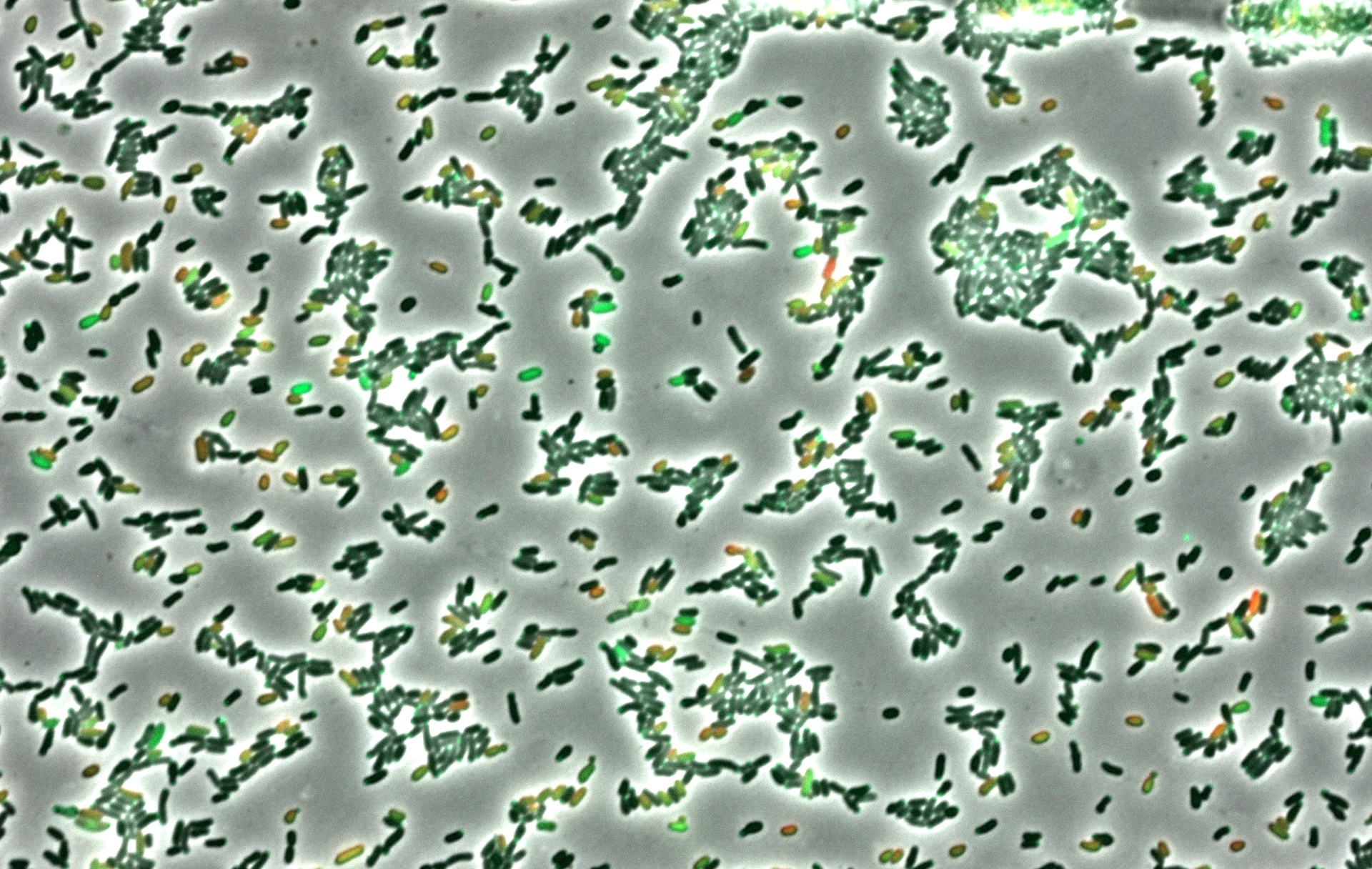

Supplement: Supplementary file 7 — Source Data for Figure 4 [file EMBR-24-e56849-s002.zip › 4A. Micr.image/Repeat 1/Tde1(M)/Tde1(M)_1.jpg]

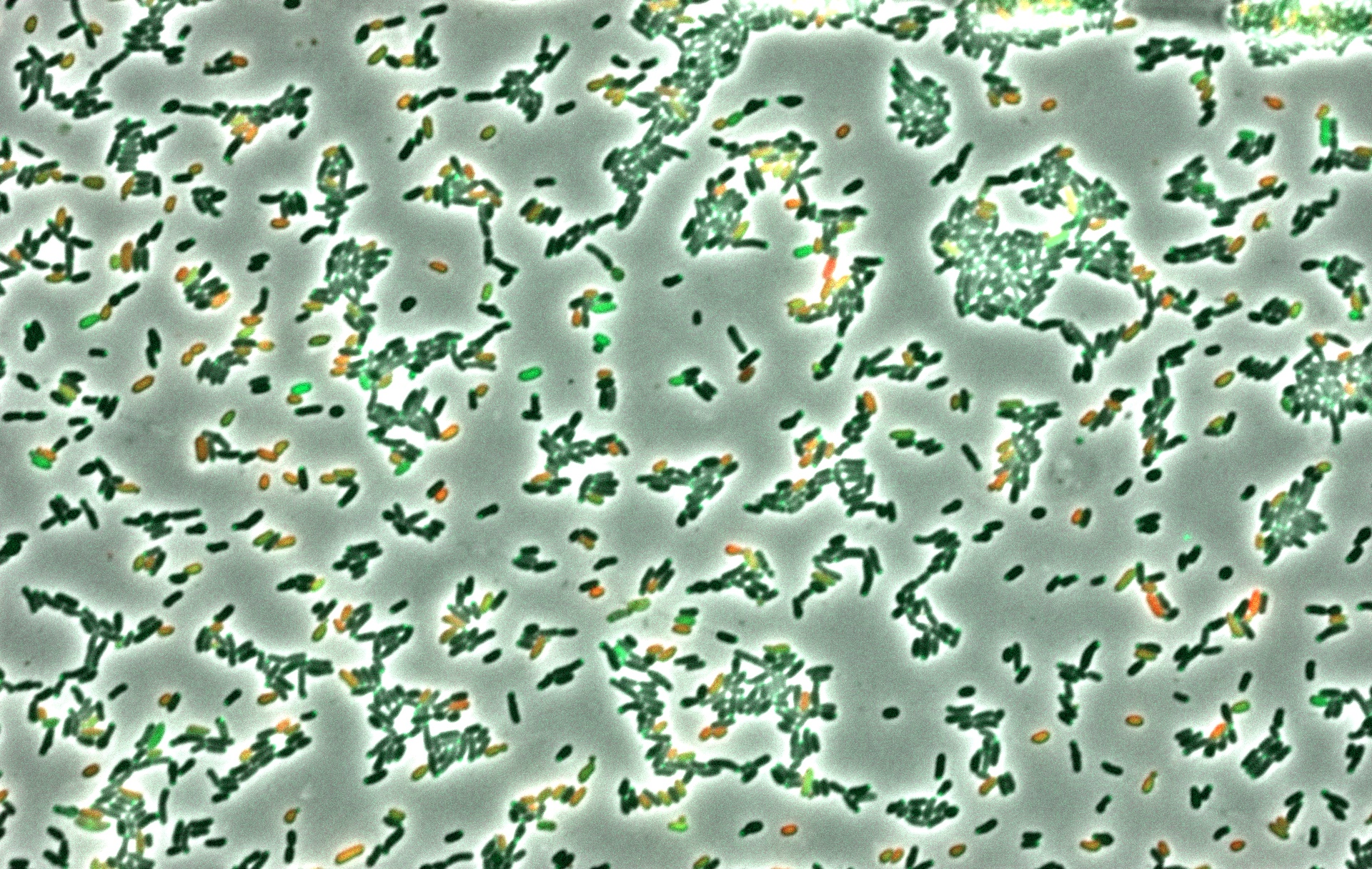

Supplement: Supplementary file 7 — Source Data for Figure 4 [file EMBR-24-e56849-s002.zip › 4A. Micr.image/Repeat 1/Tde1(M)/Tde1(M)_2.jpg]

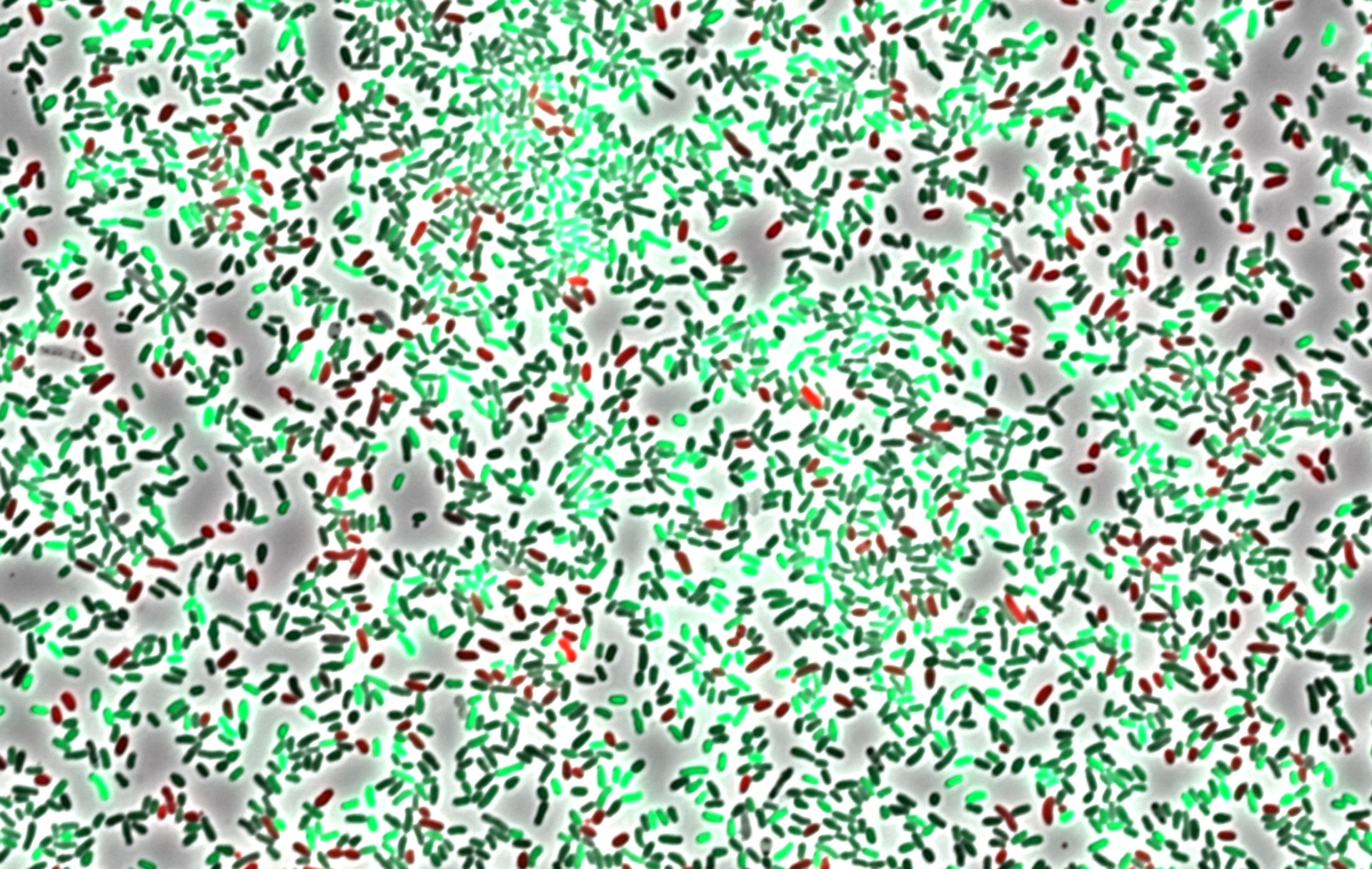

Supplement: Supplementary file 7 — Source Data for Figure 4 [file EMBR-24-e56849-s002.zip › 4A. Micr.image/Repeat 1/sfGFP/sfGFP_1.jpg]

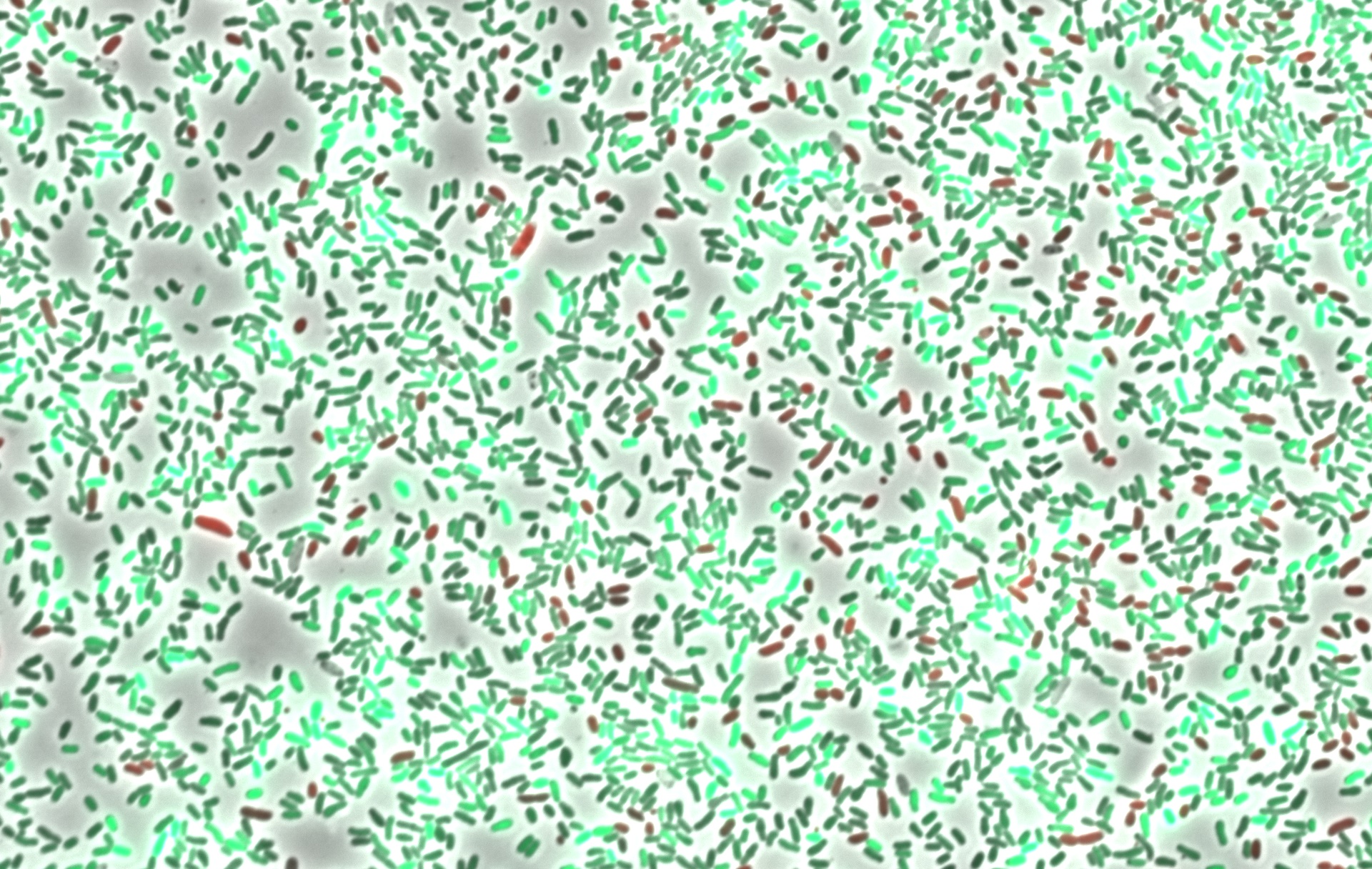

Supplement: Supplementary file 7 — Source Data for Figure 4 [file EMBR-24-e56849-s002.zip › 4A. Micr.image/Repeat 1/sfGFP/sfGFP_2.jpg]

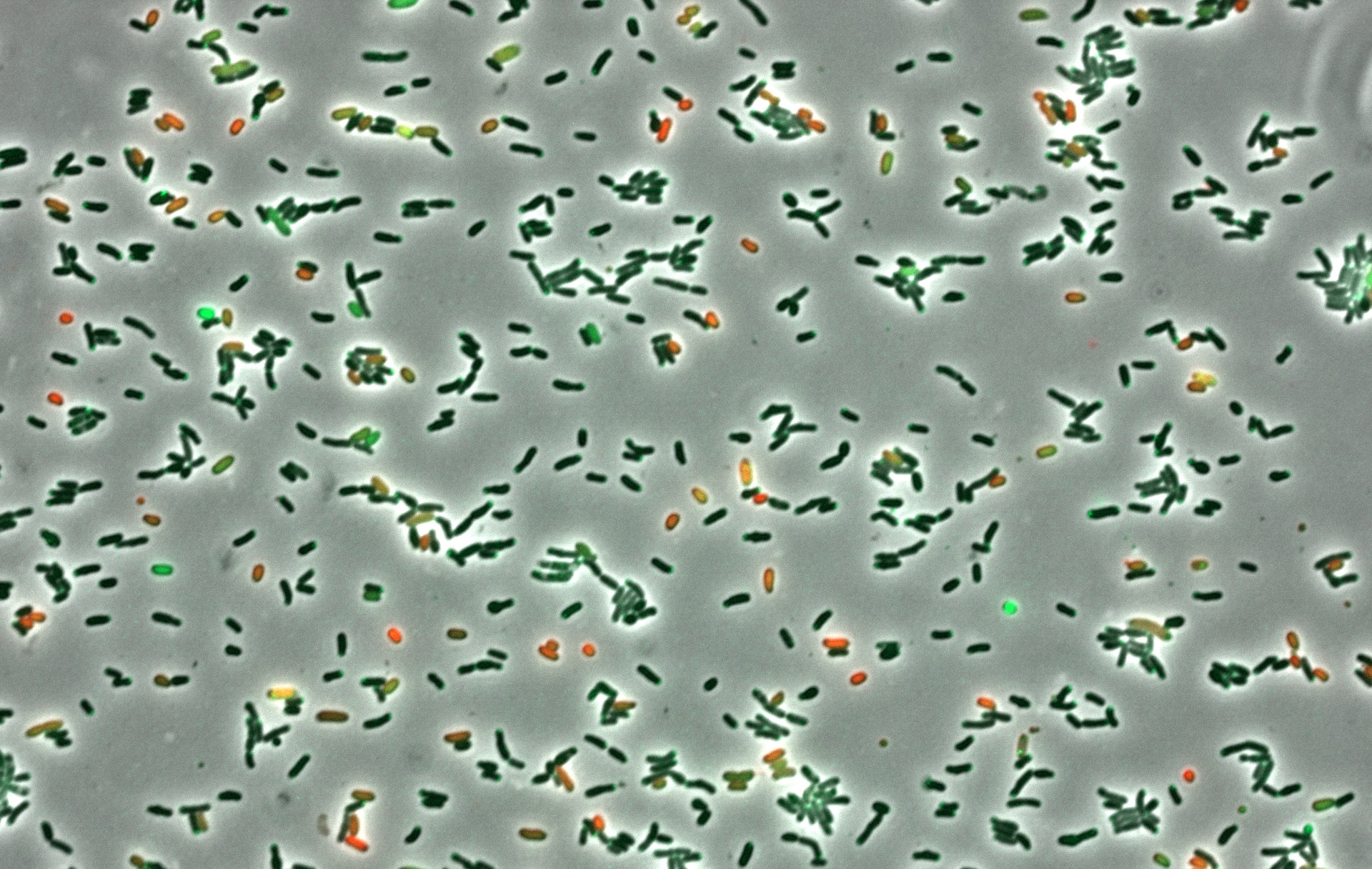

Supplement: Supplementary file 7 — Source Data for Figure 4 [file EMBR-24-e56849-s002.zip › 4A. Micr.image/Repeat 1/N-Tde1/N-Tde1_1.jpg]

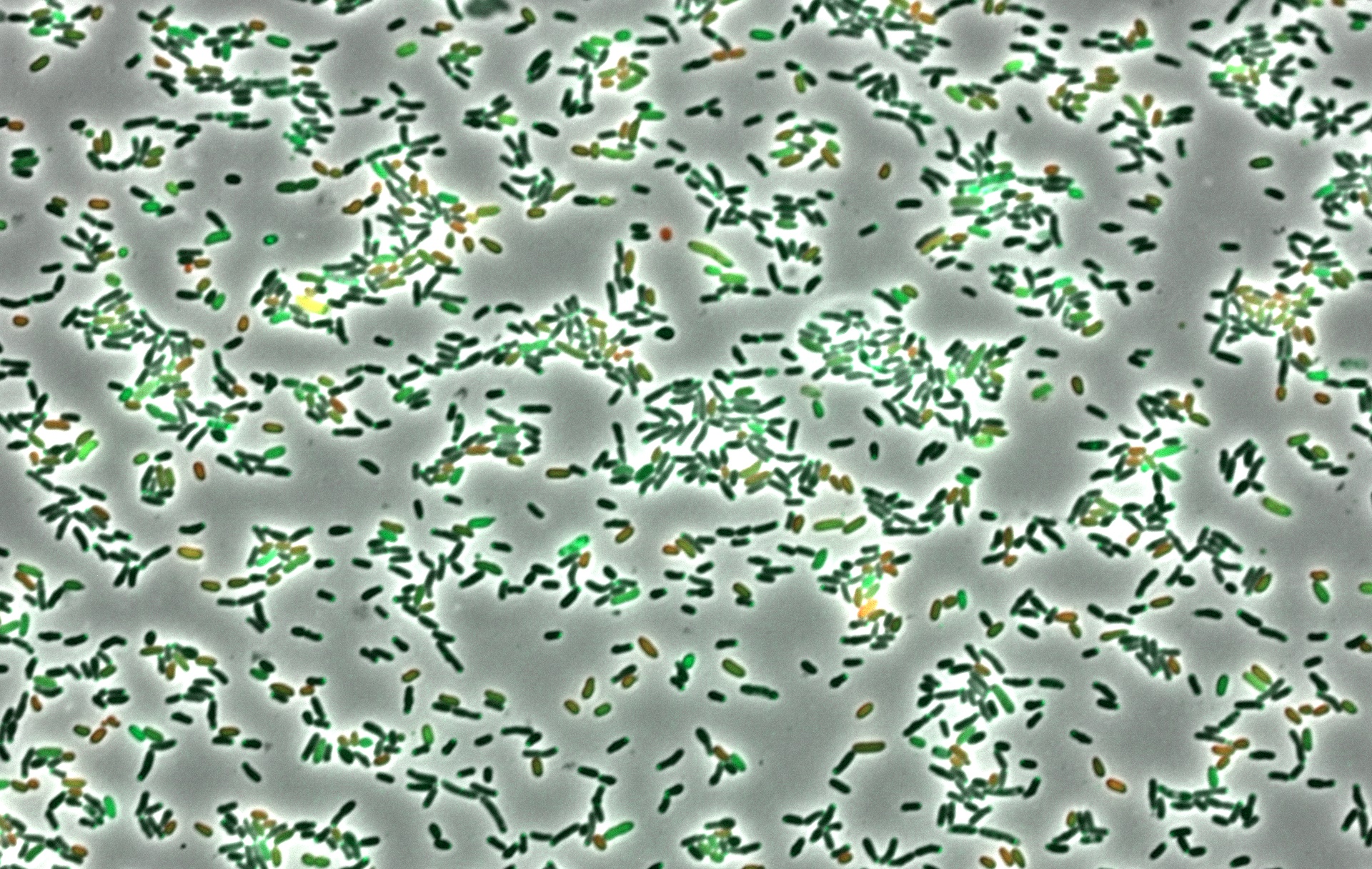

Supplement: Supplementary file 7 — Source Data for Figure 4 [file EMBR-24-e56849-s002.zip › 4A. Micr.image/Repeat 1/N-Tde1/N-tde1_2.jpg]

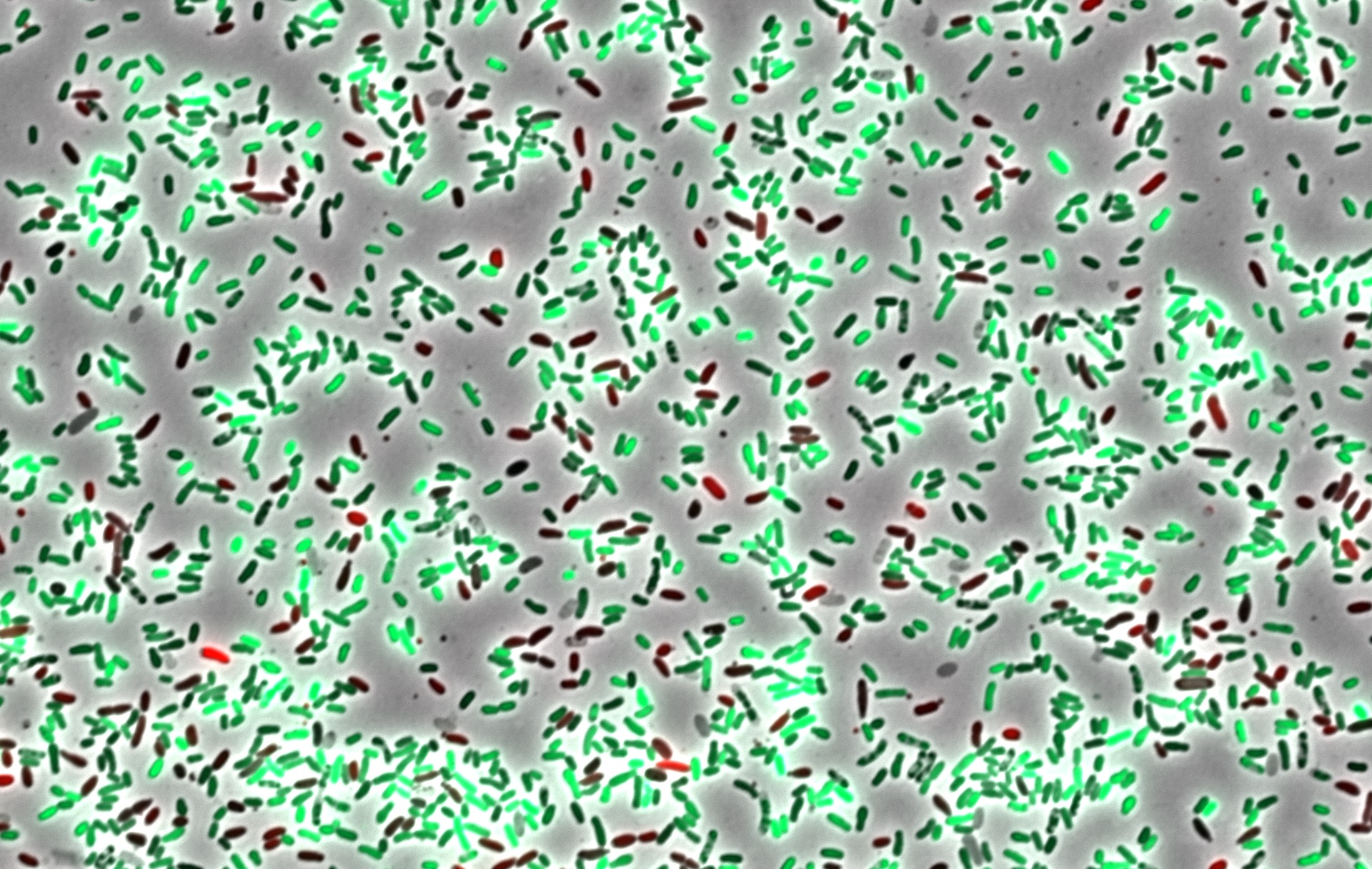

Supplement: Supplementary file 7 — Source Data for Figure 4 [file EMBR-24-e56849-s002.zip › 4A. Micr.image/Repeat 1/N-Tde1GLGL/N-Tde1GLGL_1.jpg]

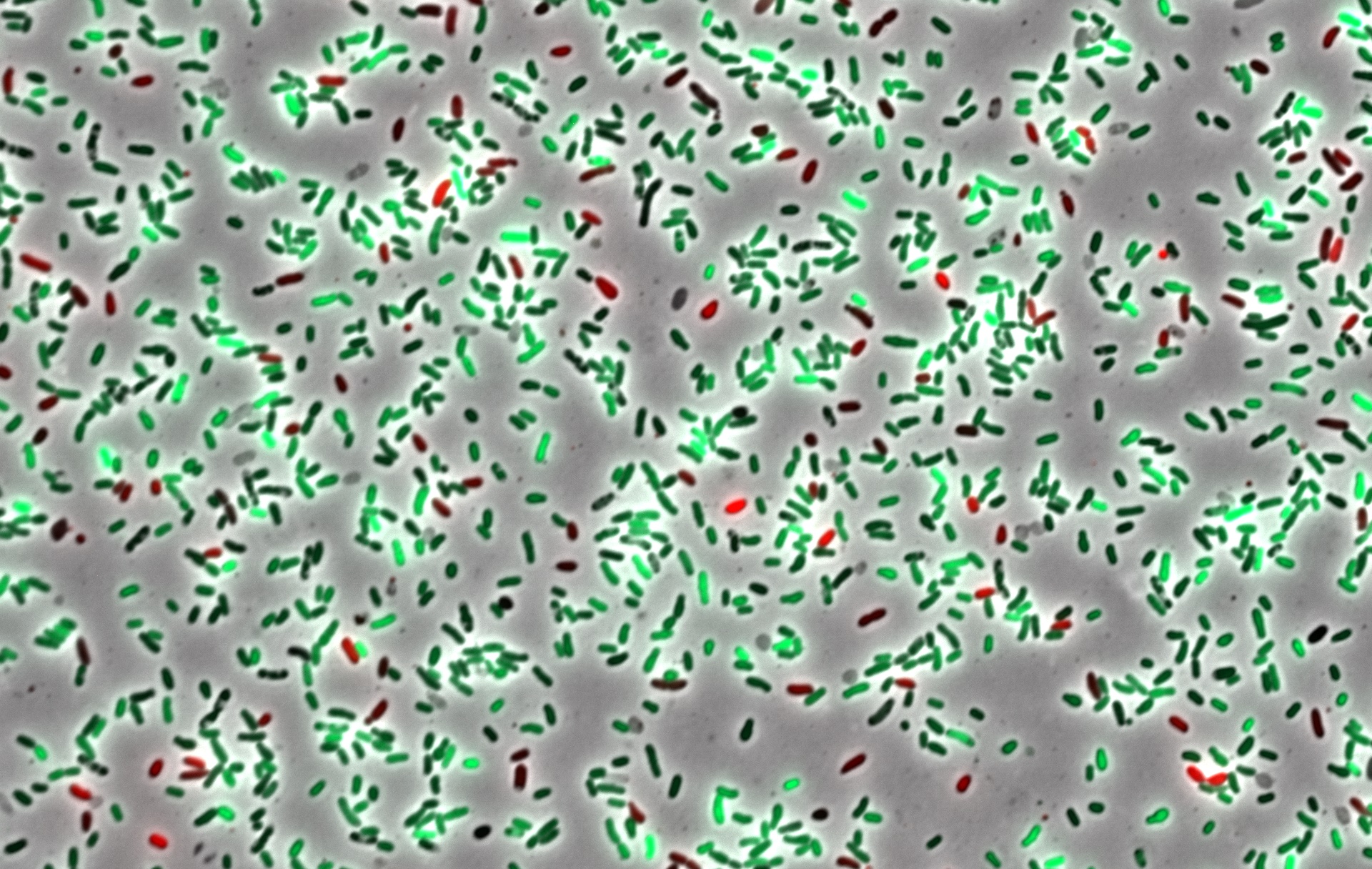

Supplement: Supplementary file 7 — Source Data for Figure 4 [file EMBR-24-e56849-s002.zip › 4A. Micr.image/Repeat 1/N-Tde1GLGL/N-Tde1GLGL_2.jpg]

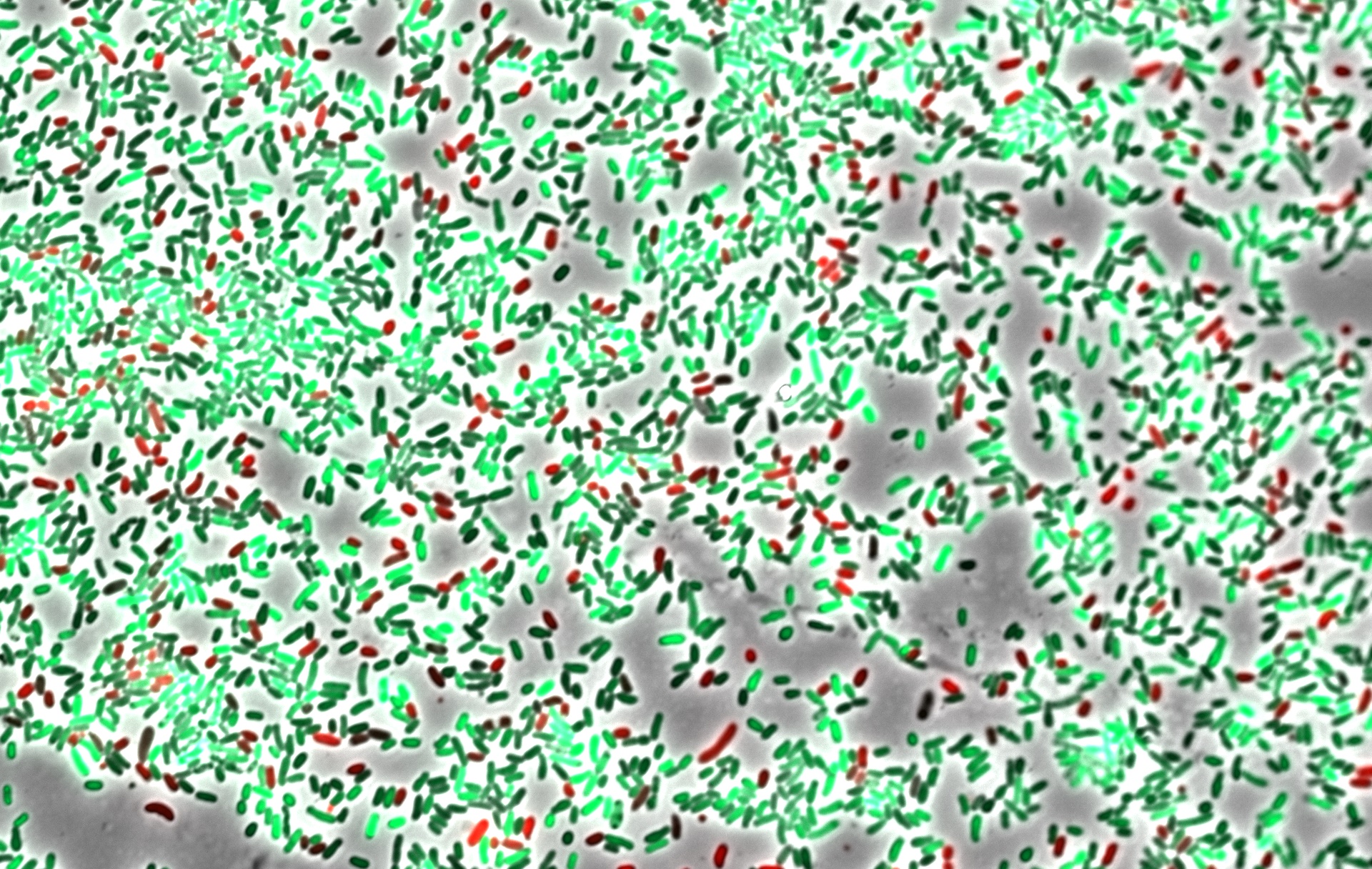

Supplement: Supplementary file 7 — Source Data for Figure 4 [file EMBR-24-e56849-s002.zip › 4A. Micr.image/Repeat 1/C1-Tde1/C1-Tde1_1.jpg]

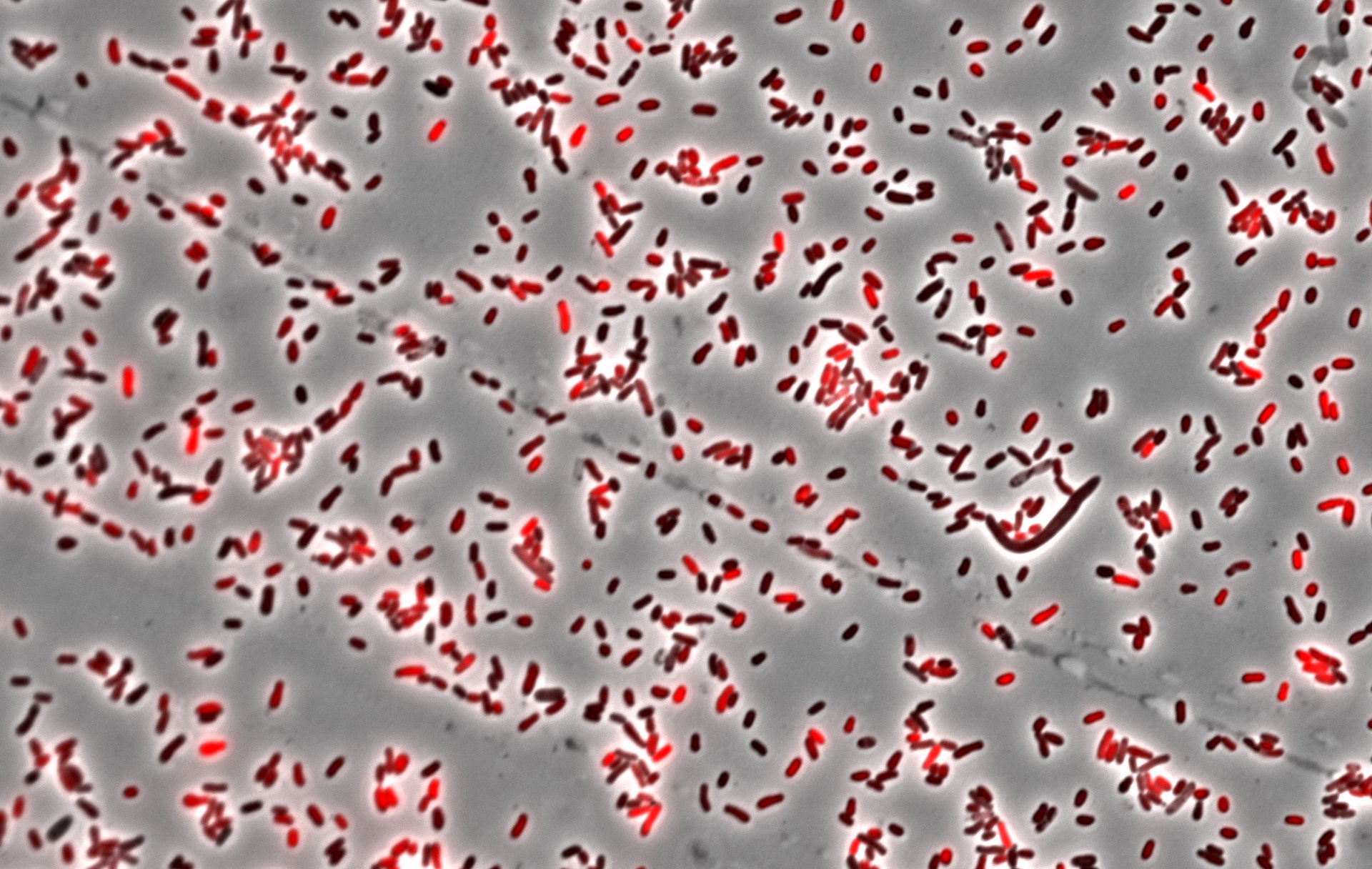

Supplement: Supplementary file 7 — Source Data for Figure 4 [file EMBR-24-e56849-s002.zip › 4A. Micr.image/Repeat 1/C1-Tde1/C1-Tde1_2.jpg]

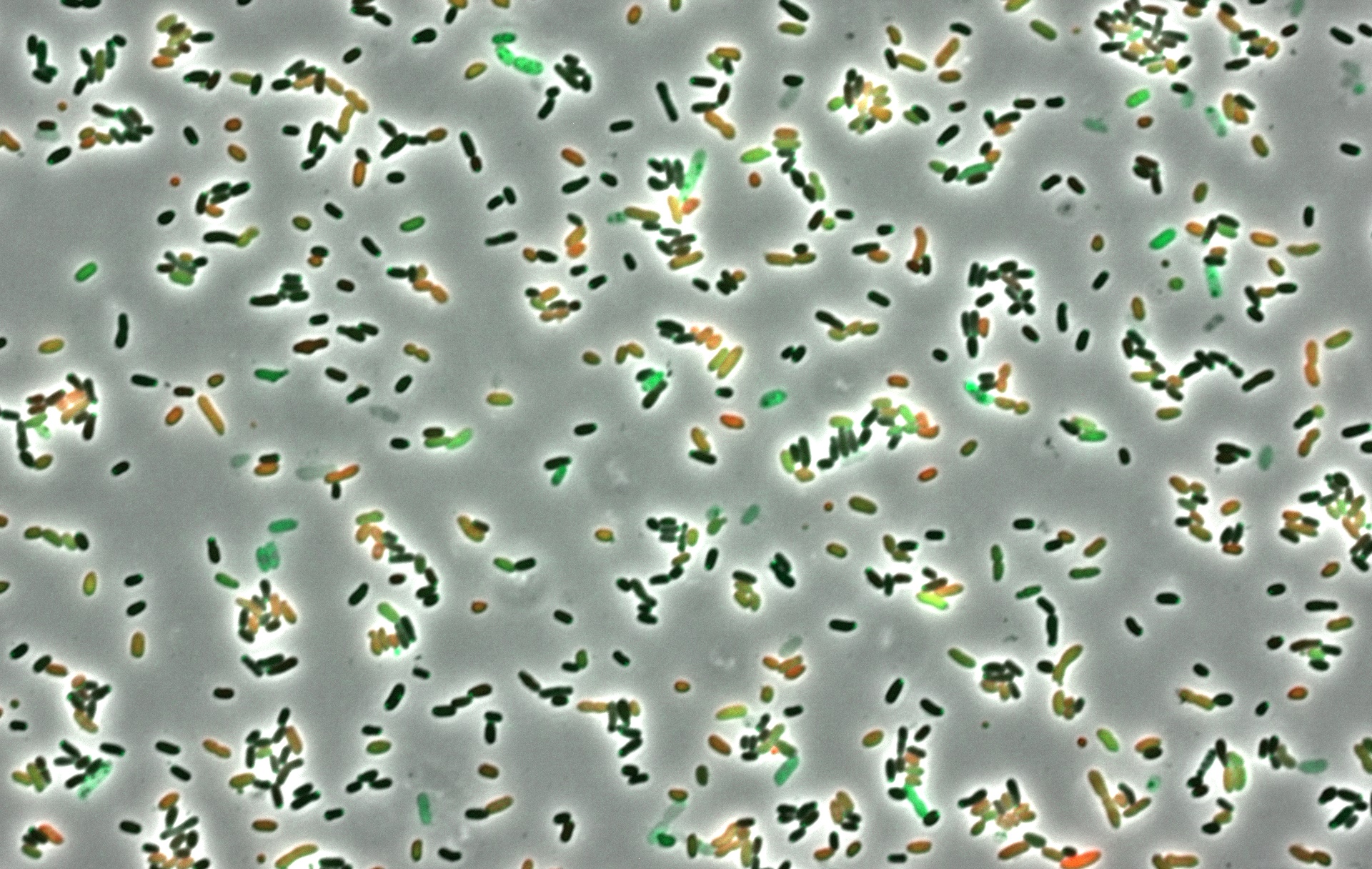

Supplement: Supplementary file 7 — Source Data for Figure 4 [file EMBR-24-e56849-s002.zip › 4A. Micr.image/Repeat 1/Tde1(M)GLGL/Tde1(M)GLGL_1.jpg]

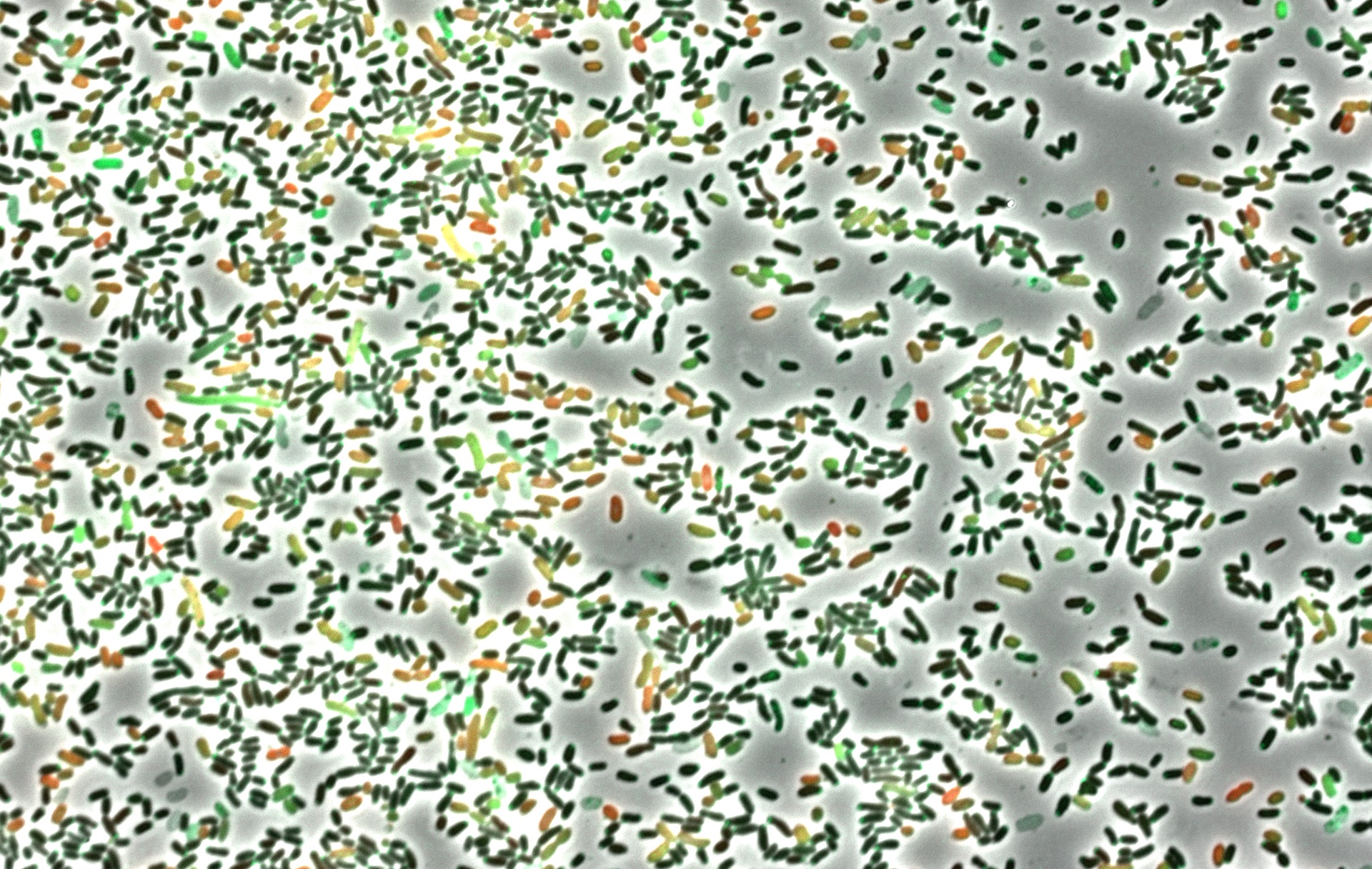

Supplement: Supplementary file 7 — Source Data for Figure 4 [file EMBR-24-e56849-s002.zip › 4A. Micr.image/Repeat 1/Tde1(M)GLGL/Tde1(M)GLGL_2.jpg]

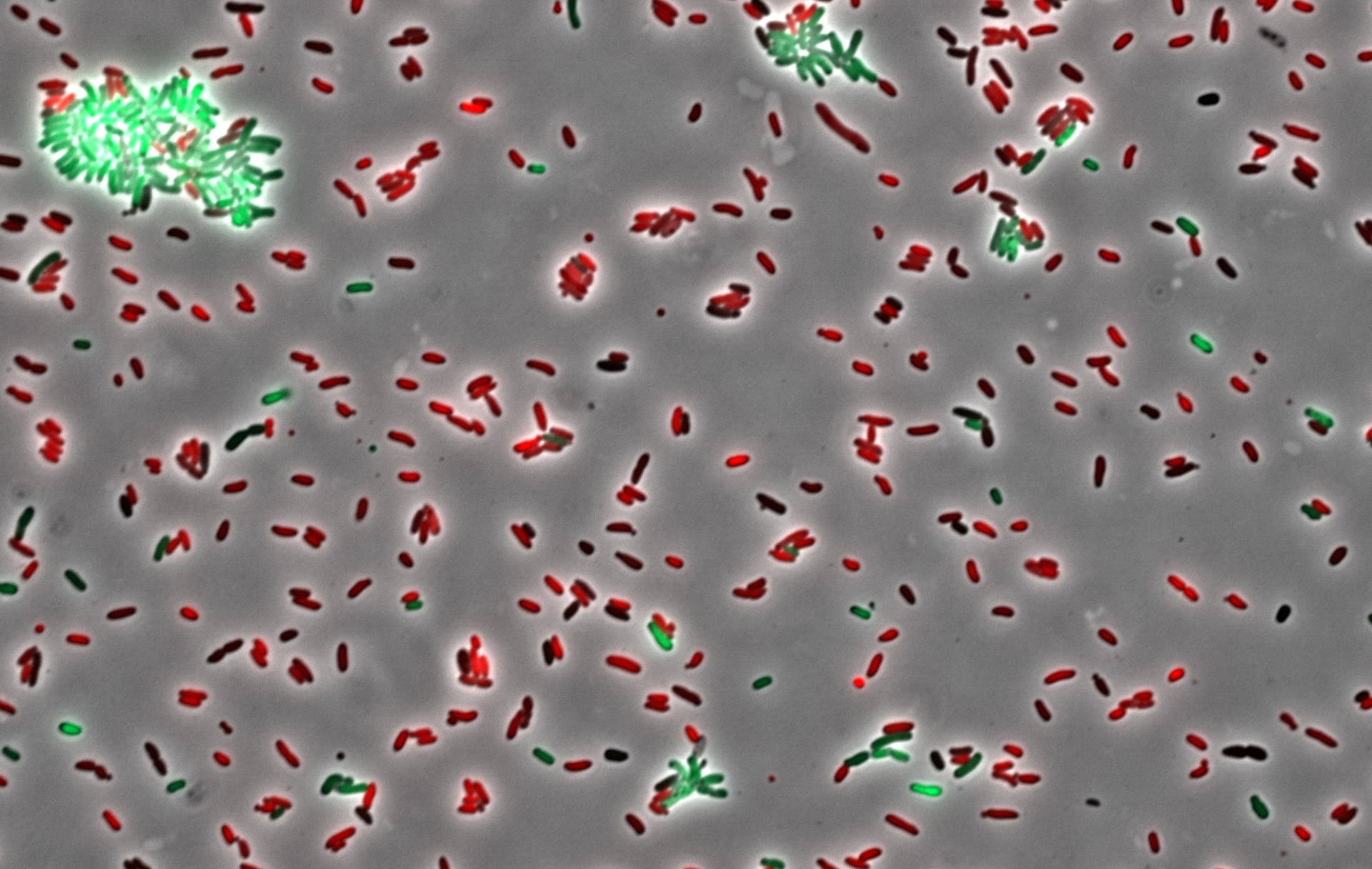

Supplement: Supplementary file 7 — Source Data for Figure 4 [file EMBR-24-e56849-s002.zip › 4A. Micr.image/repeat 3/dtdei dtssK Tde1(M)/Snap-1805_c1+2+3.jpg]

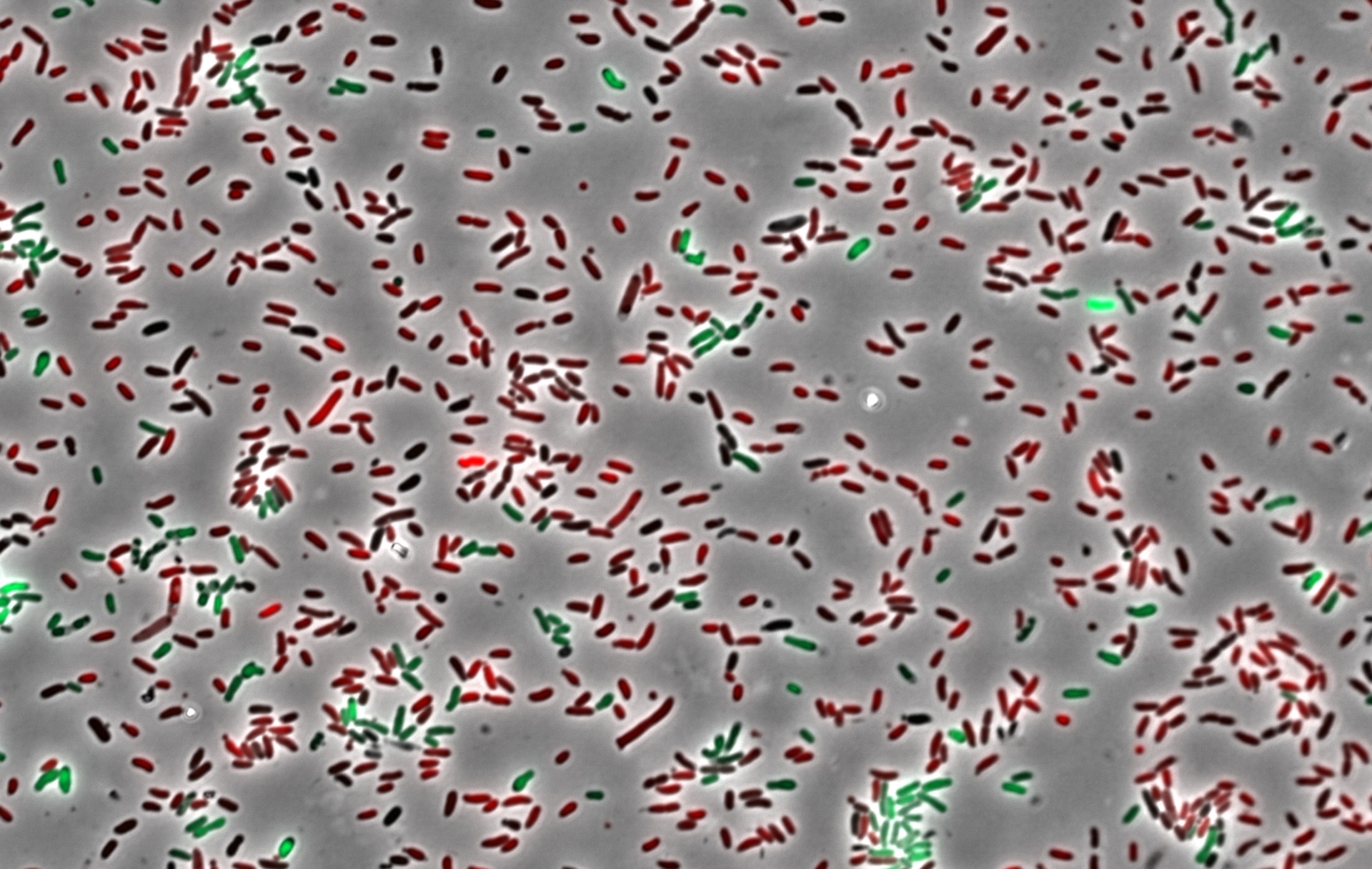

Supplement: Supplementary file 7 — Source Data for Figure 4 [file EMBR-24-e56849-s002.zip › 4A. Micr.image/repeat 3/dtdei dtssK Tde1(M)/Snap-1795_c1+2+3.jpg]

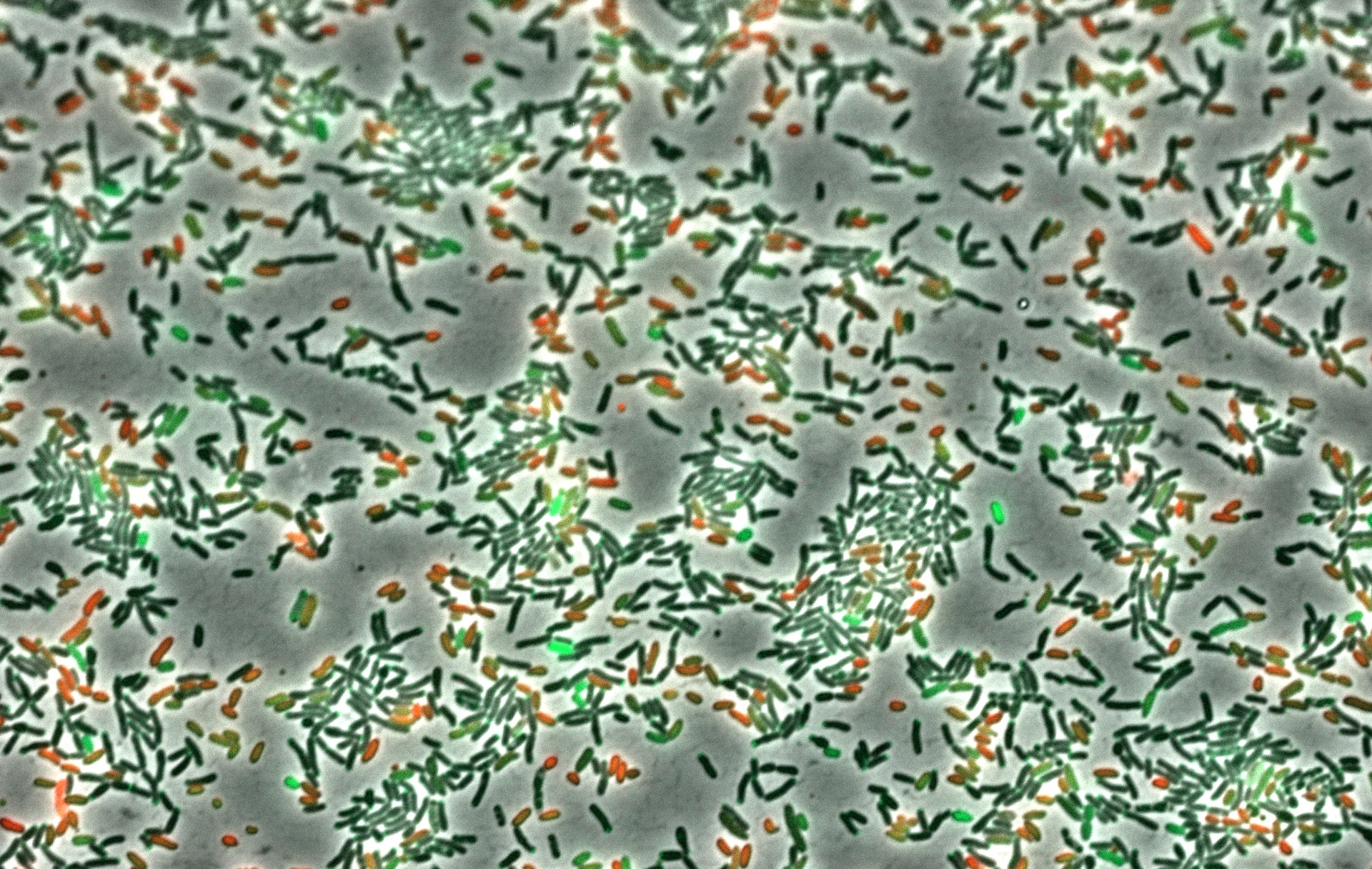

Supplement: Supplementary file 7 — Source Data for Figure 4 [file EMBR-24-e56849-s002.zip › 4A. Micr.image/repeat 3/Tde1(M)/Tde1(M)_1.jpg]

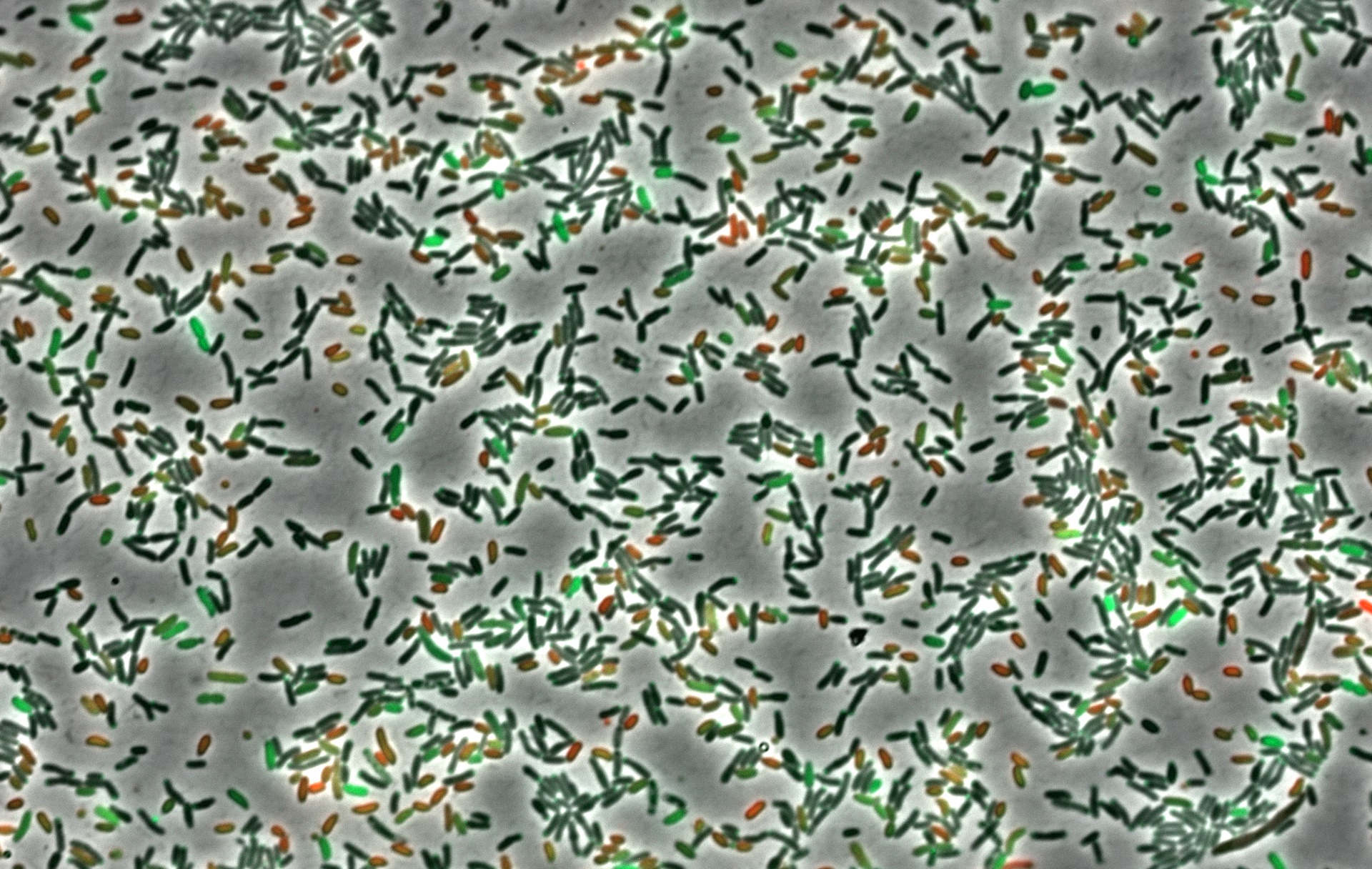

Supplement: Supplementary file 7 — Source Data for Figure 4 [file EMBR-24-e56849-s002.zip › 4A. Micr.image/repeat 3/Tde1(M)/Tde1(M)_2.jpg]

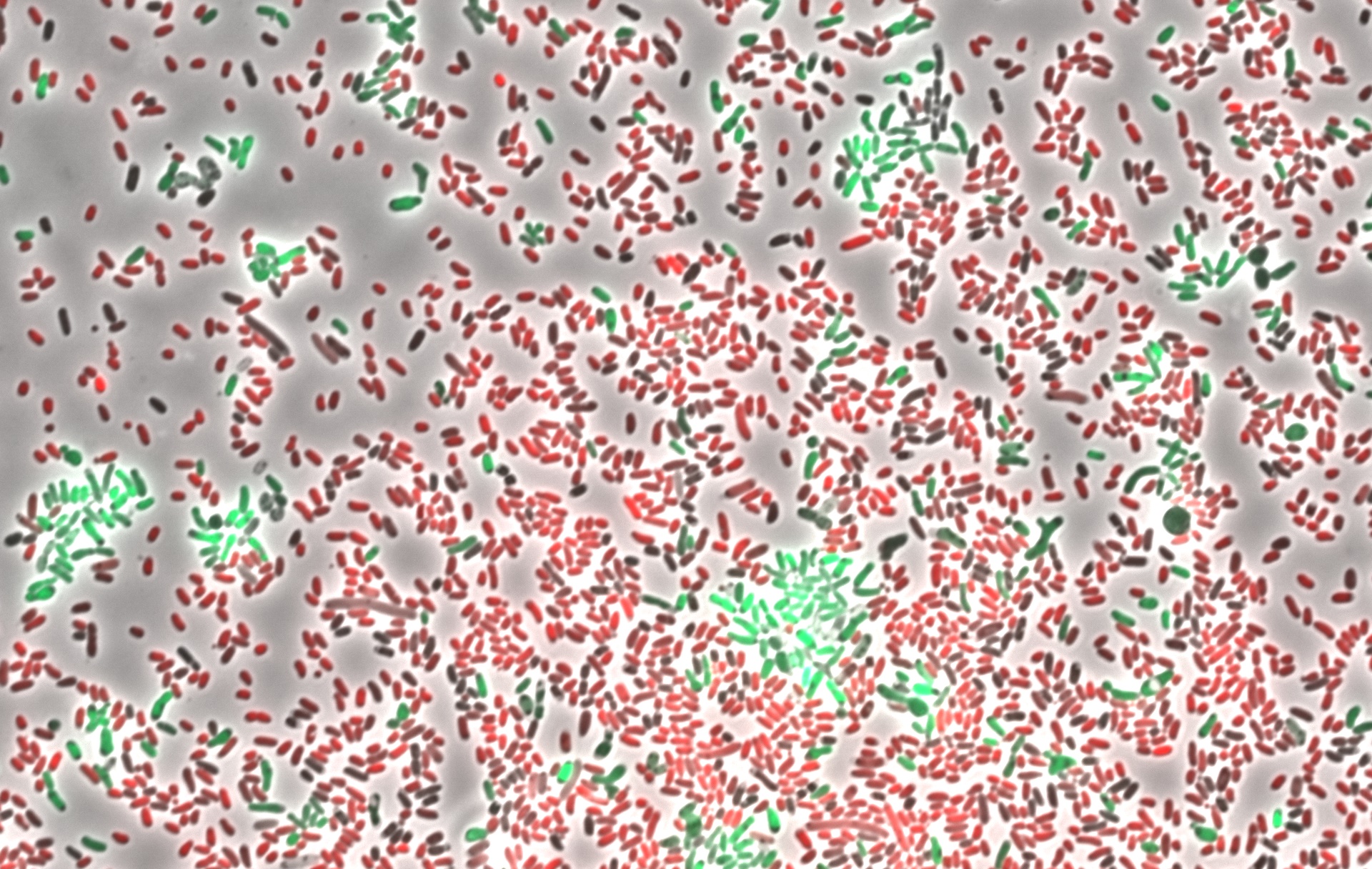

Supplement: Supplementary file 7 — Source Data for Figure 4 [file EMBR-24-e56849-s002.zip › 4A. Micr.image/repeat 3/dtdei dtssk N-Tde1/dtdei dtssk N-Tde1_2.jpg]

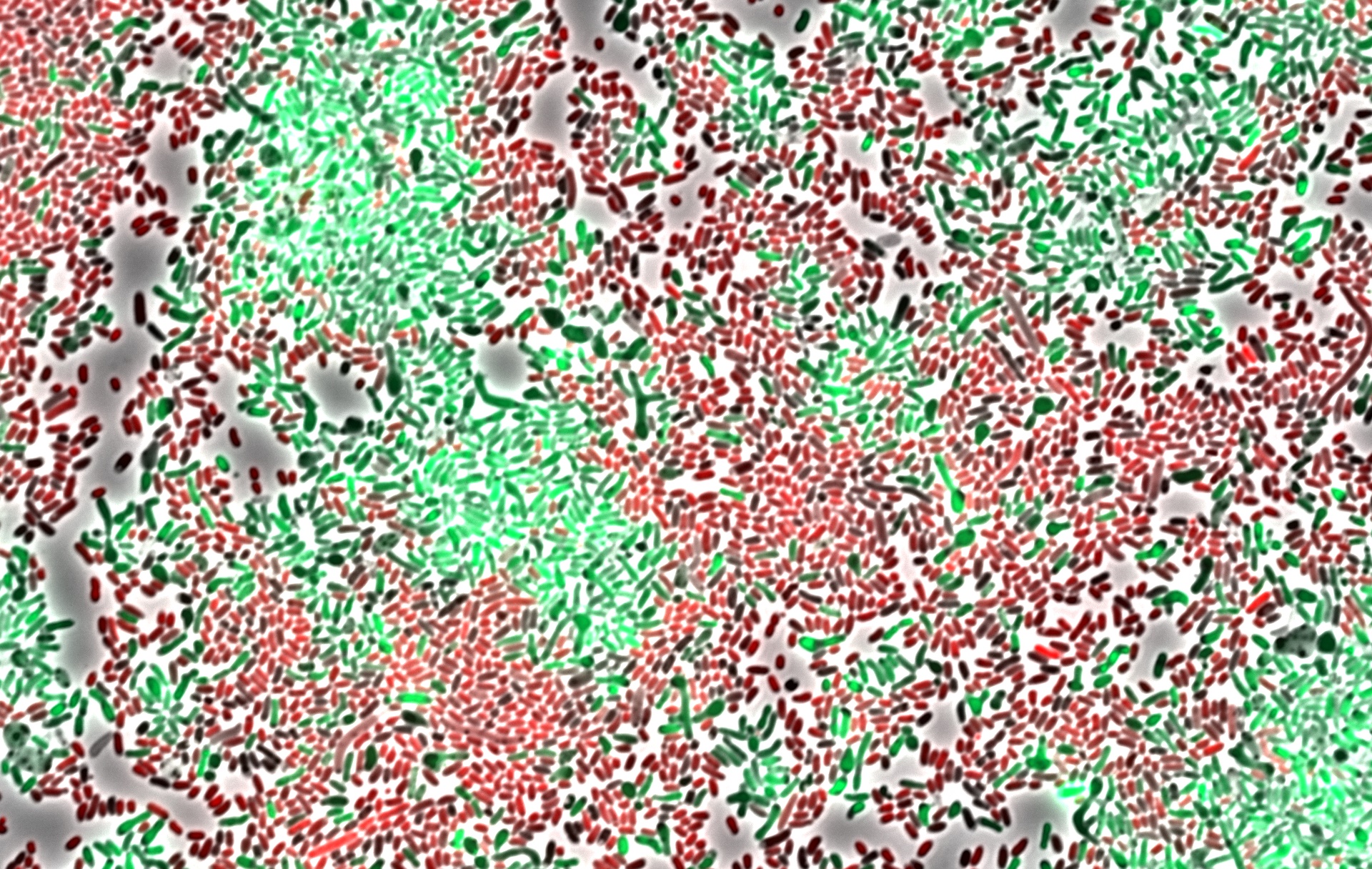

Supplement: Supplementary file 7 — Source Data for Figure 4 [file EMBR-24-e56849-s002.zip › 4A. Micr.image/repeat 3/dtdei dtssk N-Tde1/dtdei dtssk N-Tde1_1.jpg]

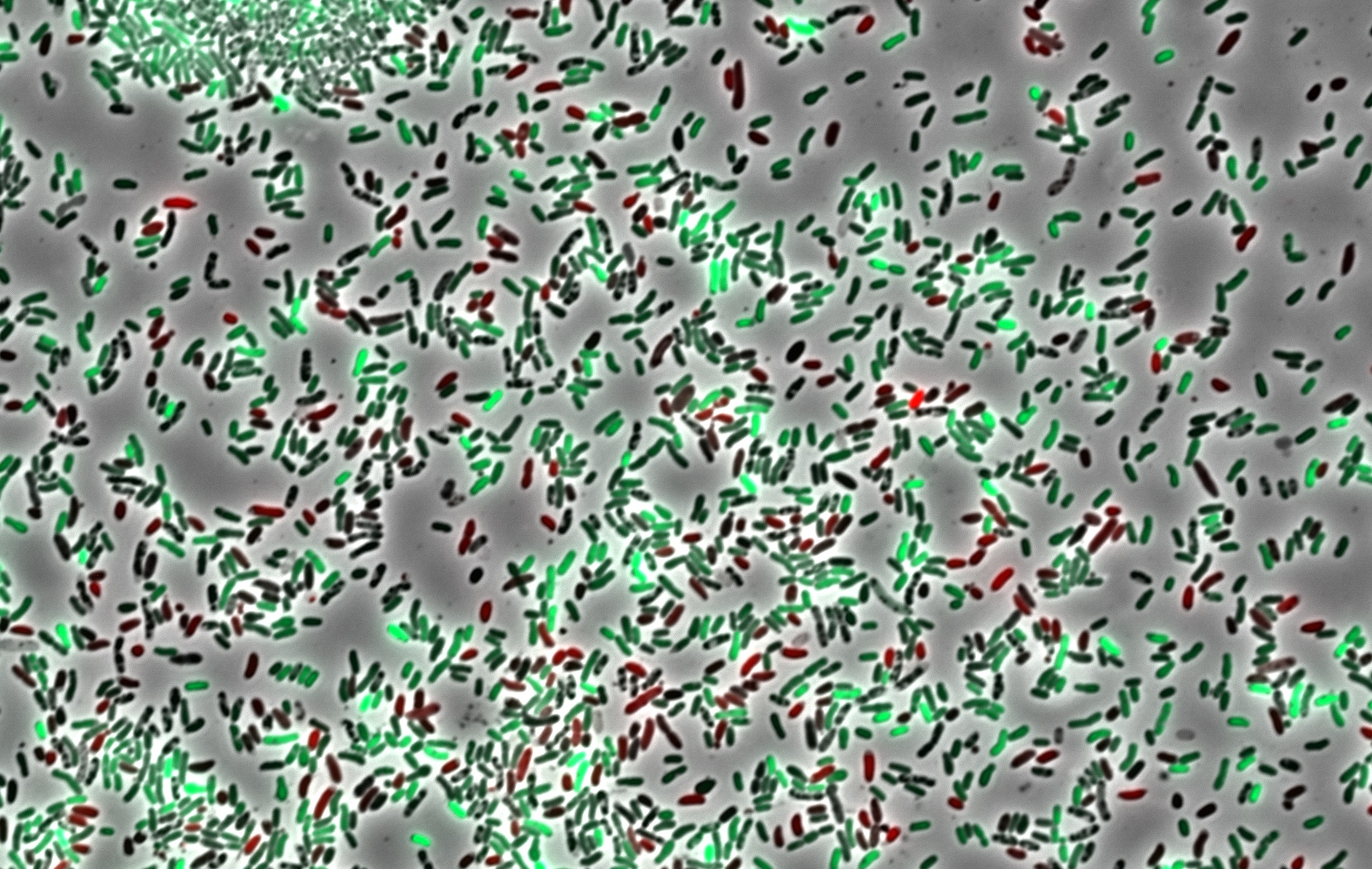

Supplement: Supplementary file 7 — Source Data for Figure 4 [file EMBR-24-e56849-s002.zip › 4A. Micr.image/repeat 3/sfGFP/sfGFP_1.jpg]

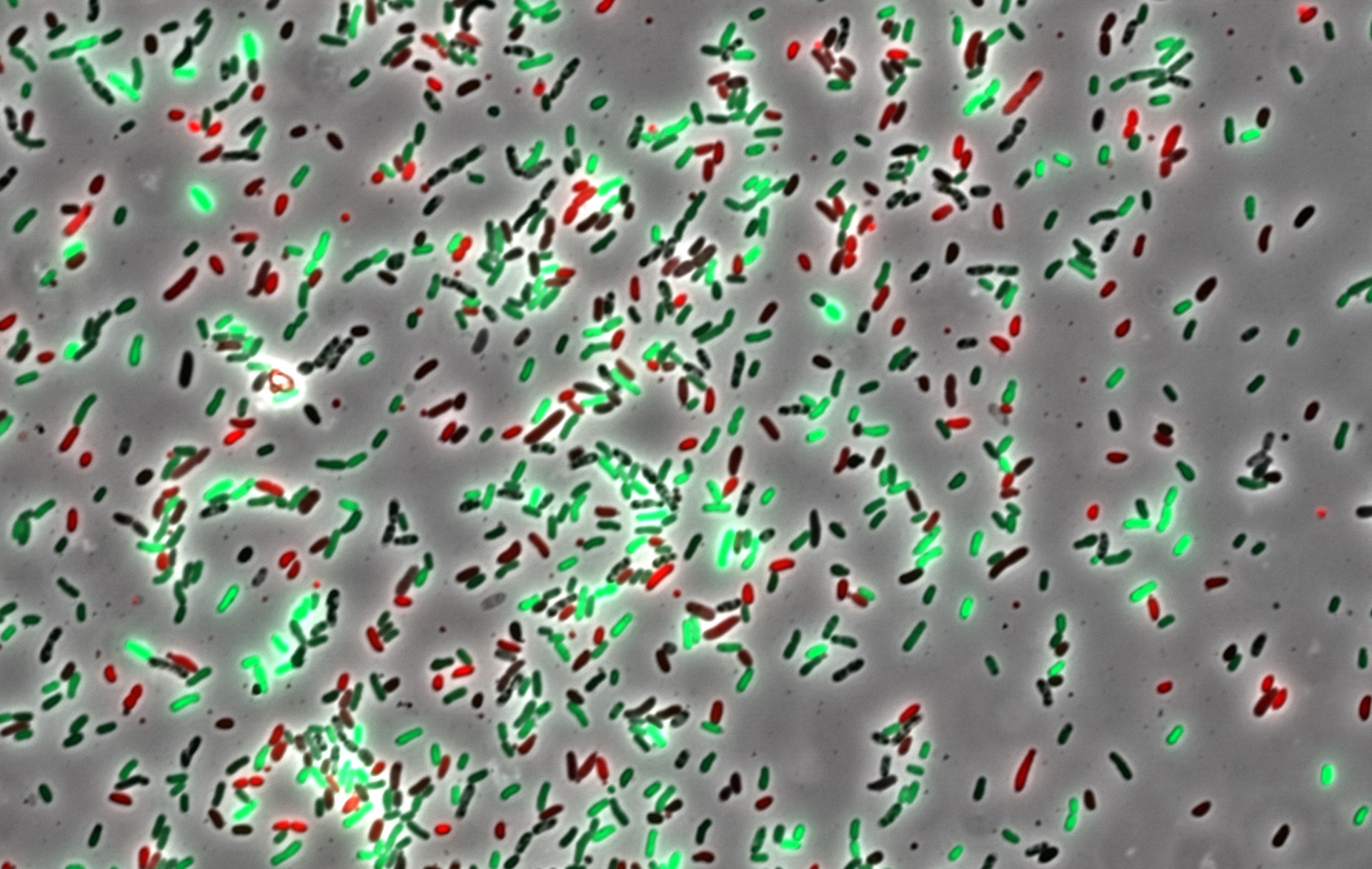

Supplement: Supplementary file 7 — Source Data for Figure 4 [file EMBR-24-e56849-s002.zip › 4A. Micr.image/repeat 3/sfGFP/sfGFP_2.jpg]

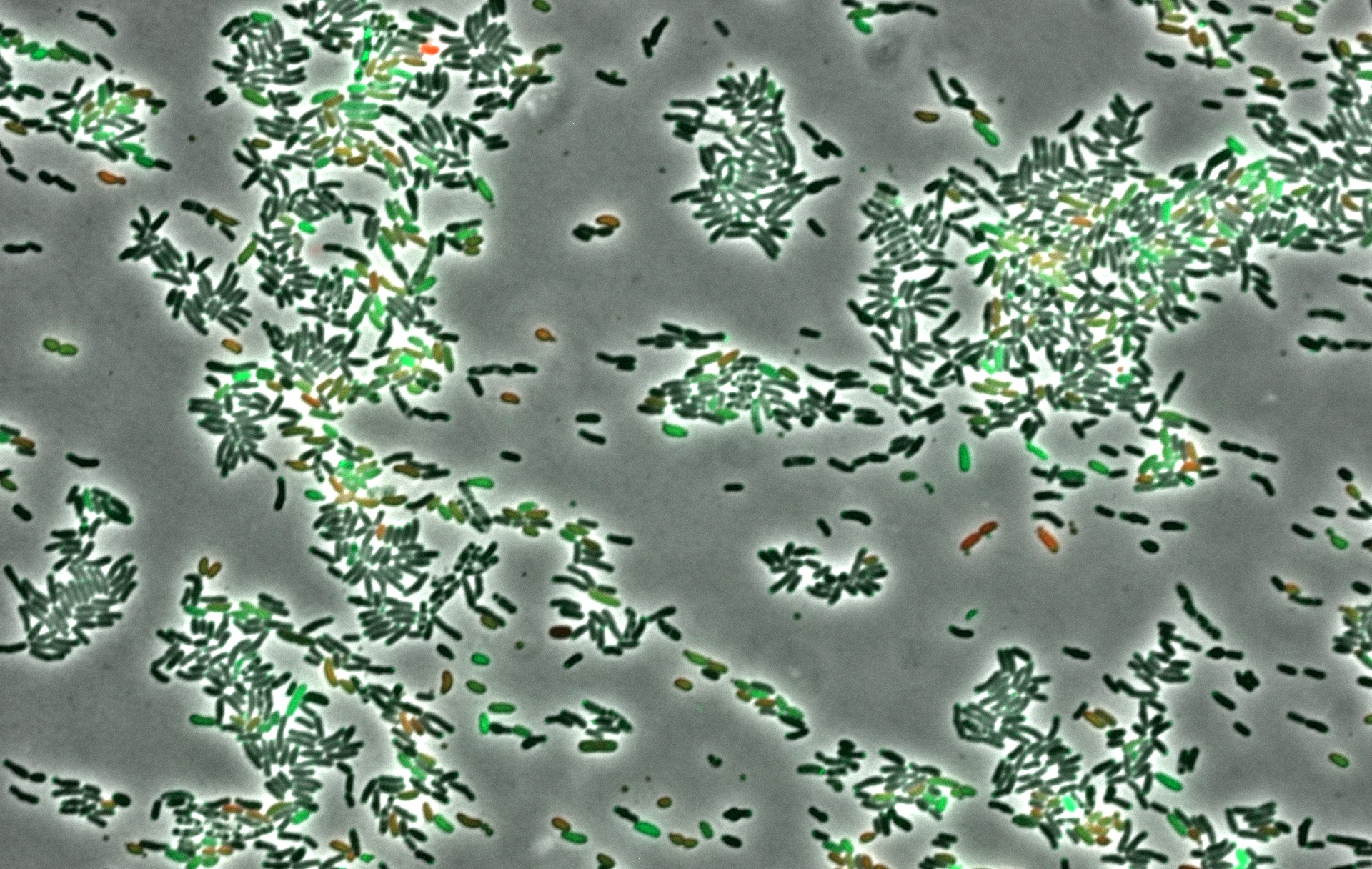

Supplement: Supplementary file 7 — Source Data for Figure 4 [file EMBR-24-e56849-s002.zip › 4A. Micr.image/repeat 3/N-Tde1/N-Tde1_1.jpg]

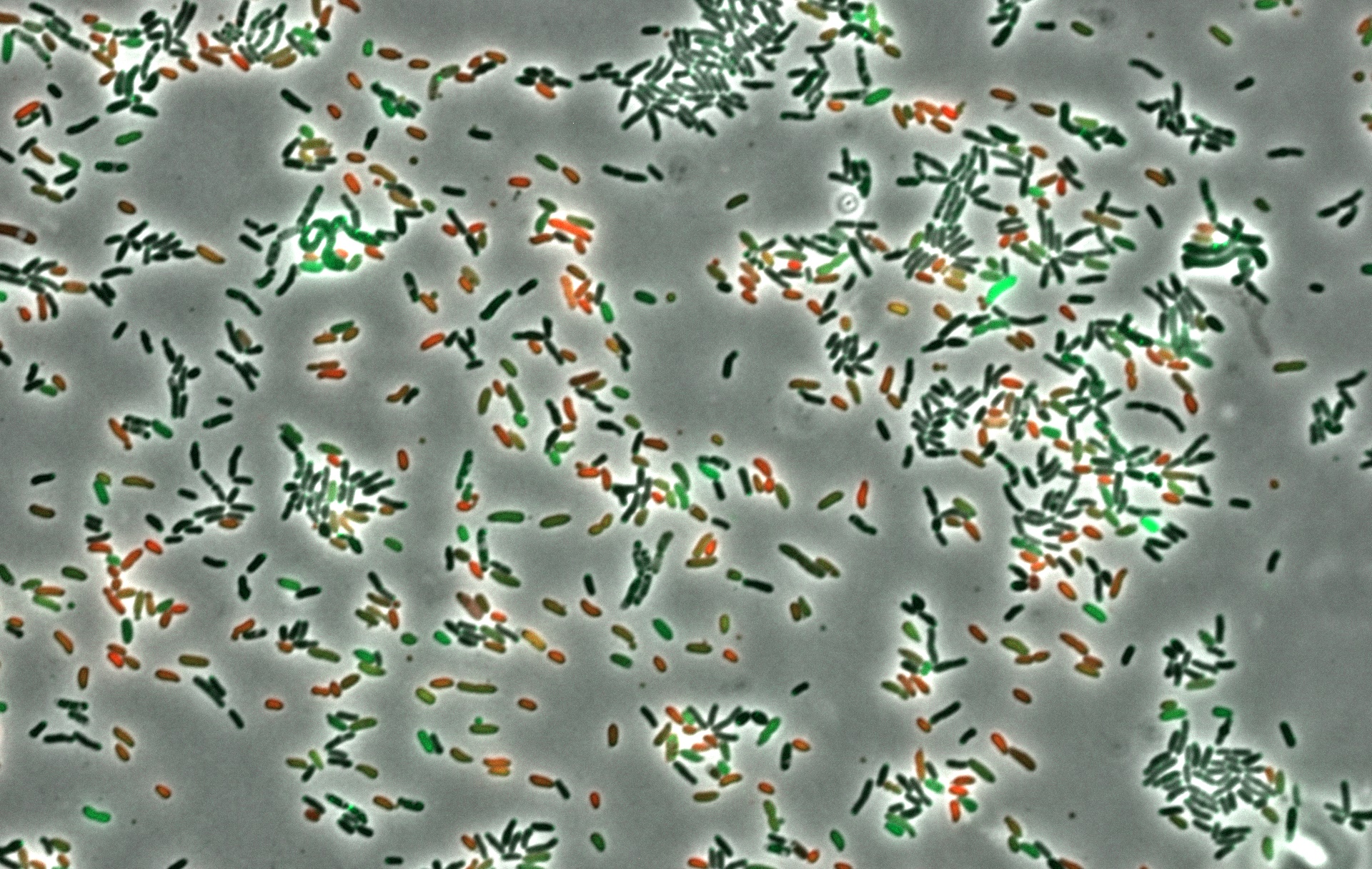

Supplement: Supplementary file 7 — Source Data for Figure 4 [file EMBR-24-e56849-s002.zip › 4A. Micr.image/repeat 3/N-Tde1/N-Tde1_2.jpg]

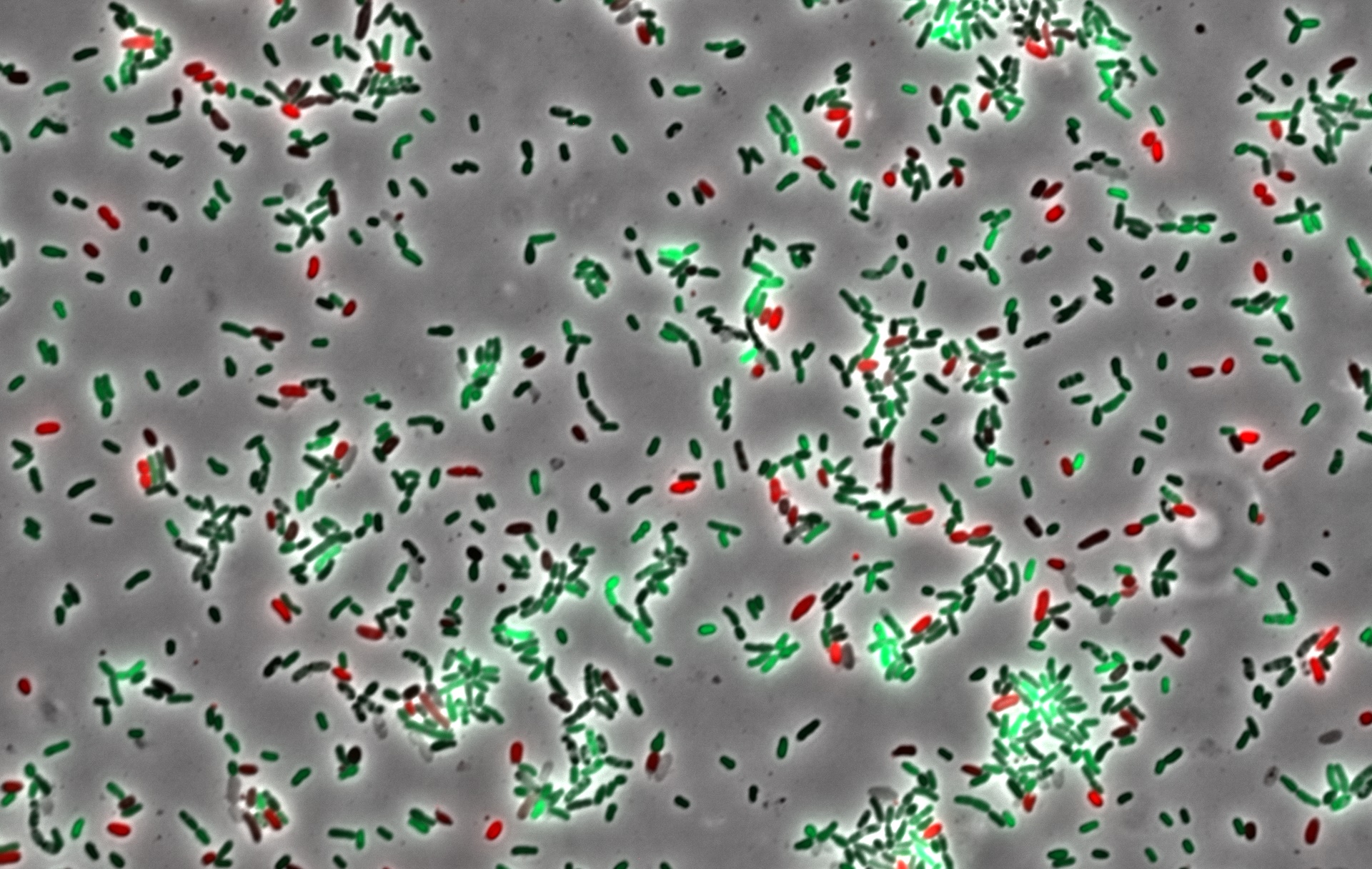

Supplement: Supplementary file 7 — Source Data for Figure 4 [file EMBR-24-e56849-s002.zip › 4A. Micr.image/repeat 3/N-Tde1GLGL/N-Tde1GLGL_1.jpg]

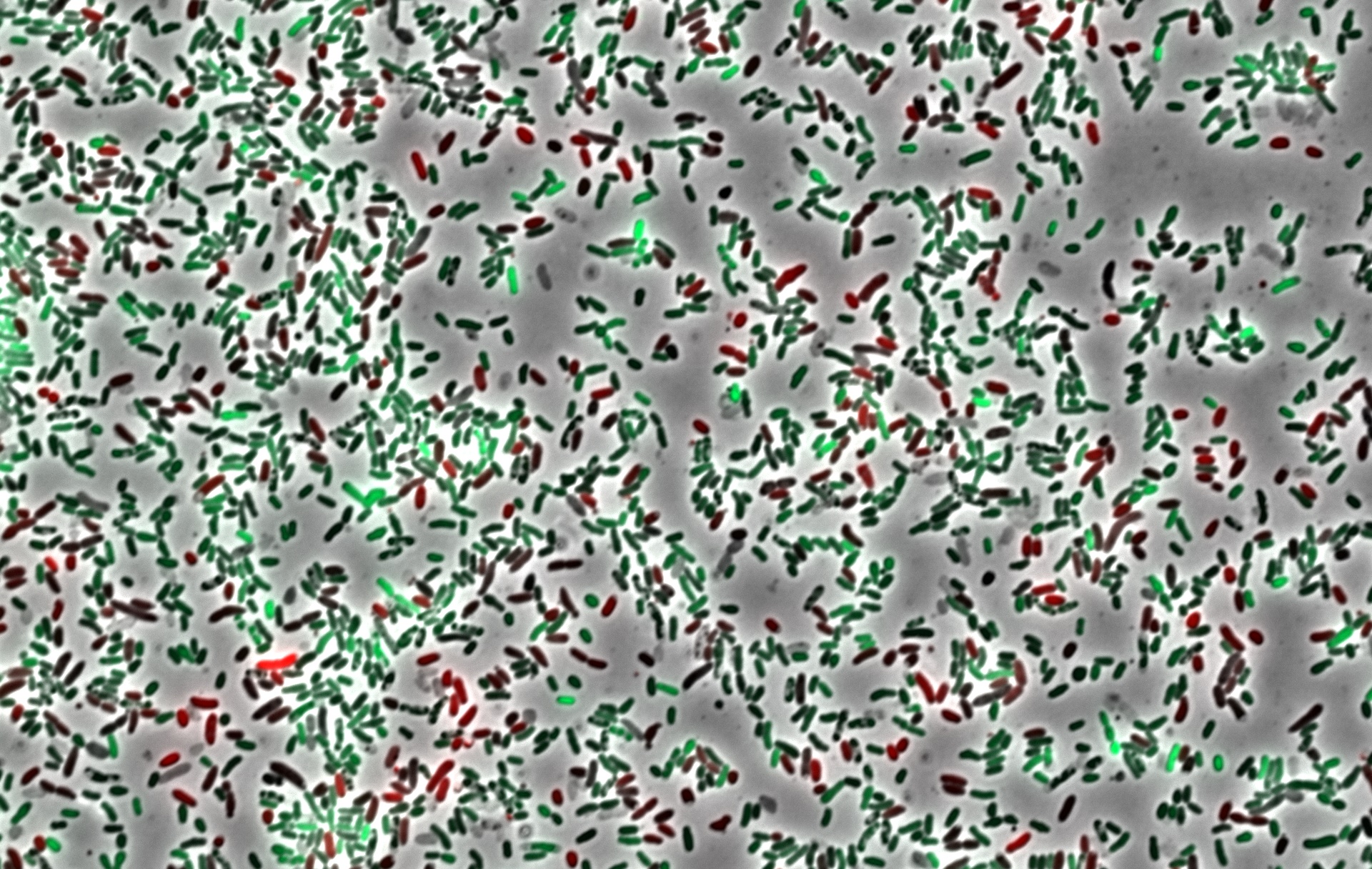

Supplement: Supplementary file 7 — Source Data for Figure 4 [file EMBR-24-e56849-s002.zip › 4A. Micr.image/repeat 3/N-Tde1GLGL/N-Tde1GLGL_2.jpg]

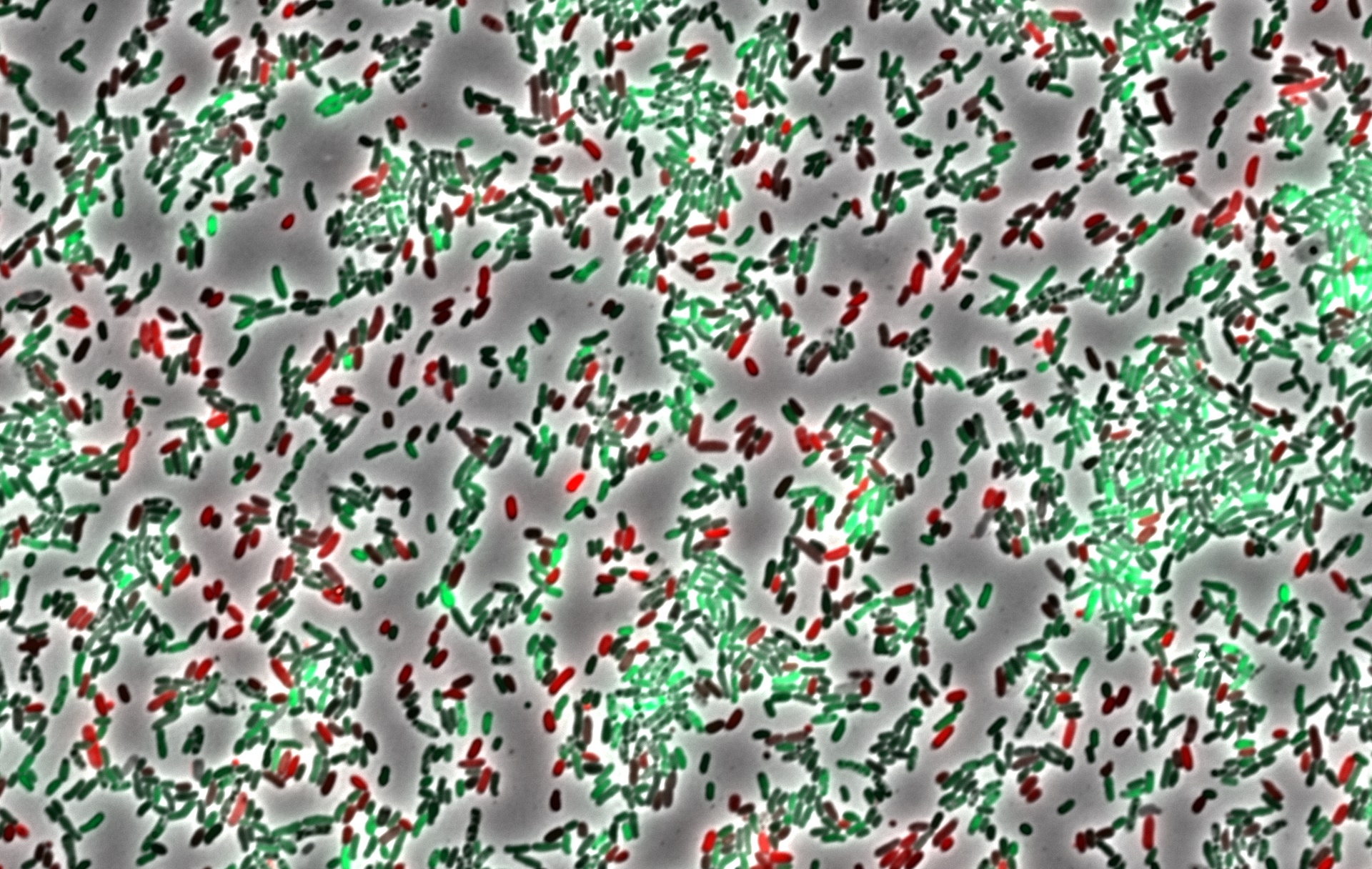

Supplement: Supplementary file 7 — Source Data for Figure 4 [file EMBR-24-e56849-s002.zip › 4A. Micr.image/repeat 3/C1-Tde1/C1-Tde1_1.jpg]

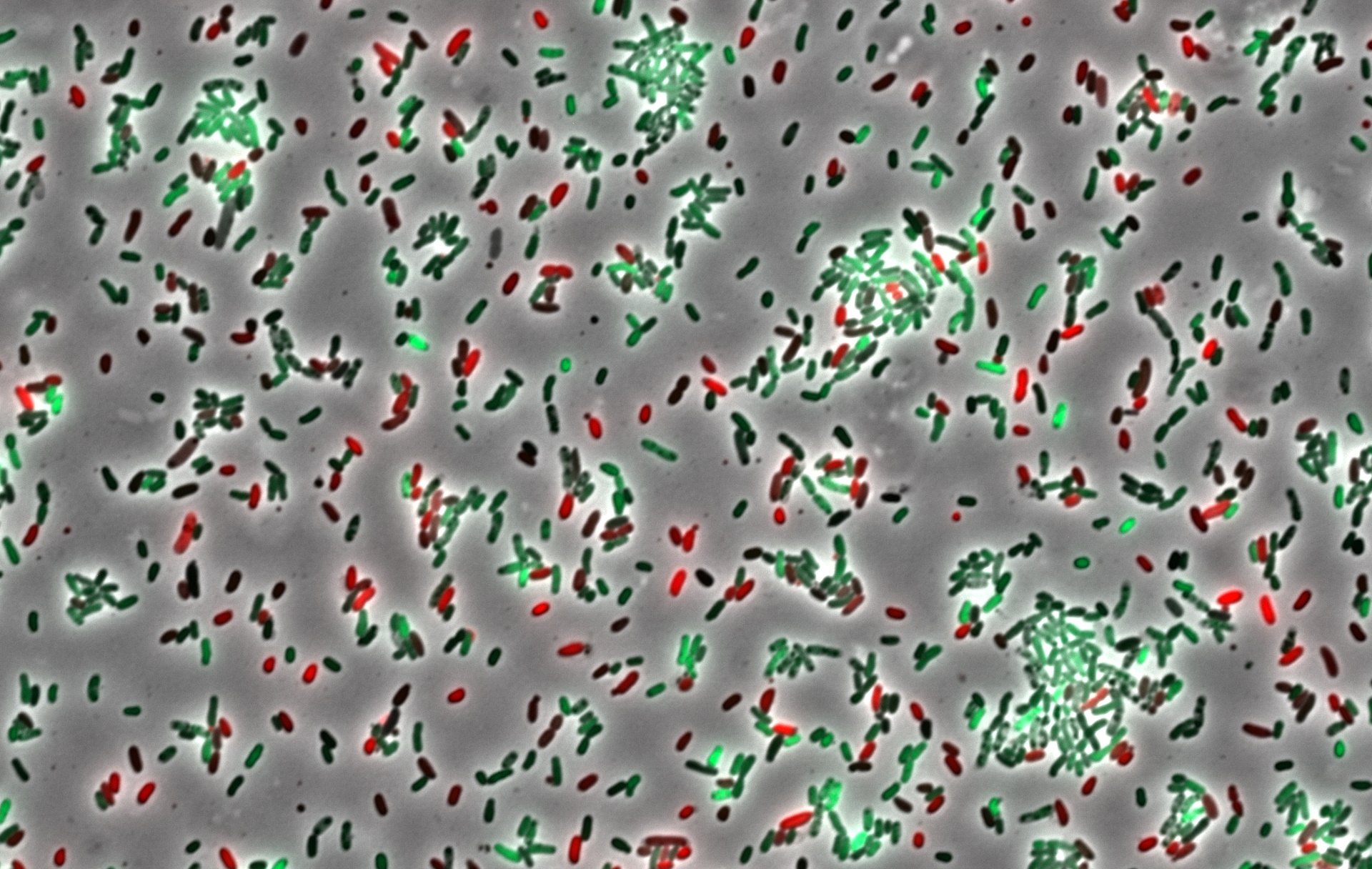

Supplement: Supplementary file 7 — Source Data for Figure 4 [file EMBR-24-e56849-s002.zip › 4A. Micr.image/repeat 3/C1-Tde1/C1-Tde1_2.jpg]

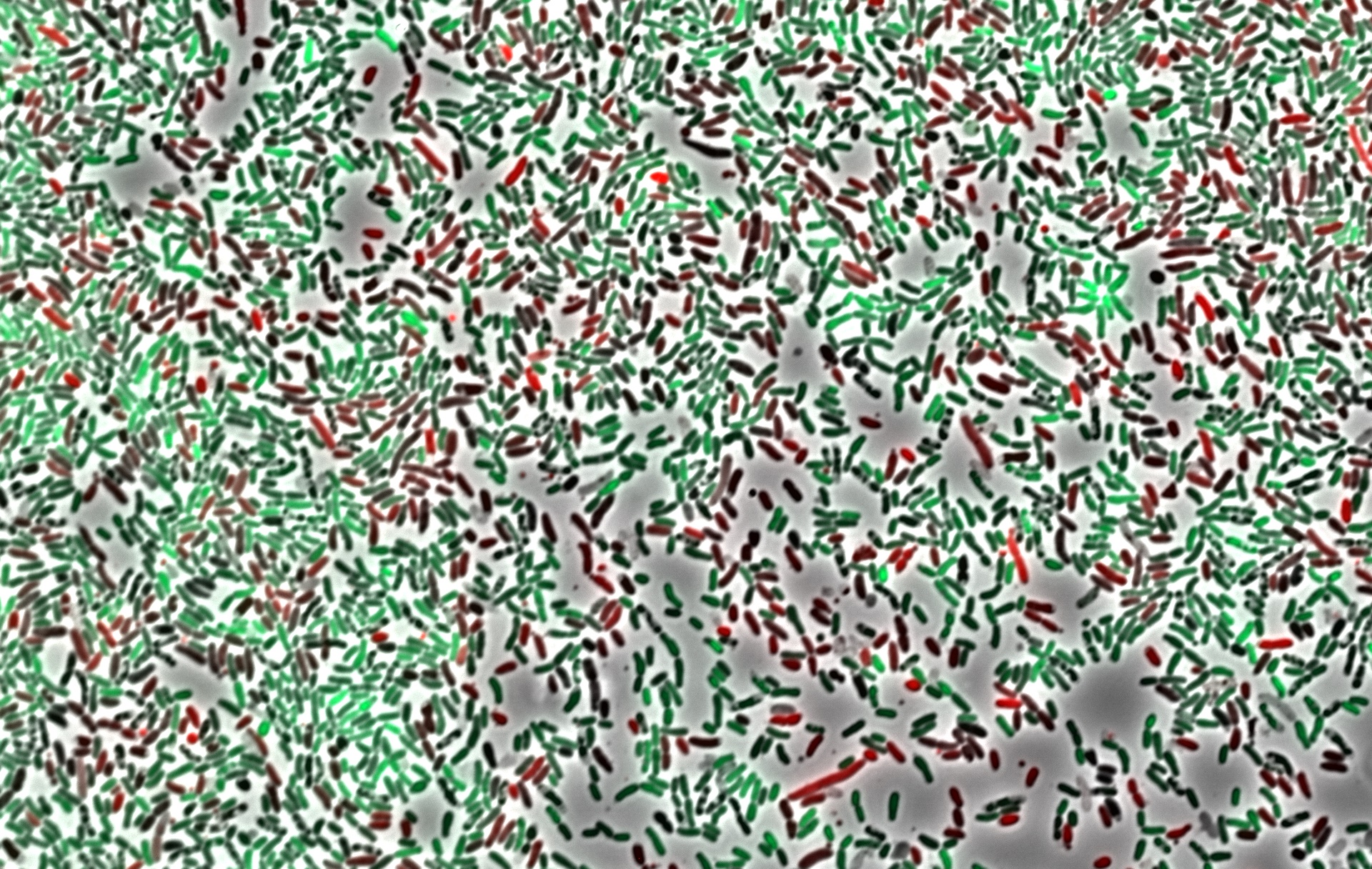

Supplement: Supplementary file 7 — Source Data for Figure 4 [file EMBR-24-e56849-s002.zip › 4A. Micr.image/repeat 3/Tde1(M)GLGL/Tde1(M)GLGL_1.jpg]

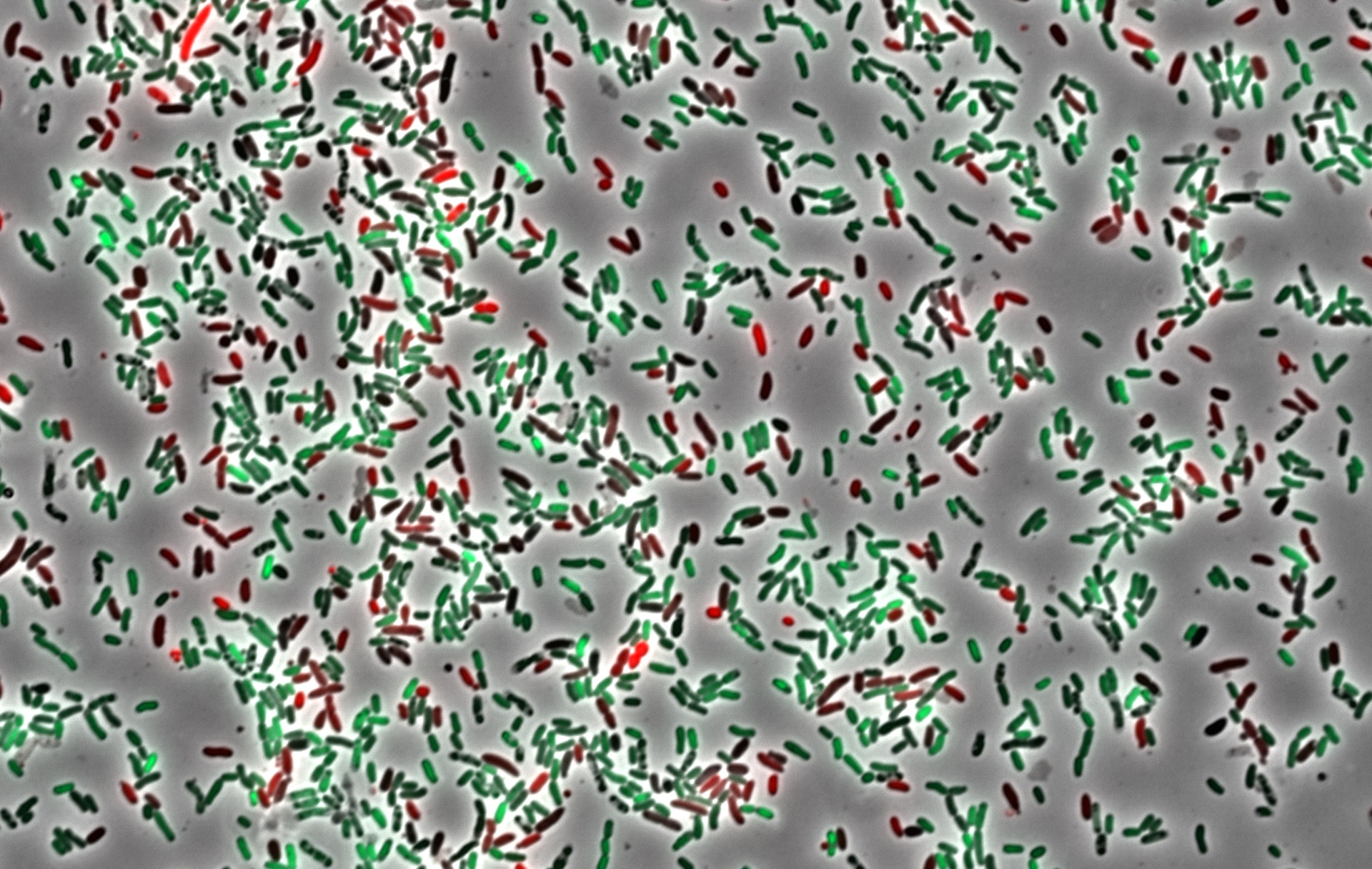

Supplement: Supplementary file 7 — Source Data for Figure 4 [file EMBR-24-e56849-s002.zip › 4A. Micr.image/repeat 3/Tde1(M)GLGL/Tde1(M)GLGL_2.jpg]

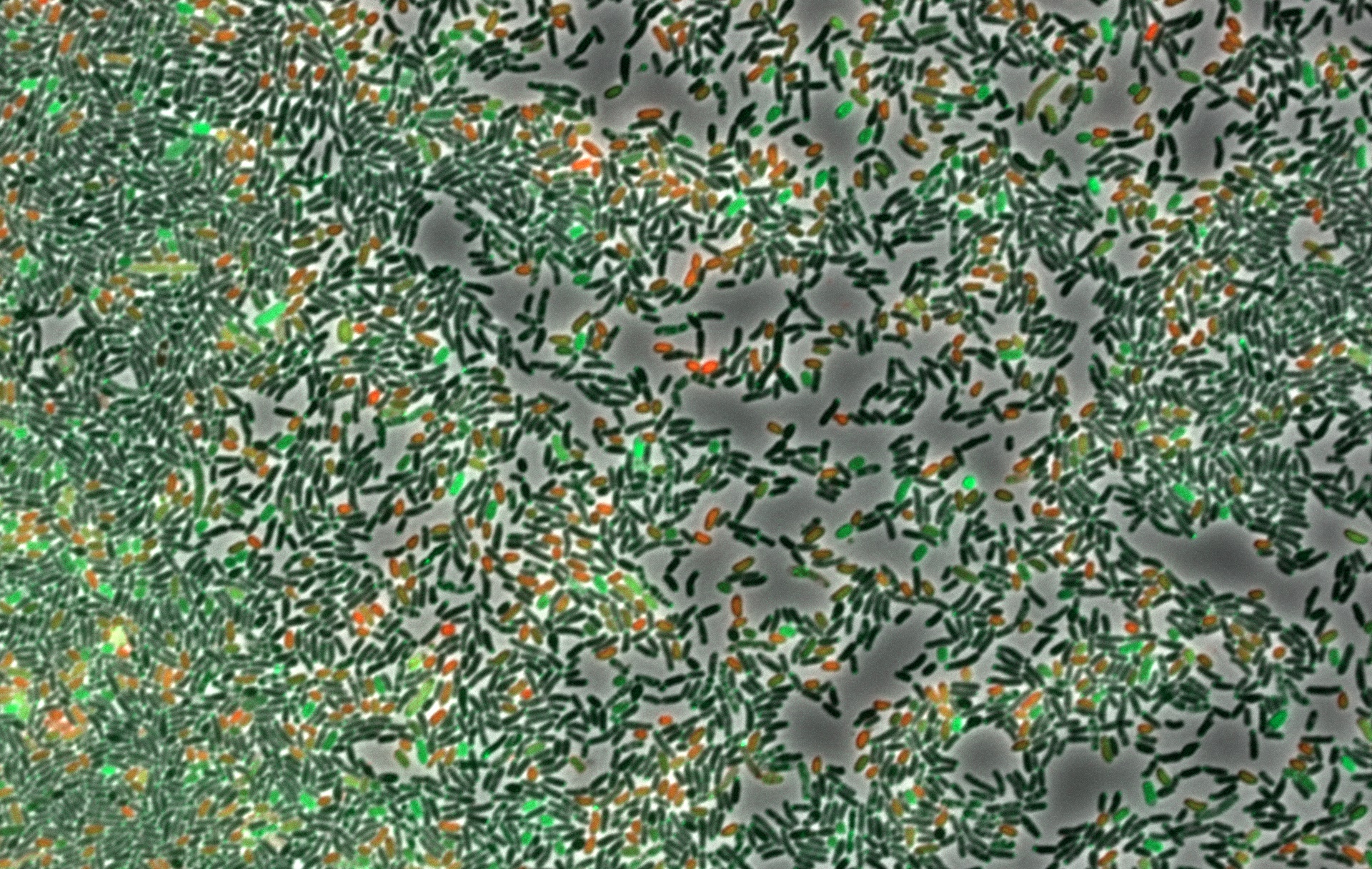

Supplement: Supplementary file 7 — Source Data for Figure 4 [file EMBR-24-e56849-s002.zip › 4A. Micr.image/Repeat 2/Tde1(M)/Tde1(M)_1.jpg]

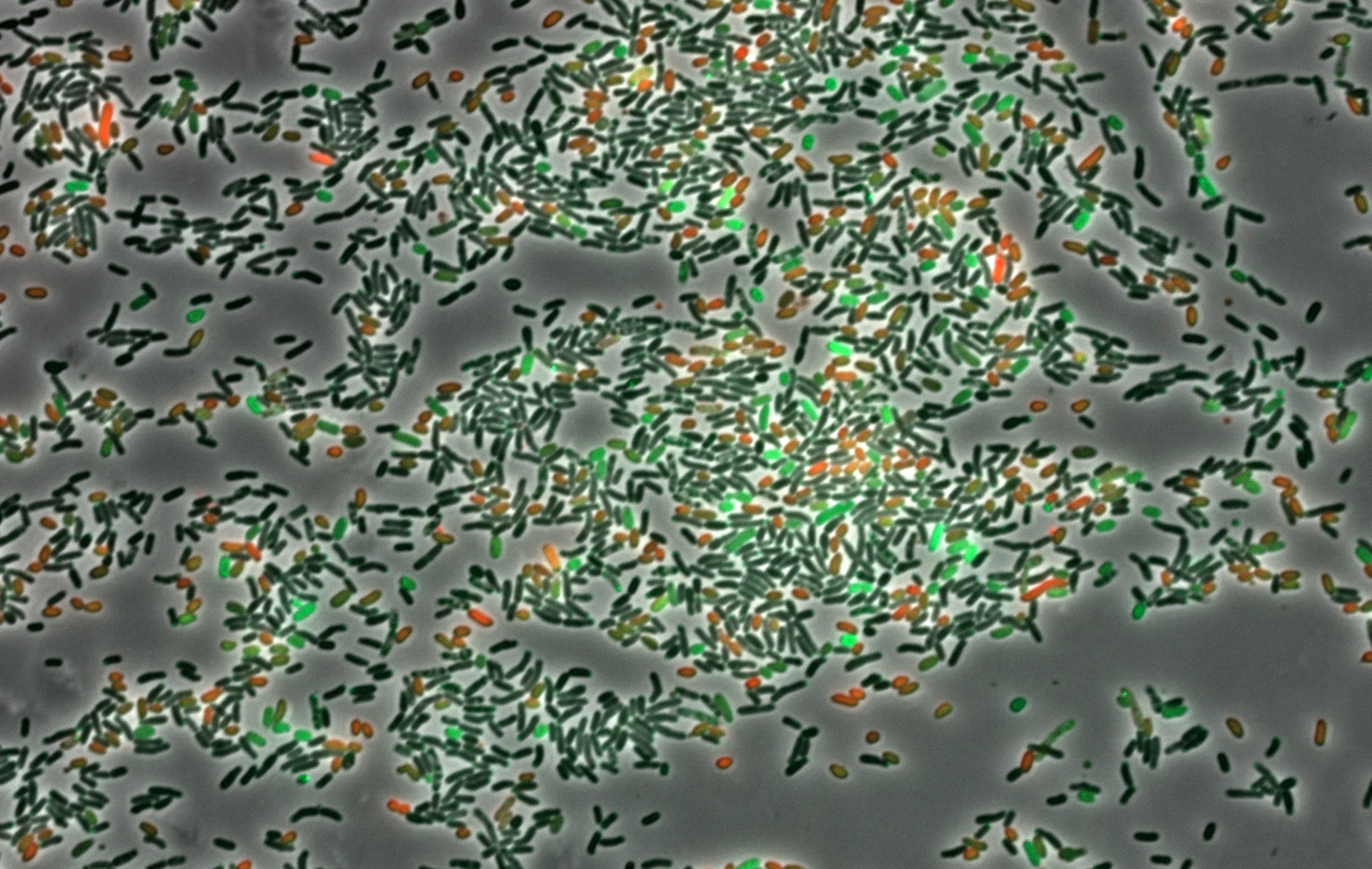

Supplement: Supplementary file 7 — Source Data for Figure 4 [file EMBR-24-e56849-s002.zip › 4A. Micr.image/Repeat 2/Tde1(M)/Tde1(M)_2.jpg]

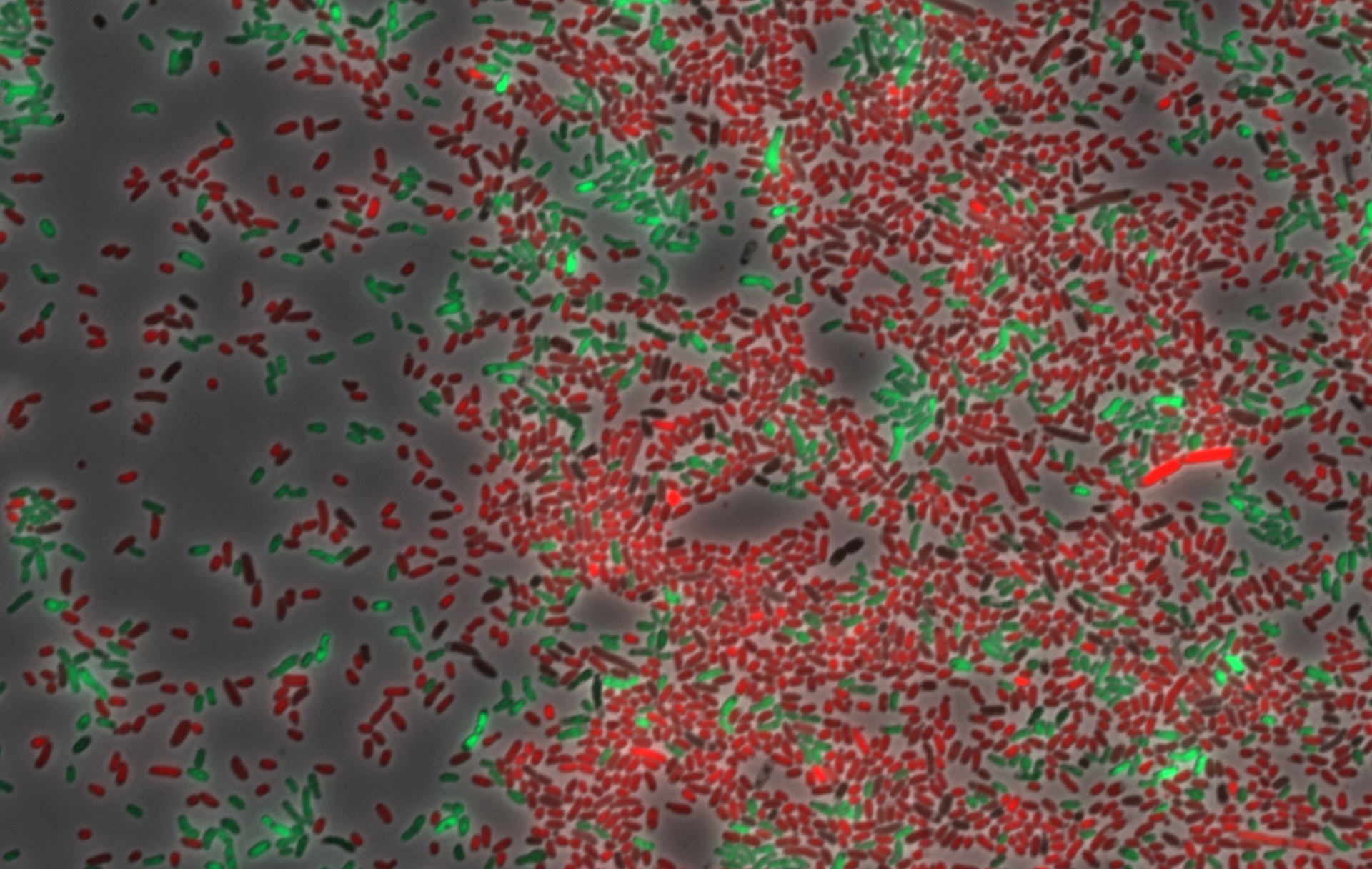

Supplement: Supplementary file 7 — Source Data for Figure 4 [file EMBR-24-e56849-s002.zip › 4A. Micr.image/Repeat 2/dtdei dtssk N-Tde1/Snap-1689_c1+2+3.jpg]

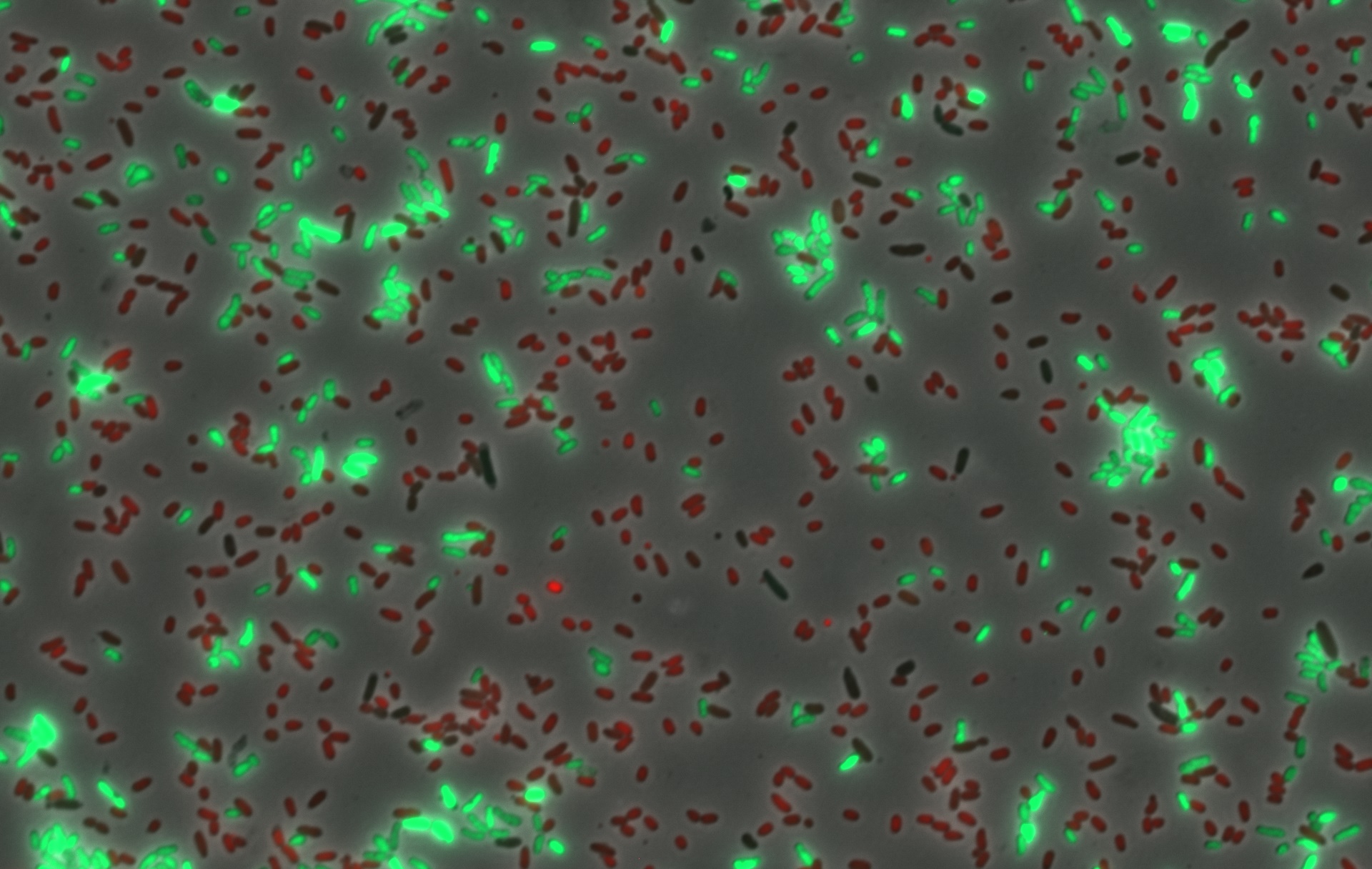

Supplement: Supplementary file 7 — Source Data for Figure 4 [file EMBR-24-e56849-s002.zip › 4A. Micr.image/Repeat 2/dtdei dtssk N-Tde1/Snap-1690_c1+2+3.jpg]

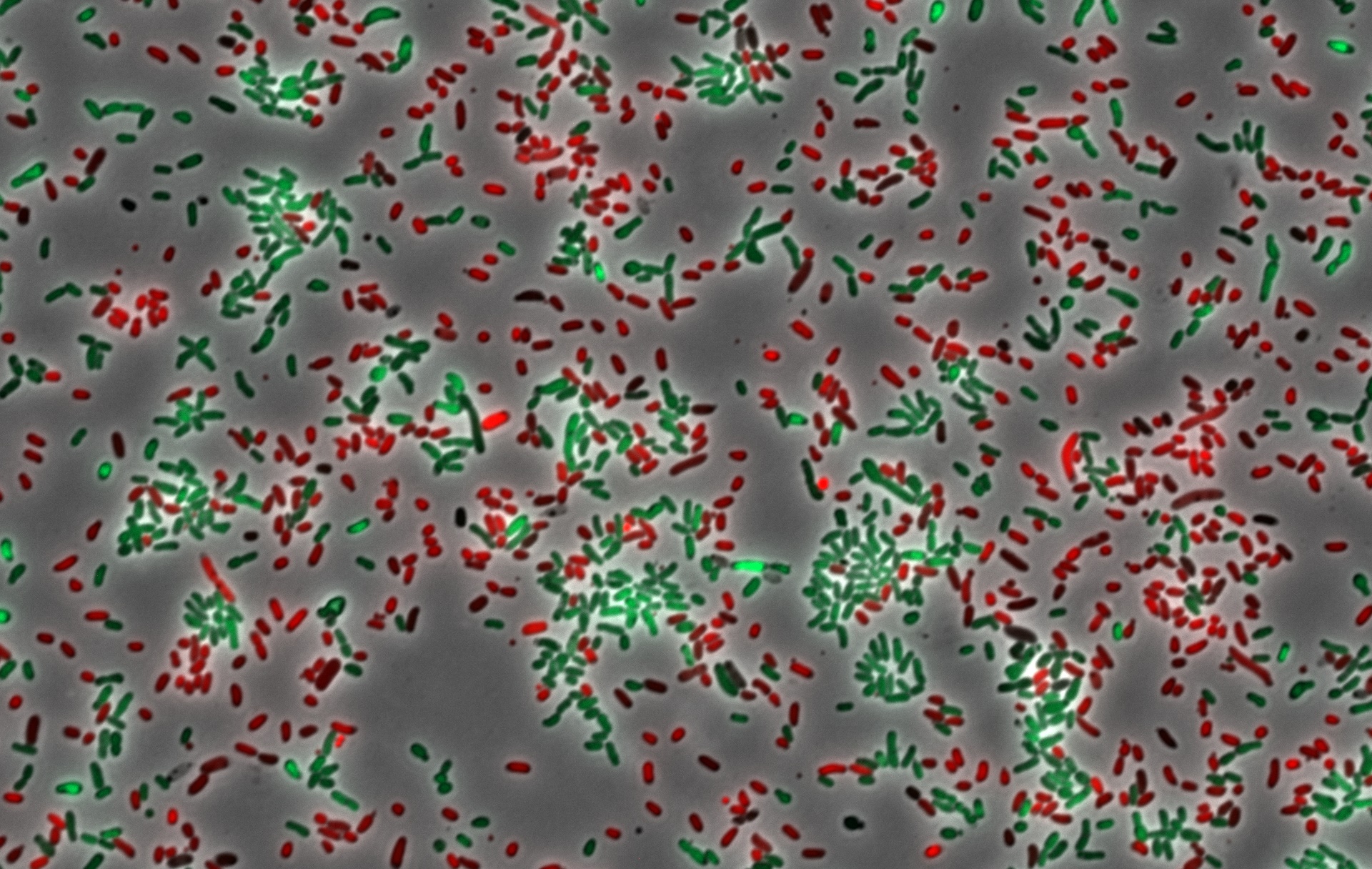

Supplement: Supplementary file 7 — Source Data for Figure 4 [file EMBR-24-e56849-s002.zip › 4A. Micr.image/Repeat 2/dtdei dtssk (Tde1(M))/dtdei dtssk (Tde1(M))_2.jpg]

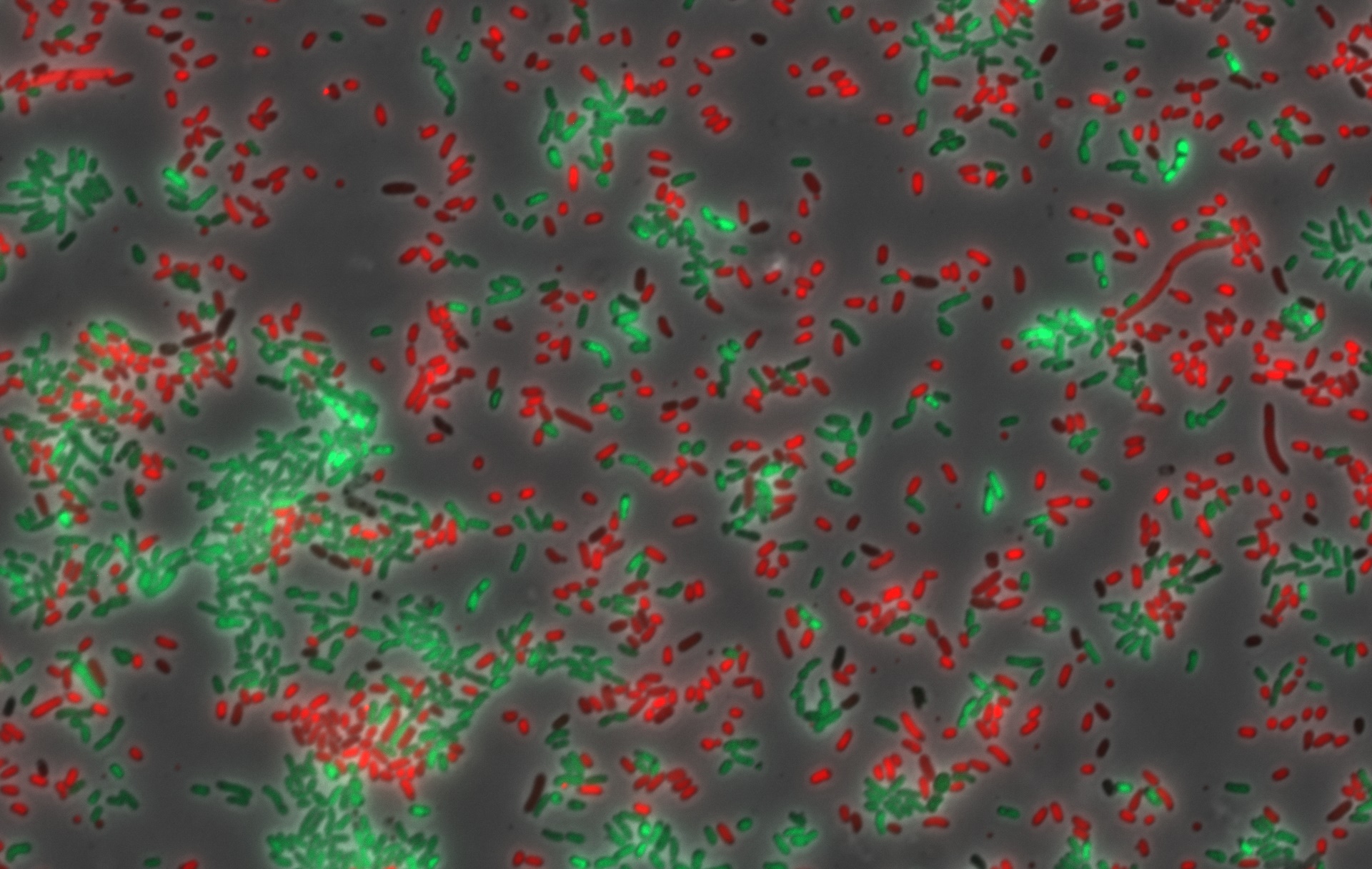

Supplement: Supplementary file 7 — Source Data for Figure 4 [file EMBR-24-e56849-s002.zip › 4A. Micr.image/Repeat 2/dtdei dtssk (Tde1(M))/dtdei dtssk (Tde1(M))_1.jpg]

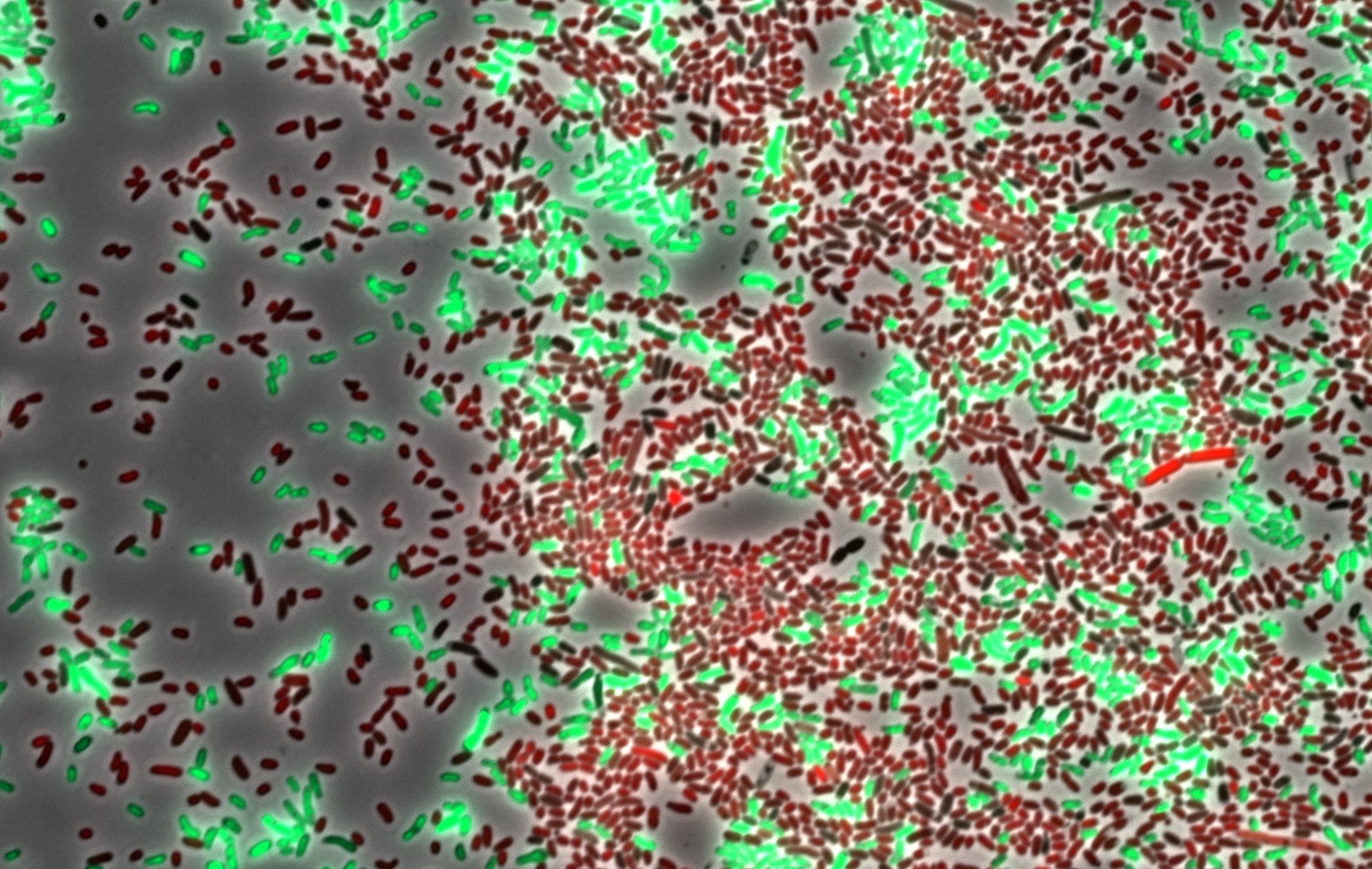

Supplement: Supplementary file 7 — Source Data for Figure 4 [file EMBR-24-e56849-s002.zip › 4A. Micr.image/Repeat 2/sfGFP/sfGFP_1.jpg]

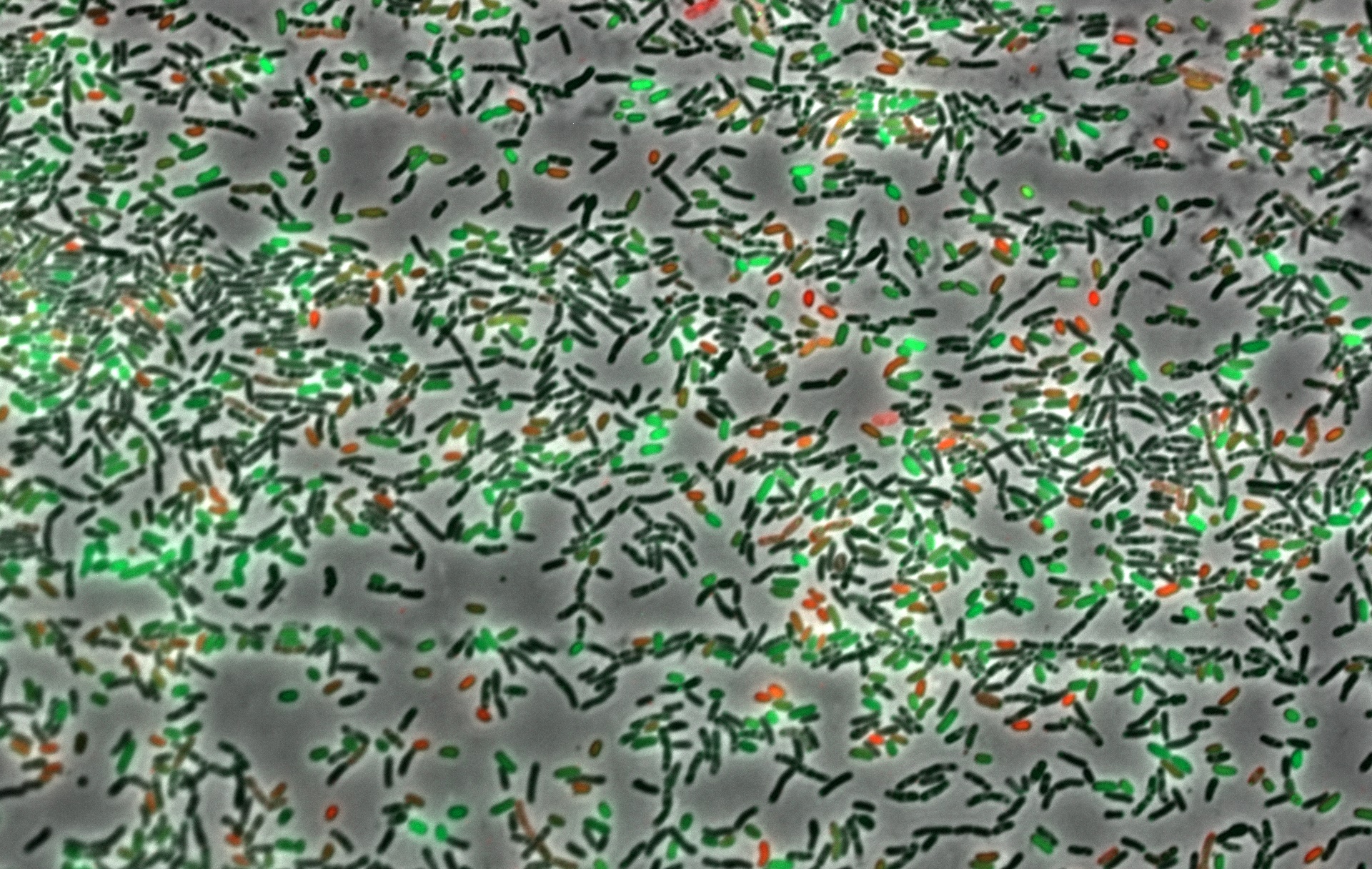

Supplement: Supplementary file 7 — Source Data for Figure 4 [file EMBR-24-e56849-s002.zip › 4A. Micr.image/Repeat 2/N-Tde1/N-Tde1_1.jpg]

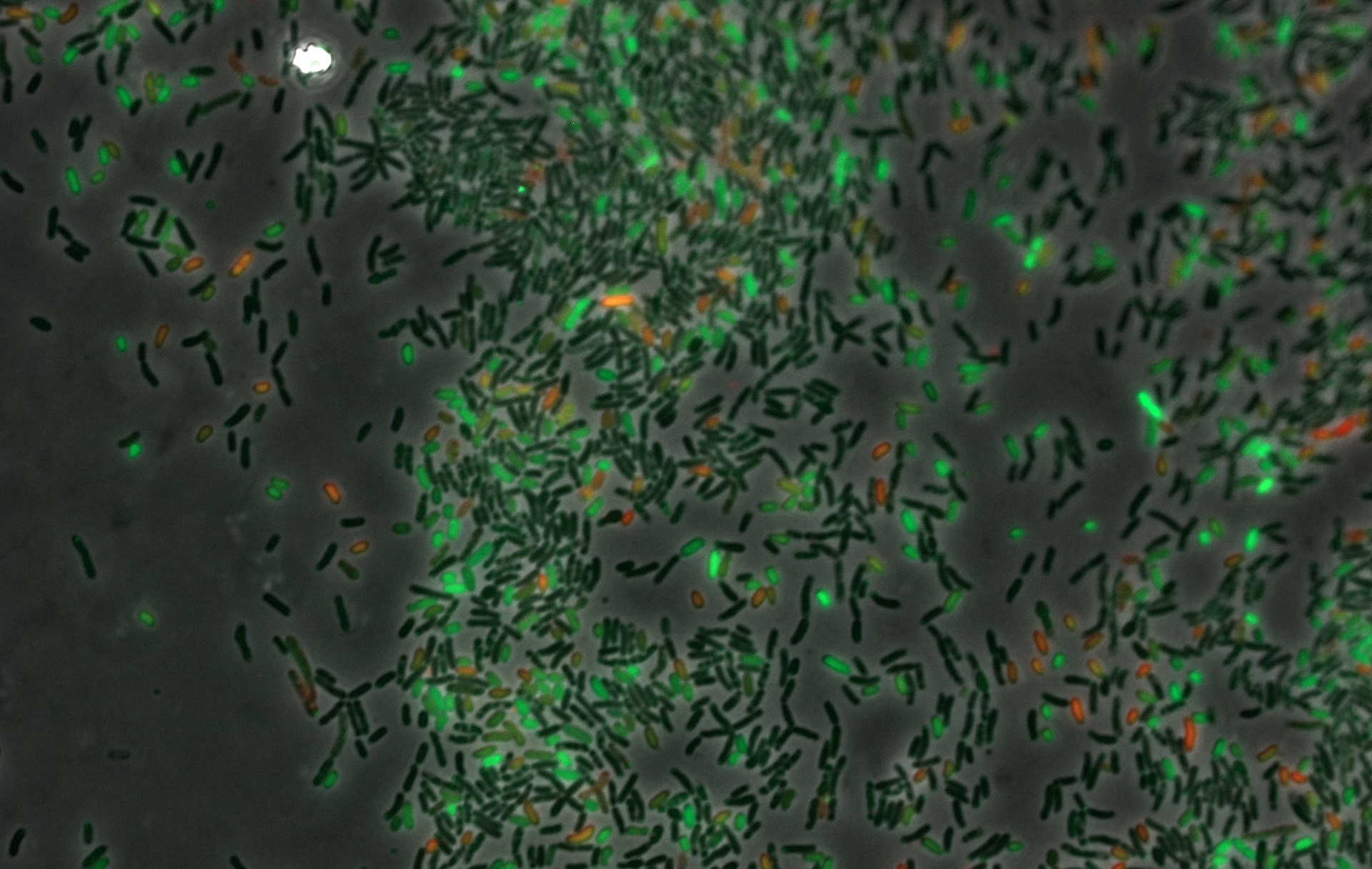

Supplement: Supplementary file 7 — Source Data for Figure 4 [file EMBR-24-e56849-s002.zip › 4A. Micr.image/Repeat 2/N-Tde1/N-Tde1_2.jpg]

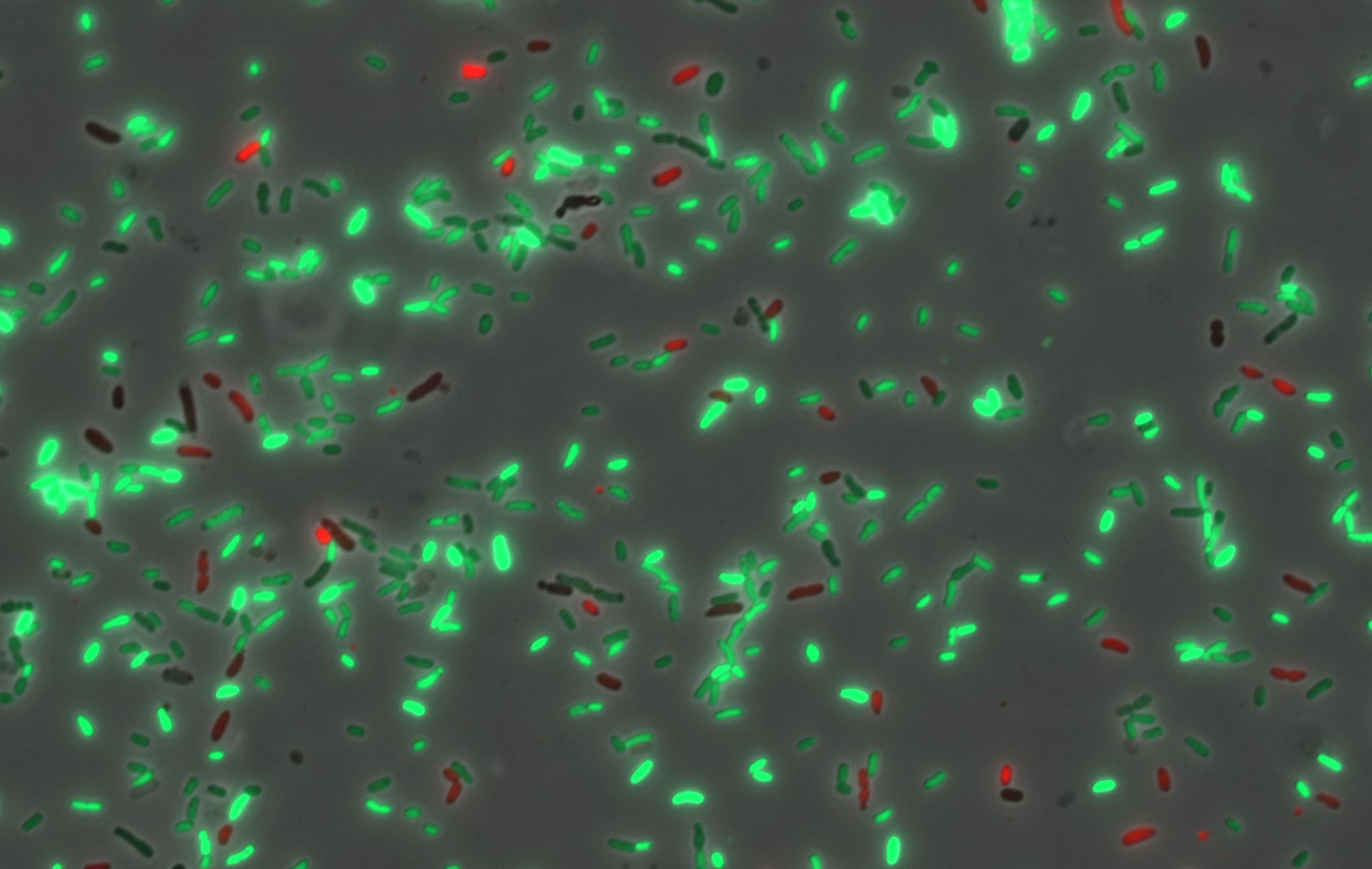

Supplement: Supplementary file 7 — Source Data for Figure 4 [file EMBR-24-e56849-s002.zip › 4A. Micr.image/Repeat 2/N-Tde1GLGL/N-Tde1GLGL_1.jpg]

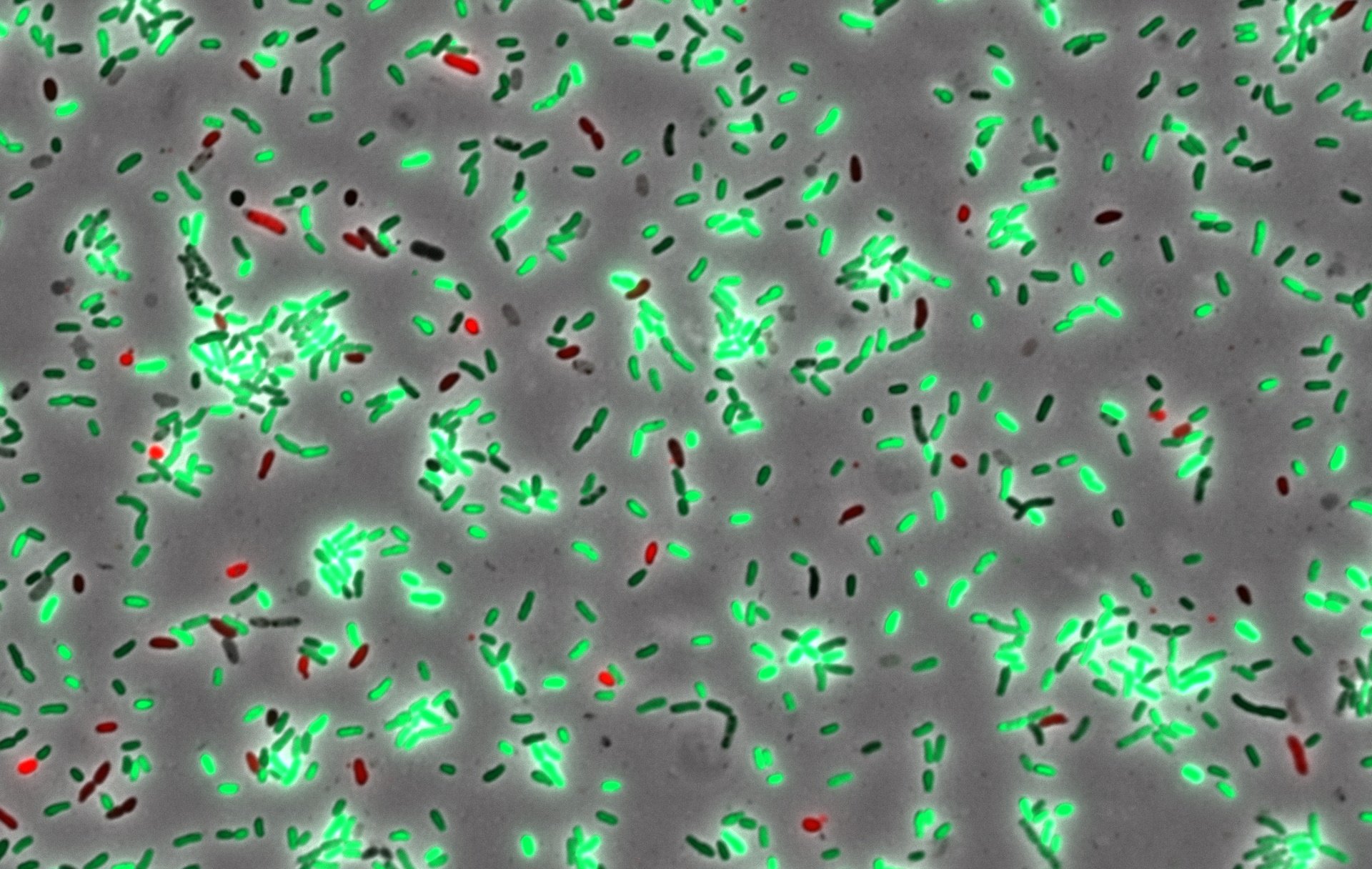

Supplement: Supplementary file 7 — Source Data for Figure 4 [file EMBR-24-e56849-s002.zip › 4A. Micr.image/Repeat 2/N-Tde1GLGL/N-Tde1GLGL_2.jpg]

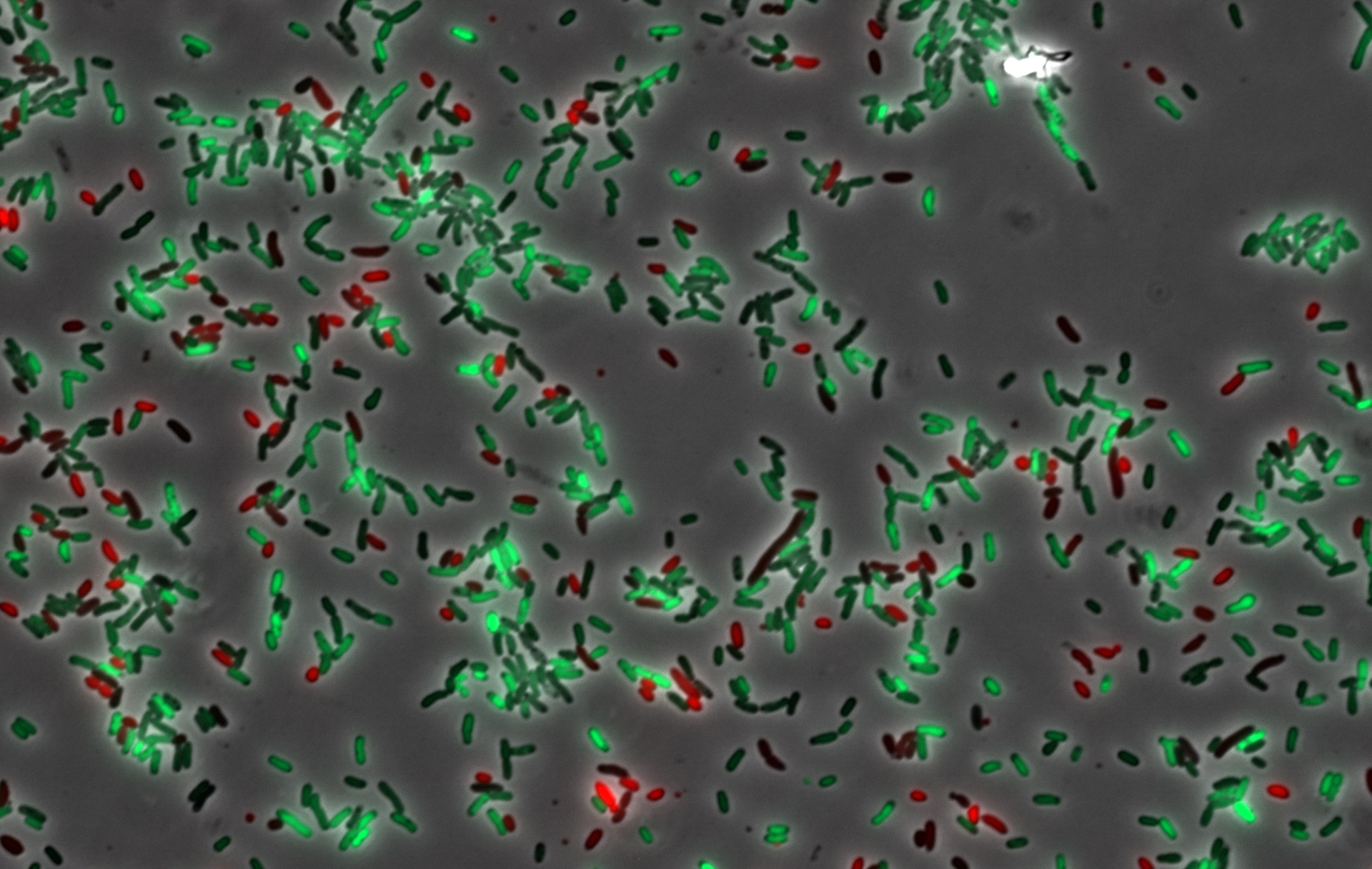

Supplement: Supplementary file 7 — Source Data for Figure 4 [file EMBR-24-e56849-s002.zip › 4A. Micr.image/Repeat 2/C1-Tde1/C1-Tde1_1.jpg]

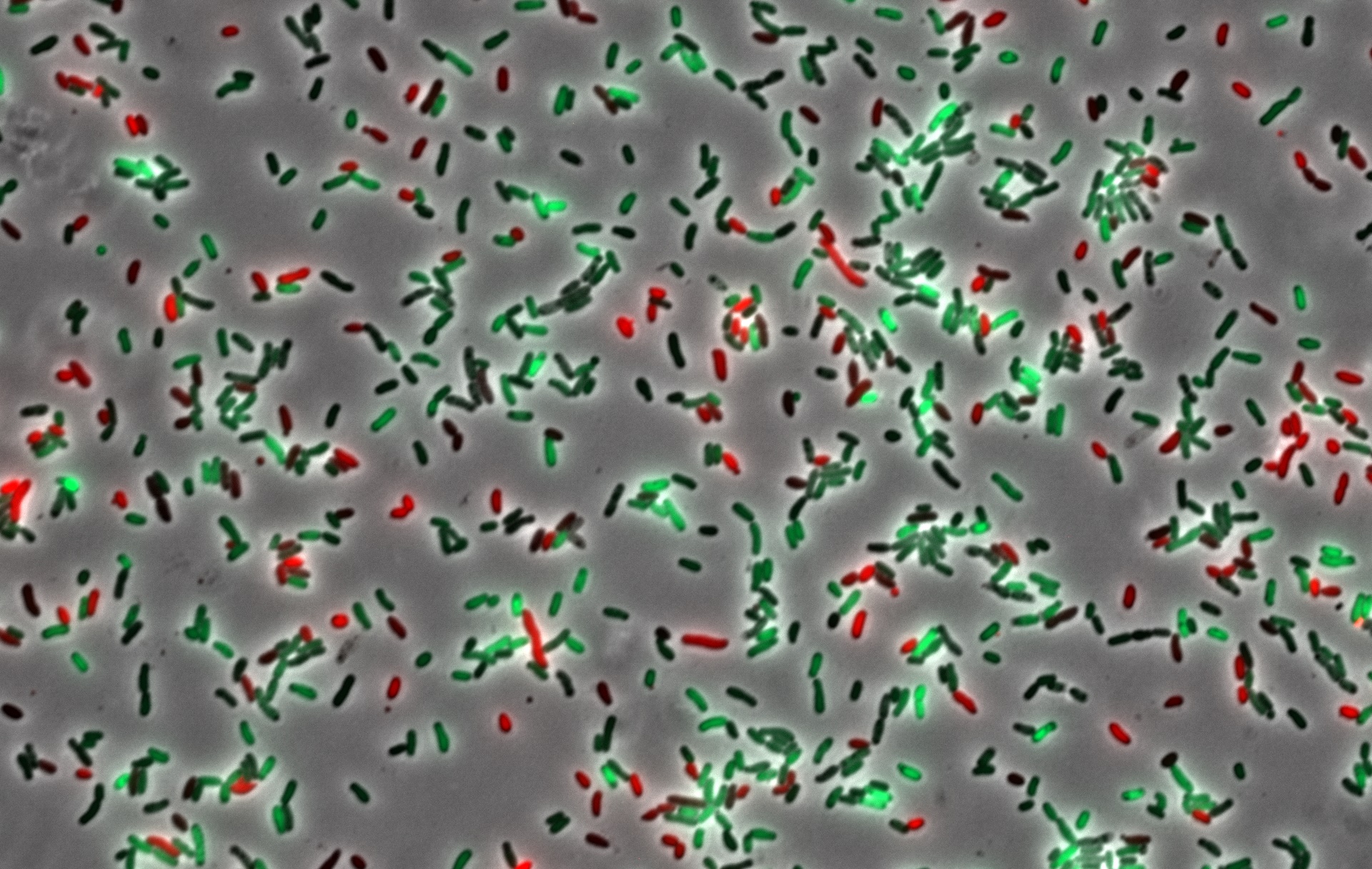

Supplement: Supplementary file 7 — Source Data for Figure 4 [file EMBR-24-e56849-s002.zip › 4A. Micr.image/Repeat 2/C1-Tde1/C1-Tde1_2.jpg]

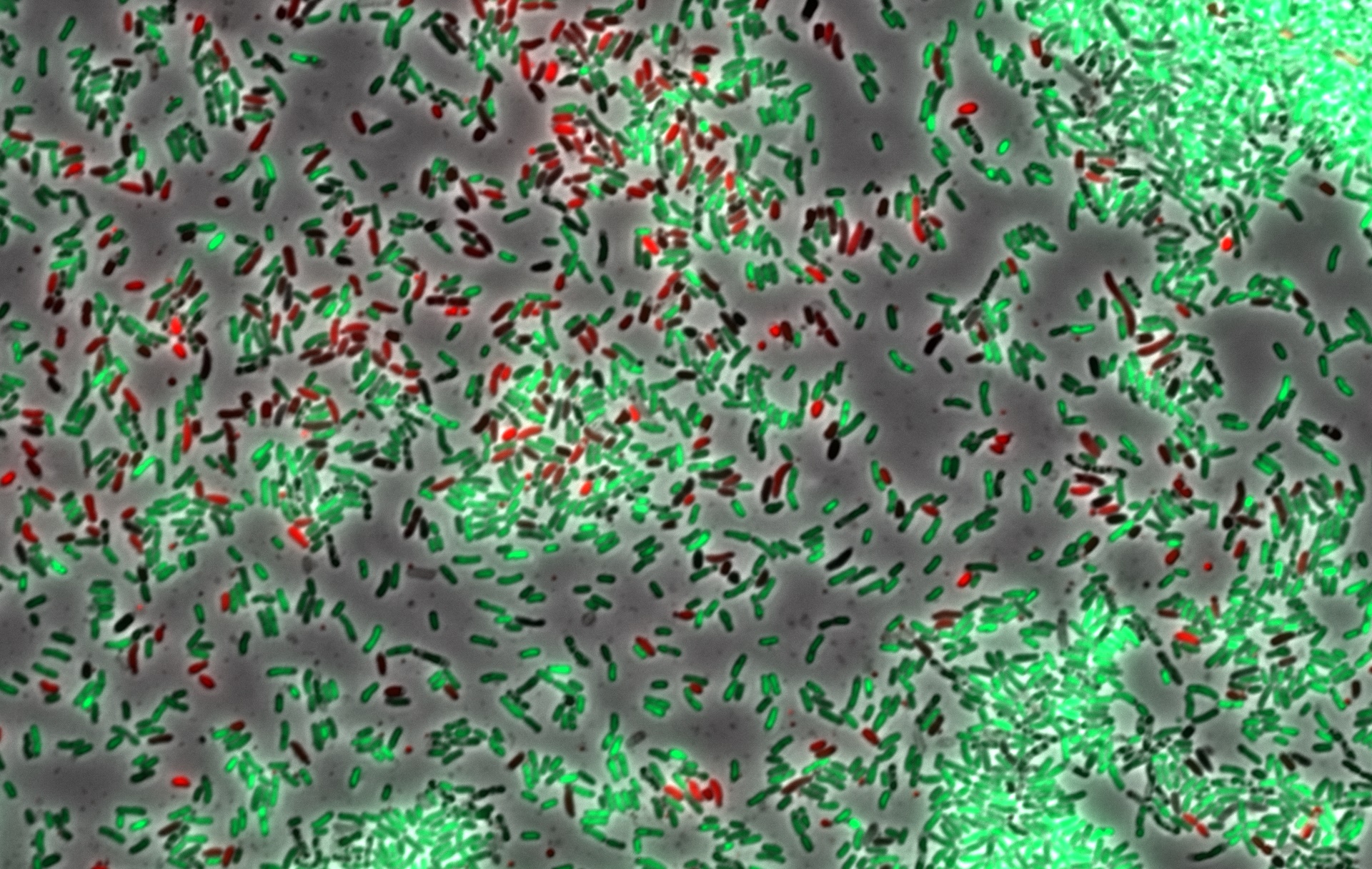

Supplement: Supplementary file 7 — Source Data for Figure 4 [file EMBR-24-e56849-s002.zip › 4A. Micr.image/Repeat 2/Tde1(M)GLGL/Tde1(M)GLGL_1.jpg]

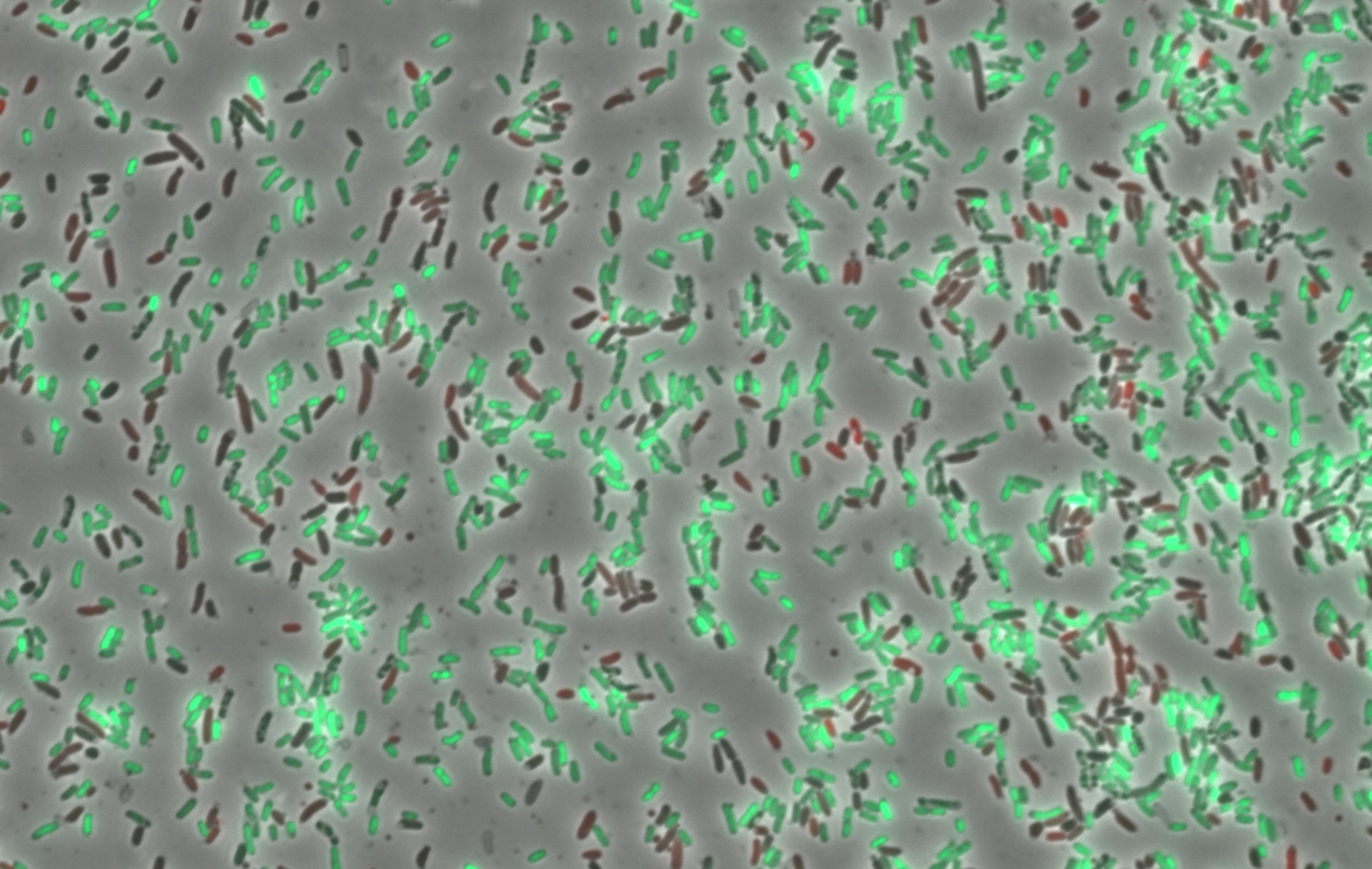

Supplement: Supplementary file 7 — Source Data for Figure 4 [file EMBR-24-e56849-s002.zip › 4A. Micr.image/Repeat 2/Tde1(M)GLGL/Tde1(M)GLGL_2.jpg]
