# Supplementary material for: A glycine zipper motif is required for the translocation of a T6SS toxic effector into target cells
Source: EMBO Rep. 2023 Apr 17;24(6):e56849. doi: 10.15252/embr.202356849 (PMC10240207; doi:10.15252/embr.202356849)
Supplement: Supplementary file 9 [file EMBR-24-e56849-s003.pdf]

EMBO reports

A glycine zipper motif is required for the translocation of a T6SS toxic effector into target cells

Jemal Ali, Manda Yu, Li-Kang Sung, Yee-Wai Cheung, and Erh-Min Lai
DOI: 10.15252/embr.202356849

Corresponding author(s): Erh-Min Lai (emlai@gate.sinica.edu.tw)

Review Timeline:

Transfer from Review Commons:	18th Jan 23
Editorial Decision:	7th Feb 23
Revision Received:	1st Mar 23
Editorial Decision:	8th Mar 23
Revision Received:	13th Mar 23
Accepted:	22nd Mar 23

Editor: Achim Breiling

Transaction Report: This manuscript was transferred to EMBO reports following peer review at Review Commons.

(Note: With the exception of the correction of typographical or spelling errors that could be a source of ambiguity, letters and reports are not edited. Depending on transfer agreements, referee reports obtained elsewhere may or may not be included in this compilation. Referee reports are anonymous unless the Referee chooses to sign their reports.)

Review #1

1. Evidence, reproducibility and clarity:

Evidence, reproducibility and clarity (Required)

Summary:

In this manuscript, Ali et al. propose that a glycine zipper motif located at the N-terminus of the Agrobacterium tumefaciens T6SS DNase effector, Tde1, can transport the toxin across the cytoplasmic membrane and into the cytoplasm, where its target is found. To support these claims, they perform a series of secretion, competition, toxicity, and fluorescence microscopy assays showing that a mutation in two glycine residues affects toxicity of the effector during competition and its ability to enter a target cell, but not its secretion through the T6SS or its binding to the adaptor protein Tap1.
The concept brought forth in this study is quite interesting and important - the notion that T6SS effectors have domains that aid in their transport into the cytoplasm of the target cell. This is similar to a recent finding that a domain common to bacterial pyocins and T6SS effectors can mediate DNase toxin transport through the target cell's cytoplasmic membrane (Atanaskovic et al., mBio, 2022); the authors should mention and discuss this recent work. Nevertheless, it is my impression that the results do not fully support the conclusions and proposed mechanism, even though the general idea seems correct.

Major comments:

- An experiment that directly demonstrates the ability of the glycine zipper to mediate transport of a toxin across a membrane would greatly support and solidify the conclusions of this work. For example, showing the ability of a purified protein to enter spheroplasts or liposomes in a glycine zipper dependent fashion. Currently, the authors perform experiments that can only indirectly support the proposed function of the glycine zipper to enable the effector to cross the membrane, and as detailed below, some of these experiments are over-interpreted in my opinion.
- Lines 153-159: It is not clear how much these results are relevant to the activity of the glycine zipper motif during effector delivery by the T6SS. If I understand correctly, the described experiments are of over-expression of the proteins in the E. coli cytoplasm, where glycine zipper-dependent membrane permeability and toxicity are detected. However, one would expect that if the effector is to be transported from the periplasm to the cytoplasm during T6SS delivery, then the glycine zipper should function from the periplasmic face of the cytoplasmic membrane, and not from its cytoplasmic face, as is the case in these experiments. Is it possible that the observed toxicity and membrane permeability be the result of over-expression in the "wrong place"?
- Fig. 4B: This figure appears to be very important, and the authors base a large part of their main conclusion regarding the role of the glycine zipper in membrane crossing on it. However, some controls are missing and part of the results observed in the figure do not match their description in the text.
- Lines 233-237 - While the authors state in the text that GFP and mCherry signals did

not overlap in E. coli cells co-cultured with Agrobacterium cells expressing Tde1(M)-GLGL, I see many double-colored cells in this sample (bottom panels in Fig. 4B). Actually, all cells appear to have both green and blue colors, except for a few cells that are only green but that also seem to be dead judging by their ghostly appearance in the phase contrast channel.
- How is it that all cells in the bottom panels are blue (indicating that they are E. coli target cells)? Shouldn't a large portion of the cells be Agrobacterium cells that should not be blue, since these are added at the beginning of the competition assay at a 10:1 ratio in their favor?
- It is quite remarkable that so much GFP signal is transported into the E. coli target cells so that it is so clearly visible under the microscope. How do the authors know that the GFP signal overlapping with the mCherry is really inside the cell and not outside (for example, proteins secreted to the media that attach to the cell envelope)? Will the GFP signal remain if trypsin is added to the media before visualization under the microscope?
- Can the authors quantify the ratio of E. coli cells that have overlapping green and blue colors over several experiments for each sample, to show that this phenomenon repeats and is statistically significant?
- Can the authors explain why at least some of these E. coli cells should not be dead due to the toxicity mediated by the third effector of the Agrobacterium T6SS, Tae?
- Why were the microscopy competitions performed differently than the regular competition assays? Why wasn't AK media used in these competitions? How active is the T6SS under these conditions compared to the AK media?
- In the N-Tde1 sample, many Agrobacterium cells appear to have the GFP signal in foci rather than distributed throughout the cell (as it is in other samples), while the E. coli cells have a uniform and strong GFP signal. Can the authors comment on that?
- It might be easier for readers to visualize this figure and see the signal distribution in the different cells if the authors show a zoomed in version in the main text, and provide the wide field images as a supplementary figure.
- Fig. 5C-D: The reduced expression and secretion of the GLGL mutant is considerable. How can the authors rule out that this reduction was the cause for the reduced observed toxicity of the mutant in 5A-B? Moreover, the results show that the GLGL double mutant is hampered in expression, secretion, and DNase activity, and it negatively affects overall T6SS activity. Since this mutant was used throughout the paper, and in the absence of a direct assay showing membrane transport mediated by the glycine zipper motif, the claim of the role of this motif in membrane crossing is not well substantiated by the results. If the authors were to show that the single glycine mutants used in Fig. 5D, which are stable and have an intact DNase activity, behave as claimed in the final conclusion sentence (lines 279-283), then the conclusions will be better substantiated by the results.

Minor comments:

- Line 93: I am not sure that Ntox15 should still be referred to as a "novel" domain.
- Line 105: The section's heading does not actually describe its content. The results here only show toxicity upon over-expression of the effector or its mutant forms in E. coli. Therefore, this cannot be referred to as a "prey cell" since the effector was not

transported into it during competition. Moreover, the results in Fig. 5A do not support DNase-independent toxicity during competition.
- Please consider making all of the symbols in the growth assays semi-transparent. It is impossible to discern between the different, overlapping curves.
- Please consider making the size bars in all microscopy images more pronounced. They are barely visible in their current form. Also, it would be better to show images of the same magnification/zoom for the different samples, since the current presentation shows cells from different samples at different sizes, and it can be confusing to the readers.
- In Fig. 1B and in Fig. 2A the authors show that expression of Tde1(M) in cells is toxic, yet in Fig. 2D they see no toxicity. Can the authors please comment on this discrepancy?
- I am not convinced that the assay in Fig. 2E can be used to determine bacteriostatic/bacteriolytic effect. It is not clear how such a distinction can be made from OD measurements, since an increase in OD can result from the entire population growing after removal of the stressor, or just part of the population that did not lyse/die. To make such a claim, the authors can spot bacteria on repressing media at different timepoints after protein induction, and then determine CFU.
- Fig. 3A: A control is missing. To verify that the N-terminal part of Tde1 is not promiscuously interacting with proteins, the authors should include a control sample testing its inability to precipitate a protein other than Tap1 in the same experiment.
- Fig. 3B: the blots are very "dirty". It is not clear how the authors were able to determine expression and precipitation of some truncations (for example, C2-Tde1 in the E. coli IP panel looks like a background band found in other lanes too).
- Lines 222-225 (Fig. 4A): I can't see C-1-Tde1(M)-sfGFP in the cellular blot. All the bands in this lane look like background bands that are also present in all other lanes. Therefore, I am not sure how the conclusion regarding this truncation's ability to be secreted was reached.
- Fig. 4A: the protein names above the lanes should include the sfGFP that is fused to them.
- It would be preferable to show quantitative competition assays with statistics rather than pictures of a plate showing a single competition result, if conclusions or observations on minor differences in toxicity are made (for example, line 253: "The killing activity of Δtdei(Tde1GLGL-Tdi1) was largely compromised"). Since the authors performed each competition assay more than once, these data should be available to them.
- Fig. 5A: The author claim at the beginning of the manuscript (first results section heading: "Tde1 can cause DNase-independent growth inhibition of prey cells") that the N-terminal region of Tde1 is toxic on its own in the prey cell, yet in this competition assay Tdi1(M) shows no toxicity against the E. coli target cells. In the microscopy assay (Fig. 4B), it appears that a lot of Tdi1(M) enters the prey cell, since we can visualize it under the microscope. Can the authors clarify this discrepancy and explain why they do not expect to see target killing by this mutant even though they claimed it is toxic earlier?
- Fig. 5B: the GLGL mutant seems to have some residual toxicity, not dissimilar to what is shown in 5A. Why are these similar results interpreted differently (in 5A they are "largely compromised", while in 5B "killing activity... was not detectable")? Also, why was Tde(M)1-Tdi1 used in Fig. 5A but Tdi1(M) without the immunity gene used in Fig. 5B?
- Fig. 5: Does the remaining third effector, Tae, not play a role in these competition

assays? If, as shown in Fig. 5C, the entire T6SS is less active when a GLGL mutant is expressed, couldn't the different in toxicity shown in Figs. 5A-B be the result of lack of Tae secretion and toxicity?
- Lines 359-362: T6SS effectors that bind the inner Hcp tube were suggested to be only partially folded.

2. Significance:

Significance (Required)

The concept of T6SS effectors providing their own mechanism of transport from the cytoplasm to the periplasm is very interesting. It will appeal to audience in a wide range of microbiology disciplines, including those interested in toxins, membrane transport, and even translational applications. A similar concept was recently proposed and demonstrated for a domain that is also found in T6SS effectors (Atanaskovic et al., mBio, 2022).

Expertise: I have been studying the different aspects of T6SS for the past decade.

3. How much time do you estimate the authors will need to complete the suggested revisions:

Estimated time to Complete Revisions (Required)

(Decision Recommendation)

Between 3 and 6 months

4. *Review Commons* values the work of reviewers and encourages them to get credit for their work. Select 'Yes' below to register your reviewing activity at Web of Science Reviewer Recognition Service (formerly Publons); note that the content of your review will not be visible on Web of Science.

Web of Science Reviewer Recognition

Yes

Review #2

1. Evidence, reproducibility and clarity:

Evidence, reproducibility and clarity (Required)

This manuscript is focused on understanding how the Agrobacterium tumefaciens T6SS effector, Tde1, is translocated across the cell envelope of target cells and how this effector binds to the adapter Tap1. The authors show that GxxxG motifs in the N terminal region of Tde1 are required for delivery into the cytoplasm of target cells and permeabilising the cytoplasmic membrane. Given that these GxxxG motifs resemble glycine zipper structures that are found in proteins involved in membrane channel formation, the authors propose that these Tde1 motifs are involved in channel formation in the target cell. The authors also show that the N terminal region of Tde1 binds to Tap1 to facilitate loading onto the T6SS machinery but that the GxxxG motifs are not involved in this binding. Overall the manuscript was easy to read and followed a logical presentation of the findings. There are a few major comments that this reviewer has below - addressing these would allow the authors' claims to be more robustly supported.

Major comments:

1. Fig 1B: Why is this such a short growth experiment (5 hrs total with 2 hr pre and 3 hrs post induction)? Reporting on a growth experiment would normally be at least until the cells reach stationary phase but here the cells are still clearly in exponential phase. This reviewer would query what happens to growth rate in later exponential growth and into stationary phase? Is the toxic effect lessened in later stages of growth?
2. It is indeed surprising that C2-Tde1(WT) does not inhibit growth despite it having a functional DNase domain and being expressed in the cytoplasm. Did the authors confirm that this protein variant was expressed by Western blot or other means? This should be done to confirm that this variant is indeed not impacting upon growth instead of it not impacting growth simply because it is not being expressed.
3. The letters used to report significance are not clear to this reviewer. The authors say that "The significant differences were shown by the different letters (p value <0.01)" and then have a, b, c etc next to lines of the growth experiments in Figure 1B, C and Fig 2A, B, E etc. Which comparisons have a p value <0.01? this is not clear.
4. For all fluorescence microscopy experiments how many fields of view were imaged for each biological replicate? Were the fields selected at random or was the field selection biased to what was present in the field before taking the image? The answers to all of these questions should be stated in the methods. Also the microscopy data presented in the manuscript is not quantitative. Quantification of the number of cells with PI vs Hoechst signal (in Fig 2C) and mcherry vs gfp signal (in Fig 4B) for all fields of view and for all biological replicates would be very informative and convince the reader that the authors have not just "cherry picked" the images they are showing in the manuscript. This could be performed manually or the authors could use the freely available image analysis program Fiji (https://imagej.net/software/fiji/) to perform these analysis in a semi-automated manner.
5. For the co-IP experiments in Fig 3 where interaction between HA tagged Tde1 and Tap1 is demonstrated the authors should also show that Tap1 does not interact with a different HA-tagged protein i.e. that the interaction is specific to Tde1 and not the HA

motif.
6. For all Western blot images there should be at least 2 protein standard markers present in each individual blot - i.e. for Fig 3A and B the bottom panel showing Tap1 detection only has the 35 kDa marker, it should have at least one more marker in it. The same is true for other panels in Fig 2, 3 and 4. Having at least two molecular weight markers in a panel is now standard for most journals when presenting Western blot images.
7. For the competition assay serial dilution images in Fig 5A-B the images are a nice way to visually represent the experimental outcome but they should accompany graphs showing the competitive index of CFU/ml of the input prey and attacker vs the output prey and attacker for all biological replicates. This will convince the reader that the authors had equivalent amounts of the prey and the attacker going into the experiment and also that all attackers grew at the same rate and so were equally able to target the prey cell. This quantification could also provide more convincing out competition of ID1609 prey by C58 attacker (Fig 5B).

Minor comments:

Line 40: should read "...demonstrate that the effector itself..."

Line 41: "...we propose..." instead of "...we proposed..." since present tense makes more sense for this statement.

Line 51: "Each specialized protein secretion system" instead of "Each of...."

Line 76: "A glycine zipper structure..."

Line 79: "For example..."

Lines 96-100: The present tense should be used here as the current usage of past tense implies that this has been done in previous work and not in the current study - eg "we revealed", "we showed" would be better as "we reveal", "we show".

Fig 5B - The competition assay serial dilution images look a bit blurry, are there images the authors could use that are not blurry?

2. Significance:

Significance (Required)

This work is significant in as while there is a great deal known about how T6SS effectors cause toxicity there is less known about how these effectors are loaded onto the T6SS machinery and very little known about how T6SS effectors are able to translocate across the cytoplasmic membrane of target cells to reach a cellular component that is in the cytoplasm. This work would be of wide general interest to researchers in the T6SS field as well as those interested in bacterial secretion systems.

Reviewer expertise key words: Molecular microbiology, T6SS, interbacterial competition

3. How much time do you estimate the authors will need to complete the suggested revisions:

Estimated time to Complete Revisions (Required)

(Decision Recommendation)

Less than 1 month

4. *Review Commons* values the work of reviewers and encourages them to get credit for their work. Select 'Yes' below to register your reviewing activity at Web of Science Reviewer Recognition Service (formerly Publons); note that the content of your review will not be visible on Web of Science.

Web of Science Reviewer Recognition

Yes

Review #3

1. Evidence, reproducibility and clarity:

Evidence, reproducibility and clarity (Required)

Summary:

In this work, Ali et al. demonstrate that the N-terminal GxxxG motif of the T6SS DNase effector Tde1 of Agrobacterium tumefaciens is required for interbacterial intoxication. Using a combination of cell viability, reporter, and microscopy assays, the authors demonstrate that over-expression of the N-terminus of Tde1 results in inner membrane permeability. Moreover, the authors show that both the interaction between Tde1 and its adaptor Tap1 as well as the T6SS-mediated secretion of Tde1 are dependent on the N-terminus of Tde1. Finally, using a combination of in vitro and in vivo experiments, the authors determine that the N-terminal GxxxG motif is essential to Tde1-dependent

interbacterial killing by enabling effector entry into competing bacterial cells.

Major comments:

If N-tde1 is 1-97 aa, the predicted size is 9 kDa, but it shows up as ~17 kDa? Can the authors comment on this? Does N-tde1 or tde1 dimerize?

I have many concerns with the data and conclusions drawn from the data in Fig. 3B. I recommend removing it since (1) the data are not accurately represented in the text and (2) it is difficult to ascertain whether biologically relevant conclusions can be drawn from what happens with Agrobacterium proteins in E. coli. Below is a summary of my concerns regarding this section: I disagree with the authors' statements in lines 191-198. Their pulldown with E. coli is not consistent with their pulldown in C58. In fact, given the expression problems of some of the constructs in E. coli, I believe the data shown in Fig. 3B is inconclusive. The amount of Tap1 that co-IP'ed with N-Tde1GLGL and Tde1(M) is very low even though the expression levels of N-Tde1GLGL and Tde1(M) were relatively strong. Therefore, I do not feel confident concluding that these proteins "interact". Secondly, Tde1(M)GLGL was not expressed in E. coli, so no conclusions can be drawn. Moreover, the C1 and C2 variants were also not expressed well, so I believe the authors' statement in line 191-192: "Similar to the results in A. tumefaciens, the N-Tde1 and Tde1(M) interacted with Tap1 but not the C-terminal variants", is unjustified. You cannot rule out that C1 and C2 do not interact with Tap1 because C1 and C2, like Tde1(M)GLGL, were not expressed well in E. coli.

Lines 211-214: It looks like C1-Tde1(M) inhibits T6SS secretion. I am aware that in Agrobacterium, it has been shown that effector loading is essential for secretion, but then why does the pTrc200 secrete Hcp? Also, in Fig. 4B, a strain expressing C1-Tde1(M) now secretes Hcp.

Minor comments:

Fig. 2B could benefit from better labeling to indicate that most strains lack lacY. Also, why is BW25113 WT showing such a low OD420 if it has LacY? Or is WT without lacZ? Please clarify.

2. Significance:

Significance (Required)

It has been known for over a decade that T6SS effectors have both periplasmic and cytosolic targets (e.g., cell wall and DNA). However, it remains unclear (1) where within the target cell are T6SS effectors are delivered and (2) once delivered, how do effectors reach their intracellular target site. In this work, Ali et al. demonstrate that for Tde1, the N-terminal GxxxG motif is essential for Tde1 to reach its target (DNA). The authors identified Tde1 homologs in several bacteria, suggesting that this model may be relevant across a wide range of bacteria. Additional research is needed to (1) determine whether Tde1 is originally secreted into the periplasm and (2) understand how non-Tde1/non-GxxxG effectors reach their target site.

3. How much time do you estimate the authors will need to complete the suggested revisions:

Estimated time to Complete Revisions (Required)

(Decision Recommendation)

Between 1 and 3 months

4. *Review Commons* values the work of reviewers and encourages them to get credit for their work. Select 'Yes' below to register your reviewing activity at Web of Science Reviewer Recognition Service (formerly Publons); note that the content of your review will not be visible on Web of Science.

Web of Science Reviewer Recognition

Yes

Full Revision

Manuscript number: RC-2022-01588
Corresponding author(s): Erh-Min, LAI

[Please use this template only if the submitted manuscript should be considered by the affiliate journal as a full revision in response to the points raised by the reviewers.

*If you wish to submit a preliminary revision with a revision plan, please use our "Revision Plan" template. **It is important to use the appropriate template to clearly inform the editors of your intentions.**]*

1. General Statements [optional]

This section is optional. Insert here any general statements you wish to make about the goal of the study or about the reviews.

The authors thank the reviewers for the positive and valuable comments, which have helped us to improve the quality of this work. We have addressed all comments by providing additional data and/or explanation with a detailed point-by-point response. The revised manuscript included new data: 1) viable cell counts of growth inhibition assay (Fig. 2A), 2) Quantitative data of microscope data (Fig. 2C, Fig. 4), 3) quantitative data of interabacterial competition (Fig. 5A, 5B), western blotting data of growth inhibition (Fig. S1A and S1B), secretion assay of single glycine-zipper mutants (Fig. 5C), and inclusion of full gel of western blot results (Fig. S3 and S5). By integrating these new results, we have substantially strengthened the findings that a glycine zipper motif of a type VI secretion effector T6SS Tde1contributes to its translocation across the cytoplasmic membrane of target cells.

2. Point-by-point description of the revisions

This section is mandatory. Please insert a point-by-point reply describing the revisions that were already carried out and included in the transferred manuscript.

Reviewer #1 (Evidence, reproducibility and clarity (Required)):

Summary:
In this manuscript, Ali et al. propose that a glycine zipper motif located at the N-terminus of the Agrobacterium tumefaciens T6SS DNase effector, Tde1, can transport the toxin across the cytoplasmic membrane and into the cytoplasm, where its target is found. To support these claims, they perform a series of secretion, competition, toxicity, and fluorescence microscopy assays showing that a mutation in two glycine residues affects toxicity of the effector during competition and its ability to enter a target cell, but not its secretion through the T6SS or its binding to the adaptor protein Tap1.
The concept brought forth in this study is quite interesting and important - the notion that T6SS effectors have domains that aid in their transport into the cytoplasm of the target cell. This is similar to a recent finding that a domain

common to bacterial pyocins and T6SS effectors can mediate DNase toxin transport through the target cell's cytoplasmic membrane (Atanaskovic et al., mBio, 2022); the authors should mention and discuss this recent work. Nevertheless, it is my impression that the results do not fully support the conclusions and proposed mechanism, even though the general idea seems correct.

Ans: We thank this reviewer found this work interesting and important. We hope the revised manuscript including the new data and careful interpretation have substantialized the conclusions and proposed mechanisms. We also included the excellent work by Atanaskovic et al., 2022 and discussed the findings in the revision (see lines 344-349).

Major comments:

- An experiment that directly demonstrates the ability of the glycine zipper to mediate transport of a toxin across a membrane would greatly support and solidify the conclusions of this work. For example, showing the ability of a purified protein to enter spheroplasts or liposomes in a glycine zipper dependent fashion. Currently, the authors perform experiments that can only indirectly support the proposed function of the glycine zipper to enable the effector to cross the membrane, and as detailed below, some of these experiments are over-interpreted in my opinion.

Ans: We agree that the direct evidence for the ability of the glycine zipper to mediate Tde1 transport across target cell membrane is to perform the *in vitro* translocation assay. Unfortunately, the attempts to purify sufficeint amounts of full-length or N-termial version of Tde1 have not been successful. Therefore, we are unable to perform this experiment. Accorigly, we have tried our best to carefully interpret the data and rephrase the statements accordingly.

- Lines 153-159: It is not clear how much these results are relevant to the activity of the glycine zipper motif during effector delivery by the T6SS. If I understand correctly, the described experiments are of over-expression of the proteins in the E. coli cytoplasm, where glycine zipper-dependent membrane permeability and toxicity are detected. However, one would expect that if the effector is to be transported from the periplasm to the cytoplasm during T6SS delivery, then the glycine zipper should function from the periplasmic face of the cytoplasmic membrane, and not from its cytoplasmic face, as is the case in these experiments. Is it possible that the observed toxicity and membrane permeability be the result of over-expression in the "wrong place"?

Ans: The reviewer is right that Tde1 should permealize cytoplasmic membrane from periplasmic side upon injection from the attacker based on our proposed model. The purpose of ectopic expression of Tde1 and its variant in *E. coli* is to dissect the region and motif of Tde1 DNase-independent toxicity and the ability in enhancing membrane permeability regardless of which sides of cytoplasmic membrane the Tde1 mediates toxicity and permeability. The results of glycine zipper-dependent toxicity and membrane permeability provide a ground work for the experiments of secretion and interabacterial compeittion in the context of active T6SS action to determine the role of glycine zipper in Tde1 export and translocation.

- Fig. 4B: This figure appears to be very important, and the authors base a large part of their main conclusion regarding the role of the glycine zipper in membrane crossing on it. However, some controls are missing and part of the results observed in the figure do not match their description in the text.

• Lines 233-237 - While the authors state in the text that GFP and mCherry signals did not overlap in E. coli cells co-cultured with Agrobacterium cells expressing Tde1(M)-GLGL, I see many double-colored cells in this sample (bottom panels in Fig. 4B). Actually, all cells appear to have both green and blue colors, except for a few cells that are only green but that also seem to be dead judging by their ghostly appearance in the phase contrast channel.

Ans: We thank the reviewer pointed this out. By looking at this particular image more carefully, it is striking that the majority of cells seem to emit both green and blue colors from this Tde1(M)^{GLGL} sample.

We have performed a total of three indepenent experiments for this translocation assay and all results except this particular sample in this particular experiment are consistent in all three independent experiments. Honestly, we could not explain this result and a possibility is this sample might be accidentally mixed with another sample. Because this is the only sample with inconsistent result with another two independent experiments, we decided NOT to use the results from this independent experiment and instead performed another independent experiment. We now have included the quantitative data from three effective independent experiments and show the representative images in Figure 4.

• How is it that all cells in the bottom panels are blue (indicating that they are E. coli target cells)? Shouldn't a large portion of the cells be Agrobacterium cells that should not be blue, since these are added at the beginning of the competition assay at a 10:1 ratio in their favor?

Ans: As explained above, we have no defined answer and decided to perform additional repeats, which are consistent with results of another two independent experiments.

• It is quite remarkable that so much GFP signal is transported into the E. coli target cells so that it is so clearly visible under the microscope. How do the authors know that the GFP signal overlapping with the mCherry is really inside the cell and not outside (for example, proteins secreted to the media that attach to the cell envelope)? Will the GFP signal remain if trypsin is added to the media before visualization under the microscope?

Ans: Indeed, our quantitative data show there are ~50% cells have GFP overlapping with mCherry in the translocation positive samples. The signals should be inside the cells because no overlay signals were observed from N-Tde1^{GLGL} or Tde1(M)^{GLGL} even though they are secreted.

• Can the authors quantify the ratio of E. coli cells that have overlapping green and blue colors over several experiments for each sample, to show that this phenomenon repeats and is statistically significant?

Ans: Yes, see quantitative data in Figure 4.

• Can the authors explain why at least some of these E. coli cells should not be dead due to the toxicity mediated by the third effector of the Agrobacterium T6SS, Tae?

Ans: In *Agrobacterium tumefaciens* C58, Tde1/2 are the major effectors contributing to antibacterial activity. Tae effector has little impact on interbacterial competition outcome (see previous publications Ma et al., 2014 doi: 10.1016/j.chom.2014.06.002.; Yu et al., 2020 doi.org/10.1128/JB.00490-20)

• Why were the microscopy competitions performed differently than the regular competition assays? Why wasn't AK media used in these competitions? How active is the T6SS under these conditions compared to the AK media?

Ans: We have tried to use AK medium for the translocation assay but only very weak fluorescent signals can be observed likely due to the low expression when grown on this nutrient poor medium. In order to correlate the results of the compeittion assay with translcoation experiment, we have performed *E. coli* killing assay using LB medium that is used for translocation experiment now. For the interbacterial competition against agrobacterial siblings, we still used AK medium for competition because no detectable interbacterial compettion activity could be observed between two *A. tumefaciens* strains on LB agar. As reported earlier, stronger interbacterial competition outcome was detected from co-culture on AK than other nutrient rich medium while the secretion activity grown in AK medium is lower (Yu et al., 2020 doi.org/10.1128/JB.00490-20). These results indicate that the factors other than secretion activity also impacted recipient cell susceptibity, which however is not the main focous of this work.

• In the N-Tde1 sample, many Agrobacterium cells appear to have the GFP signal in foci rather than distributed throughout the cell (as it is in other samples), while the E. coli cells have a uniform and strong GFP signal. Can the authors comment on that?

Ans: Thanks the reviewer for raising this question.We are also curious about the Tde1 glycine zipper-dependent GFP foci and now include this potential explanation in the Discussion of revised manuscript (line 387-406). To this end, we do not have an answer for it. Because glycine zipper repeats are known to interact with membrane, it is possible that Tde1 proteins may preferntially bind to microdomain of cytoplasmic membrane, which was recently found in *A. tumefaciens* (Czolkoss et al., 2021). We also found that Tde1 proteins (either tagged with HA or GFP) are proned for truncation when they are ectopically expressed in *E. coli* or when Tdi1 is absent or not equivalent. Thus, it is possible that Tde1-GFP proteins are truncated after translocation into *E. coli* cells, in which most GFP signals are emitted from free GFP instead of Tde1-GFP. The stability of free GFP derived from translocated Tde1-GFP may also explain the high percentage of *E. coli* cells exhibiting overlayed GFP/mCherry signals.

• It might be easier for readers to visualize this figure and see the signal distribution in the different cells if the authors show a zoomed in version in the main text, and provide the wide field images as a supplementary figure.

Ans: We have tried to include zoom-in images but the resolution is not good. We have improved the quality of images in the Figure 4 and believe the images are clear to see individual and overlayed fluorescence signals.

- Fig. 5C-D: The reduced expression and secretion of the GLGL mutant is considerable. How can the authors rule out that this reduction was the cause for the reduced observed toxicity of the mutant in 5A-B? Moreover, the results show that the GLGL double mutant is hampered in expression, secretion, and DNase activity, and it negatively affects overall T6SS activity. Since this mutant was used throughout the paper, and in the absence of a direct assay showing membrane transport mediated by the glycine zipper motif, the claim of the role of this motif in membrane crossing is not well substantiated by the results. If the authors were to show that the single glycine mutants used in Fig. 5D, which are stable and have an intact DNase activity, behave as claimed in the final conclusion sentence (lines 279-283), then the conclusions will be better substantiated by the results.

Ans: Thank you very much for suggesting this important experiment. We have now constructed the single G39L and G43L variants expressed together with Tdi1 in *A. tumefaciens tdei* mutant for both secretion and interbacterial competition assays (see description in lines 259-280 and Fig. 5). As shown in Figure 5, both G39L and G43L variants are expressed and secreted at similar or even higher levels than wild type Tde1 but have no detectable antibacterial activity against either *E. coli* or *A. tumefaciens* 1D1609. This result substantializes the role of this glycine zipper motif in translocation.

Minor comments:

- Line 93: I am not sure that Ntox15 should still be referred to as a "novel" domain.

Ans: despite the evidence of this domain as DNase, the name of Ntox15 is used. We think to keep this nomenclatture as it will be easier to be ditinquished from other nuclease or toxin domain.

- Line 105: The section's heading does not actually describe its content. The results here only show toxicity upon over-expression of the effector or its mutant forms in E. coli. Therefore, this cannot be referred to as a "prey cell" since the effector was not transported into it during competition. Moreover, the results in Fig. 5A do not support

DNase-independent toxicity during competition.

Ans: The heading is changed to "Tde1 exhibits DNase-independent growth inhibition in *E. coli*" (line 115).

- Please consider making all of the symbols in the growth assays semi-transparent. It is impossible to discern between the different, overlapping curves.

Ans: The growth curve results are improved by changing line colors and reducing size bars (Fig. 1B, 1C; Fig. 2A, 2D)

- Please consider making the size bars in all microscopy images more pronounced. They are barely visible in their current form. Also, it would be better to show images of the same magnification/zoom for the different samples, since the current presentation shows cells from different samples at different sizes, and it can be confusing to the readers.

Ans: Amended (Fig. 2C; Fig. 4).

- In Fig. 1B and in Fig. 2A the authors show that expression of Tde1(M) in cells is toxic, yet in Fig. 2D they see no toxicity. Can the authors please comment on this discrepancy?

Ans: Fig. 2D showed the viability of *E. coli* cells after Tde1 variants were induced for 1 hr before ONPG uptake assay to indicate the increased membrane permeability is not due to cell death. In Fig. 1B, the growth inhibition of Tde1(M) is also not evident at 1 hr. So, the results are consistent.

- I am not convinced that the assay in Fig. 2E can be used to determine bacteriostatic/bacteriolytic effect. It is not clear how such a distinction can be made from OD measurements, since an increase in OD can result from the entire population growing after removal of the stressor, or just part of the population that did not lyse/die. To make such a claim, the authors can spot bacteria on repressing media at different timepoints after protein induction, and then determine CFU.

Ans: The increased OD_{600} value during recovery could be caused by either resumed cell division or cell elongation. Based on the newly added growth inhibition assay of all Tde1 variants which we showed nice correlation between CFU counting and OD_{600} value (Fig. 2A, S2) and no increased cell size/length of *E. coli* cells expressing N-Tde1 or Tde1(M), we think the recovered OD_{600} value is supportive of N-Tde1 or Tde1(M) exhibiting bacteriostatic toxicity. In addition to that, our interbacterial competition data showed that Tde1(M)-Tdi1 which is still having intact glycine zipper doesn't show significant detectable killing, supporting the bacteriostatic function of Tde1 glycine zippers. In fact, we performed this experiment based on Mariano et al.(Nat. Commun. 2019 doi: 10.1038/s41467-019-13439-0), which showed the recovery of OD_{600} value after removal of inducer as the evidence that the Ssp6 toxin is not bacteriolytic.

- Fig. 3A: A control is missing. To verify that the N-terminal part of Tde1 is not promiscuously interacting with proteins, the authors should include a control sample testing its inability to precipitate a protein other than Tap1 in the same experiment.

Ans: Our previous study has showed that Tde1 can co-immunprecipiate Tap1 but not a non-T6SS protein RpoA (Bondage et al., 2016 doi:10.1073/pnas.1600428113), indicating that Tde1 is not promiscuously interacting with proteins. Considering the tight biochemical interaction between Tap1 with N-Tde1 but not C-Tde1 that correlate with their ability for secretion upon loading onto VgrG1, N-Tde1 is unlikely to bind proteins non-specifically. This is also supported by the non-specific protein bands from cellular fractions recognized by anti-Tap1 are not co-immunoprecipitated by any of Tde1 variants (Fig. S3). We could repeat the experiments to include additional proteins as negative controls but we chose to use time for other more critical experiments during the limited revision time.

- Fig. 3B: the blots are very "dirty". It is not clear how the authors were able to determine expression and precipitation of some truncations (for example, C2-Tde1 in the E. coli IP panel looks like a background band found in other lanes too).

Ans: We agree that western blots of co-IP experiments in *E. coli* are not very clear due to the weak signals of some Tde1 variants and background. As pointed out by the reviewer 3, this result is not conclusive and rovide little additional information other than the co-IP results from *A. tumefaciens.* Because the interaction between Tde1 variants and Tap1 when expressed in *E. coli* are not physiologically relevant and not the main focus of this work, we have removed the *E. coli* co-IP results from this manuscript as suggested by the reviewer 3.

- Lines 222-225 (Fig. 4A): I can't see C-1-Tde1(M)-sfGFP in the cellular blot. All the bands in this lane look like background bands that are also present in all other lanes. Therefore, I am not sure how the conclusion regarding this truncation's ability to be secreted was reached.

Ans: We agree that C1-Tde1(M)-sfGFP is barely detectable due to its weak signal overlapping with cross-reacted bands. Since several attemps to improve the western blot quality by changing antibody and pre-blocking with protein lysages of vector control strain did not produce convincing results for detection of C1-Tde1(M)-sfGFP, we have rephrased the description of this result as "However, C1-Tde1(M)-sfGFP protein signal could not be unambiguously determined in the cellular fraction due to the overlapping of its predicted protein band with cross-reacted proteins, and no corresponding C1-Tde1(M)-sfGFP band was detected in the extracellular fraction." (line 234-237).

- Fig. 4A: the protein names above the lanes should include the sfGFP that is fused to them.

Ans: Amended.

- It would be preferable to show quantitative competition assays with statistics rather than pictures of a plate showing a single competition result, if conclusions or observations on minor differences in toxicity are made (for example, line 253: "The killing activity of Δtdei(Tde1GLGL-Tdi1) was largely compromised"). Since the authors performed each competition assay more than once, these data should be available to them.

Ans: Amended. We have repeated the interbacterial competition experiments including single G39L and G43L variants for multiple biological repeats (see detailed in legends of Fig. 5A, 5B). The quantitative data with statistical analysis were added, which show no statistical difference of any glycine zipper mutants as comapred to Tde1(M) or when expressed in the T6SS mutant. Thus, there are no detectable antibacterial activity of glycine zipper mutants against either *E. coli* or *A. tumefaciens* siblings.

- Fig. 5A: The author claim at the beginning of the manuscript (first results section heading: "Tde1 can cause DNase-independent growth inhibition of prey cells") that the N-terminal region of Tde1 is toxic on its own in the prey cell, yet in this competition assay Tdi1(M) shows no toxicity against the E. coli target cells. In the microscopy assay (Fig. 4B), it appears that a lot of Tdi1(M) enters the prey cell, since we can visualize it under the microscope. Can the authors clarify this discrepancy and explain why they do not expect to see target killing by this mutant even though they claimed it is toxic earlier?

Ans: As describbed in earlier response, N-Tde1 amd Tde1(M) toxicity can exhibit toxicity by ectopic expression in *E. coli.* We mainly used this ectopic expression assay to dissect the region and motif contributing the toxicity. Compared to the interbacterial competiton process where Tde1(M) may only transiently permealze cytoplasmic membrane transiently as the final destination is cytoplasm where wild

type Tde1 but not Tde1(M) exerts DNase toxicity. Thus, the toxicity of N-Tde1 and Tde1(M) can be only observed when the proteins are continuously produced in the cytoplasm. The role of N-Tde1, specifically the glycine zipper motifs, is to mediate Tde1 translocation across inner membrane, instead of exerting toxicity during the context of interbacterial competition.

- Fig. 5B: the GLGL mutant seems to have some residual toxicity, not dissimilar to what is shown in 5A. Why are these similar results interpreted differently (in 5A they are "largely compromised", while in 5B "killing activity... was not detectable")? Also, why was Tde(M)1-Tdi1 used in Fig. 5A but Tdi1(M) without the immunity gene used in Fig. 5B?
Ans: As described above, to better quantify the interbacterial competition outcomes, we have repeated the interbacterial competition experiments and used Tde(M)1-Tdi1 instead of Tdi1(M) for at least three biological replicates. The quantitative data with statistical analysis were carried out to clarify this ambiguity (Fig. 5A, 5B).

- Fig. 5: Does the remaining third effector, Tae, not play a role in these competition assays? If, as shown in Fig. 5C, the entire T6SS is less active when a GLGL mutant is expressed, couldn't the different in toxicity shown in Figs. 5A-B be the result of lack of Tae secretion and toxicity?
Ans: As decribed above, Tae effector has little impact on interbacterial competition outcome. The quantatitive interbacterial competition results (Fig. 5A, 5B) also clarify the ambiguity because single G39L and G43L variants are expressed and secreted at similar or even higher levels than wild type Tde1 but have no detectable antibacterial activity against either *E. coli* or *A. tumefaciens* 1D1609.

- Lines 359-362: T6SS effectors that bind the inner Hcp tube were suggested to be only partially folded.
Ans: Amended.

Reviewer #1 (Significance (Required)):

The concept of T6SS effectors providing their own mechanism of transport from the cytoplasm to the periplasm is very interesting. It will appeal to audience in a wide range of microbiology disciplines, including those interested in toxins, membrane transport, and even translational applications. A similar concept was recently proposed and demonstrated for a domain that is also found in T6SS effectors (Atanaskovic et al., mBio, 2022).

Expertise: I have been studying the different aspects of T6SS for the past decade.

Reviewer #2 (Evidence, reproducibility and clarity (Required)):

This manuscript is focused on understanding how the Agrobacterium tumefaciens T6SS effector, Tde1, is translocated across the cell envelope of target cells and how this effector binds to the adapter Tap1. The authors show that GxxxG motifs in the N terminal region of Tde1 are required for delivery into the cytoplasm of target cells and permeabilising the cytoplasmic membrane. Given that these GxxxG motifs resemble glycine zipper structures that are found in proteins involved in membrane channel formation, the authors propose that these Tde1 motifs are involved in channel formation in the target cell. The authors also show that the N terminal region of Tde1 binds to Tap1 to facilitate loading onto the T6SS machinery but that the GxxxG motifs are not involved in this binding. Overall the manuscript was easy to read and followed a logical presentation of the findings. There are a few major comments that this reviewer has below - addressing these would allow the authors' claims to be more robustly supported.
Ans: Thank you very much for the positive comments and valuable suggestion. We hope the revised

manuscript including the new data and careful interpretation have substantialized the conclusions and proposed mechanisms.

Major comments:

1. Fig 1B: Why is this such a short growth experiment (5 hrs total with 2 hr pre and 3 hrs post induction)? Reporting on a growth experiment would normally be at least until the cells reach stationary phase but here the cells are still clearly in exponential phase. This reviewer would query what happens to growth rate in later exponential growth and into stationary phase? Is the toxic effect lessened in later stages of growth?
Ans: We have indeed performed the growth curve analysis with longer time period. However, we noted that the growth at later time points are not always consistent and our interpretation is that the continuous expression of toxins may lead to the selection of mutants. Since the 3 or 4 hr time period already showed the toxicity phenotype, we have focused on this time frame for the growh experiments.

2. It is indeed surprising that C2-Tde1(WT) does not inhibit growth despite it having a functional DNase domain and being expressed in the cytoplasm. Did the authors confirm that this protein variant was expressed by Western blot or other means? This should be done to confirm that this variant is indeed not impacting upon growth instead of it not impacting growth simply because it is not being expressed.
Ans: Amended. All Tde1 variants including C2-Tde1 are expressed (data included in Fig S1)

3. The letters used to report significance are not clear to this reviewer. The authors say that "The significant differences were shown by the different letters (p value <0.01)" and then have a, b, c etc next to lines of the growth experiments in Figure 1B, C and Fig 2A, B, E etc. Which comparisons have a p value <0.01? this is not clear.
Ans: Sorry for the confusion. All statistics carried out in this mansucript are one way ANOVA followed by the Tukey's multiple comparison or Fishers Least Significant Difference (LSD) test as described in the Materials and Methods. We also rephrased the description as " Different letters indicate statistically different groups of strains" in figure legend for clarity (Fig. 1B; 2C, 2D; 5A, 5B, 5E; S1D).

4. For all fluorescence microscopy experiments how many fields of view were imaged for each biological replicate? Were the fields selected at random or was the field selection biased to what was present in the field before taking the image? The answers to all of these questions should be stated in the methods. Also the microscopy data presented in the manuscript is not quantitative. Quantification of the number of cells with PI vs Hoechst signal (in Fig 2C) and mcherry vs gfp signal (in Fig 4B) for all fields of view and for all biological replicates would be very informative and convince the reader that the authors have not just "cherry picked" the images they are showing in the manuscript. This could be performed manually or the authors could use the freely available image analysis program Fiji (https://imagej.net/software/fiji/) to perform these analysis in a semi-automated manner.
Ans: The number of images and experiments were now described in the figure legends and the quantititive data are included (Fig. 2C).

5. For the co-IP experiments in Fig 3 where interaction between HA tagged Tde1 and Tap1 is demonstrated the authors should also show that Tap1 does not interact with a different HA-tagged protein i.e. that the interaction is specific to Tde1 and not the HA motif.

Review COMMONS

Ans: All Tde1 variants were tagged with HA. As shown in Fig. 3A, Tap1was not co-precipitated by C2-Tde1 and C1-Tde1(M), indicating that Tap1 specifically interacts with N-terminal region of Tde1.

6. For all Western blot images there should be at least 2 protein standard markers present in each individual blot - i.e. for Fig 3A and B the bottom panel showing Tap1 detection only has the 35 kDa marker, it should have at least one more marker in it. The same is true for other panels in Fig 2, 3 and 4. Having at least two molecular weight markers in a panel is now standard for most journals when presenting Western blot images.
Ans: Amended. We have now included the full gel of western blot results in Fig. S3 and S5 of those shown in main figures.

7. For the competition assay serial dilution images in Fig 5A-B the images are a nice way to visually represent the experimental outcome but they should accompany graphs showing the competitive index of CFU/ml of the input prey and attacker vs the output prey and attacker for all biological replicates. This will convince the reader that the authors had equivalent amounts of the prey and the attacker going into the experiment and also that all attackers grew at the same rate and so were equally able to target the prey cell. This quantification could also provide more convincing out competition of ID1609 prey by C58 attacker (Fig 5B).
Ans: Amended. As indicated above, we have repeated the interbacterial competition experiments for at leaset three biological replicates and show that quantitative data with statistical analysis (Fig. 5A, 5B).

Minor comments:

Line 40: should read "...demonstrate that the effector itself..."
Ans: The sentence has been rephrased (line 40) .

Line 41: "...we propose..." instead of "...we proposed..." since present tense makes more sense for this statement.
Ans: Amended (line 42).

Line 51: "Each specialized protein secretion system" instead of "Each of...."
Ans: Amended (line 52).

Line 76: "A glycine zipper structure..."
Ans: Amended (line 83).

Line 79: "For example..."
Ans: Amended (line 86).

Lines 96-100: The present tense should be used here as the current usage of past tense implies that this has been done in previous work and not in the current study - eg "we revealed", "we showed" would be better as "we reveal", "we show".
Ans: Thanks for the advice. We have made changes throughout the manuscript.

Fig 5B - The competition assay serial dilution images look a bit blurry, are there images the authors could use that are not blurry?
Ans: Amended. As indicated above, we now show quantitative data with statistical analysis (Fig. 5A, 5B).

Reviewer #2 (Significance (Required)):

This work is significant in as while there is a great deal known about how T6SS effectors cause toxicity there is less known about how these effectors are loaded onto the T6SS machinery and very little known about how T6SS effectors are able to translocate across the cytoplasmic membrane of target cells to reach a cellular component that is in the cytoplasm. This work would be of wide general interest to researchers in the T6SS field as well as those interested in bacterial secretion systems.

Reviewer expertise key words: Molecular microbiology, T6SS, interbacterial competition

Reviewer #3 (Evidence, reproducibility and clarity (Required)):

EVIDENCE, REPRODUCIBILITY AND CLARITY
=====================================

Summary:

In this work, Ali et al. demonstrate that the N-terminal GxxxG motif of the T6SS DNase effector Tde1 of Agrobacterium tumefaciens is required for interbacterial intoxication. Using a combination of cell viability, reporter, and microscopy assays, the authors demonstrate that over-expression of the N-terminus of Tde1 results in inner membrane permeability. Moreover, the authors show that both the interaction between Tde1 and its adaptor Tap1 as well as the T6SS-mediated secretion of Tde1 are dependent on the N-terminus of Tde1. Finally, using a combination of in vitro and in vivo experiments, the authors determine that the N-terminal GxxxG motif is essential to Tde1-dependent interbacterial killing by enabling effector entry into competing bacterial cells.

Major comments:

If N-tde1 is 1-97 aa, the predicted size is 9 kDa, but it shows up as ~17 kDa? Can the authors comment on this? Does N-tde1 or tde1 dimerize?

Ans: The theoretical Mw of N-Tde1-HA is 10.64 KDa, which indeed migrated at higer position ~17 kDa. It is notable that full-length Tde1 with theoretical 29.5-kDa migrated slower in SDS-PAGE with a observed size ~36 kDa as observed previously (Ma et al., 2014 doi:10.1016/j.chom.2014.06.002). Similarly, the full-length HA-tagged Tde1(M) with theoretical 30.89 kDa migrated at a position ~38 kDa. Since the protein samples analyzed by SDS-PAGE including reducing agent, we cannot exclude the possibility that Tde1 or N-Tde1 may form dimer or oligomer that was disrupted by SDS-PAGE but it appears not forming dimer on SDS-PAGE.

I have many concerns with the data and conclusions drawn from the data in Fig. 3B. I recommend removing it since (1) the data are not accurately represented in the text and (2) it is difficult to ascertain whether biologically relevant conclusions can be drawn from what happens with Agrobacterium proteins in E. coli. Below is a summary of my concerns regarding this section: I disagree with the authors' statements in lines 191-198. Their pulldown with E. coli is not consistent with their pulldown in C58. In fact, given the expression problems of some of the constructs in E. coli, I believe the data shown in Fig. 3B is inconclusive. The amount of Tap1 that co-IP'ed with N-Tde1GLGL and Tde1(M) is very low even though the expression levels of N-Tde1GLGL and Tde1(M) were relatively strong. Therefore, I do not feel confident concluding that these proteins "interact". Secondly, Tde1(M)GLGL was not expressed in E. coli, so no conclusions can be drawn. Moreover, the C1 and C2 variants were also not expressed well, so I believe the authors' statement in line 191-192: "Similar to the results in A. tumefaciens, the N-Tde1 and Tde1(M) interacted with Tap1 but not the C-terminal variants", is unjustified. You cannot rule out that C1 and C2 do not interact with Tap1 because C1

and C2, like Tde1(M)GLGL, were not expressed well in E. coli.

Ans: We agree with the reviewer that the *E. coli* co-IP result is not conclusive due to the low expression and instability of proteins mostly during the process of cell lysis and purification, and it provides little additional information other than data from co-IP in *A. tumefaciens.* Because the interaction between Tde1 variants and Tap1 when expressed in *E. coli* are not physiologically relevant and not the main focus of this work, we have removed the *E. coli* co-IP results from this manuscript.

Lines 211-214: It looks like C1-Tde1(M) inhibits T6SS secretion. I am aware that in Agrobacterium, it has been shown that effector loading is essential for secretion, but then why does the pTrc200 secrete Hcp? Also, in Fig. 4B, a strain expressing C1-Tde1(M) now secretes Hcp.

Ans: Thanks for noting our previous finding that Tde loading is critical for secretion. Our data are indeed supportive of the effector loading in activating T6SS as only very low levels of Hcp secretion could be detected from the strain containing vector only or C1-Tde1(M). In our previous paper (Wu et al., 2020 https://doi.org/10.15252/embr.201947961), there is either little or no detection of Hcp secretions when effectors are not loaded, indicating that effector loading is important but not essential for Hcp secretion. Because overexpression of VgrG can also activate T6SS secretion in the absence of effector loading (Bondage et al., 2016 doi:10.1073/pnas.1600428113), we think the low level secretion under certain conditions could be caused by some cells with higher levels of VgrG protein concentration but more work is required to elucidate the underlying mechanisms.

Minor comments:

Fig. 2B could benefit from better labeling to indicate that most strains lack lacY. Also, why is BW25113 WT showing such a low OD420 if it has LacY? Or is WT without lacZ? Please clarify.

Ans: We apologize for not labeling clearly. The BW25113 strain lacks *lacZ*, therefore all the Δ*lacY* strains were complemented with a plasmid encoding *lacZ* (pYTA-lacZ). We have now added the labels to avoid confusion (Fig. 2B).

Reviewer #3 (Significance (Required)):

SIGNIFICANCE
============

It has been known for over a decade that T6SS effectors have both periplasmic and cytosolic targets (e.g., cell wall and DNA). However, it remains unclear (1) where within the target cell are T6SS effectors are delivered and (2) once delivered, how do effectors reach their intracellular target site. In this work, Ali et al. demonstrate that for Tde1, the N-terminal GxxxG motif is essential for Tde1 to reach its target (DNA). The authors identified Tde1 homologs in several bacteria, suggesting that this model may be relevant across a wide range of bacteria. Additional research is needed to (1) determine whether Tde1 is originally secreted into the periplasm and (2) understand how non-Tde1/non-GxxxG effectors reach their target site.

1st Editorial Decision **7th Feb 2023**

Dear Dr. Lai,

Thank you for transferring your revised manuscript from Review Commons to EMBO reports. I have now received the reports from three of the two referees that had assessed your study at Review Commons that were asked to re-evaluate the manuscript. Original referee #3 declined my invitation to re-assess the study. However, going through your p-b-p-response, I consider his/her concerns as adequately addressed. Please find the comments of the remaining two referees below. As you will see, the referees now support the publication of your study. Nevertheless, they have remaining concerns and suggestions to improve the study, I ask you to address in a final revised manuscript.

Moreover, the manuscript needs formatting according to our journal style. Please contact me to discuss the revision (also by video chat) if you have questions or comments.

Please carefully review the instructions that follow below.

PLEASE NOTE THAT upon resubmission revised manuscripts are subjected to an initial quality control prior to exposition to re-review. Upon failure in the initial quality control, the manuscripts are sent back to the authors, which may lead to delays. Frequent reasons for such a failure are the lack of the data availability section (please see below) and the presence of statistics based on n=2 (the authors are then asked to present scatter plots or provide more data points).

When submitting your final revised manuscript, we will require:

1) a .docx formatted version of the final manuscript text (including legends for main figures, EV figures and tables), but without the figures included. Figure legends should be compiled at the end of the manuscript text.

2) individual production quality figure files as .eps, .tif, .jpg (one file per figure), of main figures and EV figures. Please upload these as separate, individual files upon re-submission.

The Expanded View format, which will be displayed in the main HTML of the paper in a collapsible format, has replaced the Supplementary information. You can submit up to 5 images as Expanded View. Please follow the nomenclature Figure EV1, Figure EV2 etc. The figure legend for these should be included in the main manuscript document file in a section called Expanded View Figure Legends after the main Figure Legends section. Additional Supplementary material should be supplied as a single pdf file labeled Appendix. The Appendix should have page numbers and needs to include a table of content on the first page (with page numbers) and legends for all content. Please follow the nomenclature Appendix Figure Sx, Appendix Table Sx etc. throughout the text, and also label the figures and tables according to this nomenclature.

For more details, please refer to our guide to authors:
http://www.embopress.org/page/journal/14693178/authorguide#manuscriptpreparation

Please consult our guide for figure preparation:
http://wol-prod-cdn.literatumonline.com/pb-assets/embo-site/EMBOPress_Figure_Guidelines_061115-1561436025777.pdf

See also the guidelines for figure legend preparation:
https://www.embopress.org/page/journal/14693178/authorguide#figureformat

3) a complete author checklist, which you can download from our author guidelines (https://www.embopress.org/page/journal/14693178/authorguide). Please insert page numbers in the checklist to indicate where the requested information can be found in the manuscript. The completed author checklist will also be part of the RPF.

Please also follow our guidelines for the use of living organisms, and the respective reporting guidelines:
http://www.embopress.org/page/journal/14693178/authorguide#livingorganisms

4) that primary datasets produced in this study (e.g. RNA-seq, ChIP-seq, structural and array data) are deposited in an appropriate public database. If no primary datasets have been deposited, please also state this in a dedicated section (e.g. 'No primary datasets have been generated and deposited'), see below.

See also: http://embor.embopress.org/authorguide#datadeposition

Please remember to provide a reviewer password if the datasets are not yet public.

The accession numbers and database should be listed in a formal "Data Availability" section (placed after Materials & Methods) that follows the model below. This is now mandatory (like the COI statement). Please note that the Data Availability Section is restricted to new primary data that are part of this study. This section is mandatory. As indicated above, if no primary datasets

have been deposited, please state this in this section

Data availability

The datasets produced in this study are available in the following databases:

- RNA-Seq data: Gene Expression Omnibus GSE46843 (https://www.ncbi.nlm.nih.gov/geo/query/acc.cgi?acc=GSE46843)
- [data type]: [name of the resource] [accession number/identifier/doi] ([URL or identifiers.org/DATABASE:ACCESSION])

*** Note - All links should resolve to a page where the data can be accessed. ***

Moreover, I have these editorial requests:

6) We now request the publication of original source data with the aim of making primary data more accessible and transparent to the reader. Our source data coordinator (in cc) will contact you to discuss which figure panels we would need source data for and will also provide you with helpful tips on how to upload and organize the files.

7) Our journal encourages inclusion of *data citations in the reference list* to directly cite datasets that were re-used and obtained from public databases. Data citations in the article text are distinct from normal bibliographical citations and should directly link to the database records from which the data can be accessed. In the main text, data citations are formatted as follows: "Data ref: Smith et al, 2001" or "Data ref: NCBI Sequence Read Archive PRJNA342805, 2017". In the Reference list, data citations must be labeled with "[DATASET]". A data reference must provide the database name, accession number/identifiers and a resolvable link to the landing page from which the data can be accessed at the end of the reference. Further instructions are available at: http://www.embopress.org/page/journal/14693178/authorguide#referencesformat

8) Regarding data quantification and statistics, please make sure that the number "n" for how many independent experiments were performed, their nature (biological versus technical replicates), the bars and error bars (e.g. SEM, SD) and the test used to calculate p-values is indicated in the respective figure legends (also for potential EV figures and all those in the final Appendix). Please also check that all the p-values are explained in the legend, and that these fit to those shown in the figure. Please provide statistical testing where applicable. Please add complete statistical testing to all diagrams (for main, EV figures). Please also indicate (e.g. with n.s.) if testing was performed, but the differences are not significant. Please avoid the phrase 'independent experiment', but clearly state if these were biological or technical replicates. In case n=2, please show the data as separate datapoints without error bars and statistics. See also:
http://www.embopress.org/page/journal/14693178/authorguide#statisticalanalysis

If n<5, please show single datapoints for diagrams.

Please see also the comments by referee #2 regarding statistics.

9) Please also note our reference format:
http://www.embopress.org/page/journal/14693178/authorguide#referencesformat

10) For all microscopic images, please add scale bars of similar style and thickness to all the microscopic images, using clearly visible black or white bars (depending on the background). Please place these in the lower right corner of the images. Please do not write on or near the bars in the image but define the size in the respective figure legend.

11) We updated our journal's competing interests policy in January 2022 and request authors to consider both actual and perceived competing interests. Please review the policy https://www.embopress.org/competing-interests and update your competing interests if necessary. Please name this section 'Disclosure and Competing Interests Statement' and put it after the Acknowledgements section.

12) Please make sure that all the funding information is entered into the online submission system and is complete and similar to the one in the acknowledgements section of the manuscript text file.

13) We now use CRediT to specify the contributions of each author in the journal submission system. CRediT replaces the author contribution section. Please use the free text box to provide more detailed descriptions and remove the author contributions from the manuscript text file. See also guide to authors:
https://www.embopress.org/page/journal/14693178/authorguide#authorshipguidelines

14) Please provide a title with not more than 100 characters (including spaces).

15) Please make sure that the manuscript sections are ordered like this:
Title page - Abstract - Introduction - Results - Discussion - Materials and Methods - Data availability section (DAS) - Acknowledgements - Disclosure and Competing Interests Statement - References - Figure legends - Expanded View Figure

legends

16) I would suggest to upload the information provided in the supplementary tables as a 'Reagents and Tools table' (and to remove the tables from the manuscript text). I have attached templates for the table in word or excel format. Please upload the filled in table to the manuscript tracking system as a 'Reagent Table' file. Please also provide callouts to this table. The example linked below shows how the table will display in the published article and includes examples of the type of information that should be provided for the different categories of reagents and tools. Please list your reagents/tools using the categories provided in the template and do not add additional subheadings to the table. Reagents/tools that do not fit in any of the specific categories can be listed under "Other":
https://www.embopress.org/pb%2Dassets/embo-site/msb_177951_sample_FINAL.pdf

18) Thanks for providing the source data for the Western blots as supplementary figures. Please provide these as source data files, one PDF file per figure, and combine these with the data requested by our source data coordinator. Please combine all files for one figure in one folder and upload this ZIPed together.

In addition, I would need from you:
- a short, two-sentence summary of the manuscript (not more than 35 words).
- three to four short one sentence bullet points highlighting the key findings of your study.
- a schematic summary figure (synopsis image) in jpeg or tiff format with the exact width of 550 pixels and a height of not more than 400 pixels that can be used as a visual synopsis on our website.

I look forward to seeing a revised version of your manuscript when it is ready. Please let me know if you have questions or comments regarding the revision.

Please use this link to submit your revision: https://embor.msubmit.net/cgi-bin/main.plex

Best,

Achim Breiling
Senior Editor
EMBO Reports

Referee #1:

This is a revised version of a manuscript submitted by Ali et al. via the Review Commons platform. The authors addressed many of my concerns regarding the original manuscript. However, a few minor concerns remain, and a few additional concerns arose when reading the newly added data.

1. Line 115 - in reference to my comment from the previous round, I meant that the word "novel" to describe a domain published 10 years ago may be inappropriate.

2. (A) Line 142 - I am a bit confused by the citation of Fig. S2 here. Panel A shows results for homologs containing Ntox15, not the N-terminus of Tde1, so I assume panel B is the one that should be cited. Panel B shows two types of operons containing a glycine zipper, but it is unclear to what end the same operon from three agrobacterium strains is shown. Also, not all colors are explained in the legend, making this panel challenging to understand.
(B) Note that the blue rectangle in the middle Tde1 gene in panel B lacks a black frame, and the figure caption for panel A mentions that the numbers are shown on the left, yet they are shown on the right side of the figure.
(C) "FIX-1" domains should be renamed "FIX" of "FIX-like" to match the annotation on NCBI.
(D) Overall, the figure will benefit from text and shape alignment, and from keeping the fonts similar.
(E) Why is Fig. S2 cited before Fig. S1C-F? Perhaps the order of supplementary figures should be reconsidered.

3. Fig. S1B - please explain what is the "loading control" in the figure caption.

4. Line 169-170 - there is a noticeable difference of ~2-fold in the number of viable cells (CFU/ml) between the strains in Fig. 2A (with a very small SD). This contradicts the result in the single plate image provided in Fig. S1E. Can the authors explain the discrepancy between the results and why they describe one over the other in the text?

5. Fig. 2B - (A) Isn't the LacZ plasmid present in all strains, or at least also in the lacY mutant as described in the text? If so, please extend the line at the bottom of the graph to clarify this.
(B) Is "LacY" the lacY mutant? If so, please clarify this in the figure (X-axis) or in the caption.

6. Fig. 2C - This is not exactly my area of expertise, but wouldn't one expect that two dyes that bind DNA (Hoechst and PI) will at least somewhat overlap? I find it curious that the Hoechst signals mostly show nucleoid structures (indicating that the cells are

probably stressed) while the PI signals are uniformly distributed throughout the cell. Can the authors please comment on this?

7. Line 187 - should "bacteriolytic" be changed to "bactericidal"?

8. Fig. 3A - in reference to my comment from the previous round, I am not persuaded by the authors' reply. In co-IP experiments, controls must be performed as part of the same experiment. A control sample cannot come from a separate experiment. At least show for N-Tde1 that its interaction with Tap-1 is specific.

9. Fig. 4 - the legend on the right side of the graph is redundant with the X-axis titles.

10. Line 263 and 264 - should "Tdei1" be "Tdi1"?

11. Fig. 5C - why wasn't secretion from the tssK mutant tested for the single glycine mutants (bottom panel) to show that their secretion is dependent on T6SS?

12. Fig. S5B - Should the bottom rectangles on the gels be marked as Hcp?

13. Line 320 - if Fig. S2 (I assume this refers to panel A) is cited only here for the first time, perhaps it should not be part of Fig. S2?

Referee #2:

I thank the authors for the work they have done to revise the manuscript. I think that it is significantly improved and there is more support for the conclusions that they draw from their data. I am happy with all of the changes they made to the manuscript in response to my comments in my last review and believe they have adequately addressed all but one of my comments. The outstanding issue I have is how they explain the outcomes of their statistical analyses in their figures. The authors say that "We also rephrased the description as " Different letters indicate statistically different groups of strains" in figure legend for clarity (Fig. 1B; 2C, 2D; 5A, 5B, 5E; S1D)". This is fine but they do not say what the different letters refer to other than the p value being <0.01. Is 'a' referring to a p value <X and 'b' to a p value < Y? This should be stated. It is also not clear what comparisons have been done for these statistical outcomes - this should also be stated so the reader can understand what is significant statistically or not.

Referee #1:

This is a revised version of a manuscript submitted by Ali et al. via the Review Commons platform. The authors addressed many of my concerns regarding the original manuscript. However, a few minor concerns remain, and a few additional concerns arose when reading the newly added data.

Ans: We thank this reviewer for critically reading this manuscript and valuable comments that help us to improve the manuscript. We have addressed the comments point-by-point as follows.

1. Line 115 - in reference to my comment from the previous round, I meant that the word "novel" to describe a domain published 10 years ago may be inappropriate.

Ans: the term “novel” is removed.

2. (A) Line 142 - I am a bit confused by the citation of Fig. S2 here. Panel A shows results for homologs containing Ntox15, not the N-terminus of Tde1, so I assume panel B is the one that should be cited. Panel B shows two types of operons containing a glycine zipper, but it is unclear to what end the same operon from three agrobacterium strains is shown. Also, not all colors are explained in the legend, making this panel challenging to understand.
(B) Note that the blue rectangle in the middle Tde1 gene in panel B lacks a black frame, and the figure caption for panel A mentions that the numbers are shown on the left, yet they are shown on the right side of the figure.
(C) "FIX-1" domains should be renamed "FIX" of "FIX-like" to match the annotation on NCBI.
(D) Overall, the figure will benefit from text and shape alignment, and from keeping the fonts similar.
(E) Why is Fig. S2 cited before Fig. S1C-F? Perhaps the order of supplementary figures should be reconsidered.

Ans: The authors highly appreciate this reviewer for carefully reading this manuscript in great details. We apologize for the confusion. We have now revised for clarity as follows.

(A) We have reorganized these Supplementary Figures, now referred to Expanded View Figures as EV1-3 based on the order mentioned in the text.
(B) The operons shown are representative operons for Tde1 orthologues and Tape Measure Proteins (TMPs). Thus, we have included the word “representative” in the legend for clarity. We have also corrected the figure captions (see Figure EV2).
(C) We have amended it to FIX-like to be consistent with NCBI annotation as mentioned.
(D) We tried our best to ensure the alignment of text and the shapes and proper font size in the figures.
(E) The figures are reorganized based on the order mentioned in the text.

3. Fig. S1B - please explain what is the "loading control" in the figure caption.

Ans: Thank you for pointing this out. The loading control here is a non-specific band from the western blot of anti-strep and we have now indicated it in the figure legend of Figure EV1.

4. Line 169-170 - there is a noticeable difference of ~2-fold in the number of viable cells (CFU/ml) between the strains in Fig. 2A (with a very small SD). This contradicts the result in the single plate image provided in Fig. S1E. Can the authors explain the discrepancy between the results and why they describe one over the other in the text?

Ans: These two experiments were results of different strains with slightly different experimental setup. For the one in Figure 2A, they are Tde1 variants expressed in *E. col* DH10B while Figure S1E (now EV3C), they are Tde1 variants expressed in *E. coli* BW25113 WT or *lacY* mutant expressing pYTA-*lacZ*. Thus, the difference in the CFU/ml might be caused by differences in strain background and growth conditions.

5. Fig. 2B - (A) Isn't the LacZ plasmid present in all strains, or at least also in the lacY mutant as described in the text? If so, please extend the line at the bottom of the graph to clarify this.
(B) Is "LacY" the lacY mutant? If so, please clarify this in the figure (X-axis) or in the caption.

Ans: Thank you for pointing out. The figure is amended with clear description in the figure legend to avoid confusion now. The plasmid pYTA-*lacZ* was introduced to only the *lacY* mutant cells. The BW23113 was not transformed with pYTA-*lacZ*.

6. Fig. 2C - This is not exactly my area of expertise, but wouldn't one expect that two dyes that bind DNA (Hoechst and PI) will at least somewhat overlap? I find it curious that the Hoechst signals mostly show nucleoid structures (indicating that the cells are probably stressed) while the PI signals are uniformly distributed throughout the cell. Can the authors please comment on this?

Ans: Because PI stains not only DNA but also RNA while Hoechst is a specific DNA dye. Thus, we think it is reasonable to observe more localized Hoechst stains while the PI signals are detected throughout the cell, which is consistent with several literatures (Kiani *et al*, 2021) (Krasauskas *et al*, 2020) (Dar *et al*, 2022; Molina *et al*, 2008).

Dar Y, Jana B, Bosis E, Salomon D (2022) A binary effector module secreted by a type VI secretion system. *EMBO Rep* 23: e53981

Kiani D, Santus W, Kiernan a K, Behnsena J (2021) Proteus mirabilis Employs a Contact-Dependent Killing System against Competing Enterobacteriaceae. *mSphere* 6 e00321-00321

Krasauskas R, Skerniskyte J, Martinkus J, Armalyte J, Suziedeliene E (2020) Capsule Protects Acinetobacter baumannii From Inter-Bacterial Competition Mediated by CdiA Toxin. *Front Microbiol* 11: 1493

Molina F, Sánchez-Romero MA, Jiménez-Sánchez A (2008) Dynamic organization of replication forks into factories in Escherichia coli. *Process Biochemistry* 43: 1171-1177

Line 187 - should "bacteriolytic" be changed to "bactericidal"?

Ans: Thank you for the suggestion, we have amended it accordingly.

8. Fig. 3A - in reference to my comment from the previous round, I am not persuaded by the authors' reply. In co-IP experiments, controls must be performed as part of the same experiment. A control sample cannot come from a separate experiment. At least show for N-Tde1 that its interaction with Tap-1 is specific.

Ans: We agree with the reviewer that the controls should be performed in the same experiment. Indeed, our results that C-Tde1 does not bind Tap1 served as a good control for Tap1-N-Tde1 specific interaction. In addition, our data show that some non-specific protein bands from cellular fractions recognized by anti-HA or anti-Tap1 were not co-immunoprecipitated by N-Tde1-HA (Fig. A3 and see full western blot data below). Although one can choose an available antibody for an irrelevant protein to show N-Tde1 does not bind to another protein, we don't think adding this piece of data can add more information on this point.

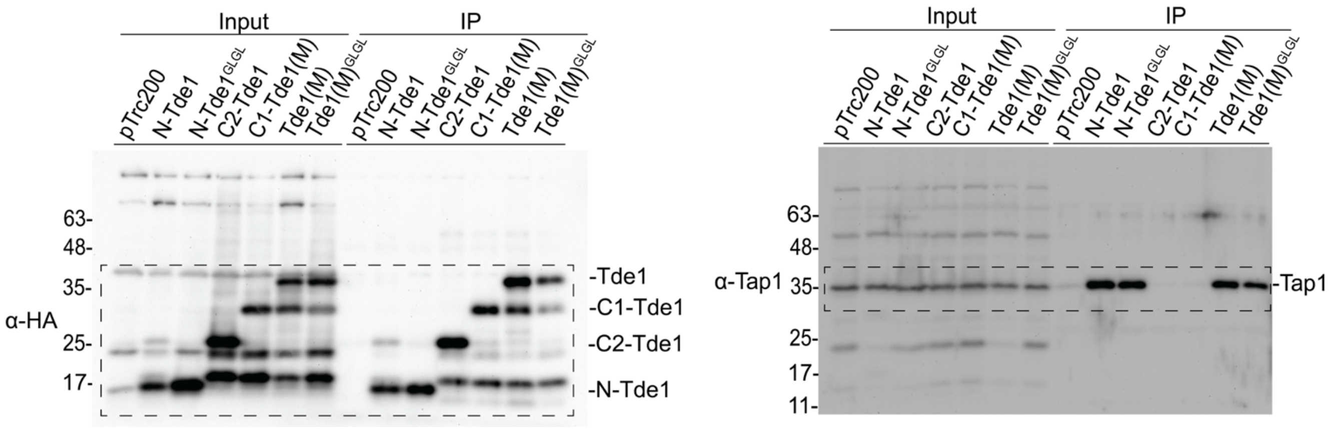

9. Fig. 4 - the legend on the right side of the graph is redundant with the X-axis titles.

Ans: We have amended that by removing the legend on the right side

10. Line 263 and 264 - should "Tdei1" be "Tdi1"?

Ans: Amended.

11. Fig. 5C - why wasn't secretion from the tssK mutant tested for the single glycine mutants (bottom panel) to show that their secretion is dependent on T6SS?

Ans: We have done the secretion of Tde1 single glycine zipper variants in *tssK* mutant to confirm the secretion of the Tde1 single glycine zipper variants are T6SS-dependent. As we already showed T6SS-dependent secretion for WT and double glycine zipper variants in Figure 5C, we only did the secretion of single glycine zipper variants in *tssK* mutant once and so prefer not to include this data in the manuscript.

12. Fig. S5B - Should the bottom rectangles on the gels be marked as Hcp?

Ans: Amended.

Line 320 - if Fig. S2 (I assume this refers to panel A) is cited only here for the first time, perhaps it should not be part of Fig. S2?

Ans: Amended.

Referee #2:

I thank the authors for the work they have done to revise the manuscript. I think that it is significantly improved and there is more support for the conclusions that they draw from their data. I am happy with all of the changes they made to the manuscript in response to my comments in my last review and believe they have adequately addressed all but one of my comments. The outstanding issue I have is how they explain the outcomes of their statistical analyses in their figures. The authors say that "We also rephrased the description as " Different letters indicate statistically different groups of strains" in figure legend for clarity (Fig. 1B; 2C, 2D; 5A, 5B, 5E; S1D)". This is fine but they do not say what the different letters refer to other than the p value being <0.01. Is 'a' referring to a p value <X and 'b' to a p value < Y? This should be stated. It is also not clear what comparisons have been done for these statistical outcomes - this should also be stated so the reader can understand what is significant statistically or not.

Ans: We thank this reviewer for positive considerations of this work and valuable comments that help us to improve the manuscript. Regarding to the statistical analysis, we performed mean separation using Tukey's multiple comparison after obtaining a significant ANOVA. Since Tukey's multiple comparison tests are adjusted for multiple comparisons, the p-value represents the significance of separating different groups, instead of pairwise comparison.

1st Revision - Editorial Decision 8th Mar 2023

Dear Dr. Lai,

Thank you for the submission of your further revised manuscript to our editorial offices. I have now received the report from the referee that I have asked to look into this again, you will find below. As you will see, the referee now fully supports the publication of your study in EMBO reports. I think the controls for the co-IP are acceptable.

Before I can proceed with formal acceptance, I have these further editorial requests I ask you to address in a final revised manuscript:

- I do not like very much the word 'governs' in the title. How about:
A glycine zipper motif is required for the translocation of a T6SS toxic effector into target cells.

or

A glycine zipper motif allows translocation of a T6SS toxic effector into target cells.

- Please reduce the number of keywords to 5.

- Regarding data quantification and statistics, please make sure that the number "n" for how many independent experiments were performed, their nature (biological versus technical replicates), the bars and error bars (e.g. SEM, SD) and the test used to calculate p-values is indicated in the respective figure legends (main, EV and Appendix figures). Please also check that all the p-values are explained in the legend, and that these fit to those shown in the figure. Please provide statistical testing where applicable. Please avoid the phrase 'independent experiment', but clearly state if these were biological or technical replicates. Please also indicate (e.g. with n.s.) if testing was performed, but the differences are not significant.

In case n=2, please show the data as separate datapoints without error bars and statistics.

See also:
http://www.embopress.org/page/journal/14693178/authorguide#statisticalanalysis

If n<5, please show single datapoints for diagrams.

- Please add a formal 'Data Availability Section' (DAS) after the Methods section. This is now mandatory, like the 'Disclosure and Competing Interests Statement'. If no primary datasets have been deposited, please state this in this section (e.g. 'No large primary datasets have been generated and deposited').

- Please make sure that all figure panels are called out separately and sequentially (main, EV and Appendix figures). Presently, there seems to be no separate callout for the panels 4A&B, and EV5A-D. Please check.

- Thanks for providing the source data. Please provide upon re-submission also the filled in source data checklist (attached again). Please make sure that all the requested source data is provided during final revision. Please upload all source data for one figure as one pdf per figure or (if there is more than one file) ZIPed together as one folder. Ideally, there is then one ZIPed folder for each figure. Moreover, please name all the SD files as such (not dataset).

- In Figure 2C PI - pTrc200, it seems the panel/cell shown is empty of any image signal. Source data is provided, but it is not clear which number corresponds to which panel/cell. Each SD has an image in each cell - so this does not explain why 1 cell would be empty. Please clarify or replace the panel with one that contains (background) signal.

- Please provide the information on primers and plasmids as an Appendix file (not as reagents table). This needs to be a single pdf file labeled Appendix. The Appendix should have page numbers and needs to include a table of content on the first page (with page numbers) and legends for all content. Please follow the nomenclature Appendix Table Sx etc. throughout the text, and also label the tables according to this nomenclature. The word file provided is basically fine, but we need this as pdf and uploaded as Appendix. Please also add a title ('Appendix for ...').

- Finally, please find attached a word file of the manuscript text (provided by our publisher) with changes we ask you to include in your final manuscript text and comments. Please use the attached file as basis for further revisions and provide your final manuscript file with track changes, in order that we can see any modifications done.

In addition, I would need from you:
- a short, two-sentence summary of the manuscript (not more than 35 words).
- two to four short bullet points highlighting the key findings of your study (two lines each).

I look forward to seeing the final revised version of your manuscript when it is ready. Please let me know if you have questions regarding the revision.

Please use this link to submit your revision: https://embor.msubmit.net/cgi-bin/main.plex

Best,

Achim Breiling
Senior Editor
EMBO Reports

Referee #1:

The authors have done the necessary modifications to the manuscript. This is an interesting finding of importance to the field.

I leave it to the editor to decide whether the reliance on non-specific bands as co-IP controls is an acceptable practice.

The authors have addressed all minor editorial requests.

2nd Revision - Editorial Decision 22nd Mar 2023

Prof. Erh-Min Lai
Academia Sinica
Institute of Plant and Microbial Biology
128, Sec. 2, Academia Road, Academia Sinica
Taipei 115201
Taiwan

Dear Prof. Lai,

I am very pleased to accept your manuscript for publication in the next available issue of EMBO reports. Thank you for your contribution to our journal.

At the end of this email I include important information about how to proceed. Please ensure that you take the time to read the information and complete and return the necessary forms to allow us to publish your manuscript as quickly as possible.

As part of the EMBO publication's Transparent Editorial Process, EMBO reports publishes online a Review Process File to accompany accepted manuscripts. As you are aware, this File will be published in conjunction with your paper and will include the referee reports, your point-by-point response and all pertinent correspondence relating to the manuscript.

If you do NOT want this File to be published, please inform the editorial office within 2 days, if you have not done so already, otherwise the File will be published by default [contact: emboreports@embo.org]. If you do opt out, the Review Process File link will point to the following statement: "No Review Process File is available with this article, as the authors have chosen not to make the review process public in this case." Please note that the author checklist will still be published even if you opt out of the transparent process.

Thank you again for your contribution to EMBO reports and congratulations on a successful publication. Please consider us again in the future for your most exciting work.

Yours sincerely,

Achim Breiling
Editor
EMBO Reports

****

THINGS TO DO NOW:

Once your article has been received by Wiley for production, the corresponding author will receive an email from Wiley's Author Services system which will ask them to log in and will present them with the appropriate license for completion.

You will receive proofs by e-mail approximately 2-3 weeks after all relevant files have been sent to our Production Office; you should return your corrections within 2 days of receiving the proofs.

Please inform us if there is likely to be any difficulty in reaching you at the above address at that time. Failure to meet our deadlines may result in a delay of publication, or publication without your corrections.

All further communications concerning your paper should quote reference number EMBOR-2023-56849V3 and be addressed to emboreports@wiley.com.

Should you be planning a Press Release on your article, please get in contact with emboreports@wiley.com as early as possible, in order to coordinate publication and release dates.

EMBO Press Author Checklist

Corresponding Author Name: Erh-Min Lai	
Journal Submitted to: EMBO Reports	
Manuscript Number: EMBOR-2023-56849V1-RC-2022-01588	

USEFUL LINKS FOR COMPLETING THIS FORM
The EMBO Journal - Author Guidelines
EMBO Reports - Author Guidelines
Molecular Systems Biology - Author Guidelines
EMBO Molecular Medicine - Author Guidelines

Reporting Checklist for Life Science Articles (updated January 2022)
This checklist is adapted from Materials Design Analysis Reporting (MDAR) Checklist for Authors. MDAR establishes a minimum set of requirements in transparent reporting in the life sciences (see Statement of Task: 10.31222/osf.io/9sm4x). Please follow the journal's guidelines in preparing your manuscript.
Please note that a copy of this checklist will be published alongside your article.

Abridged guidelines for figures
1. Data
The data shown in figures should satisfy the following conditions:
- the data were obtained and processed according to the field's best practice and are presented to reflect the results of the experiments in an accurate and unbiased manner.
- ideally, figure panels should include only measurements that are directly comparable to each other and obtained with the same assay.
- plots include clearly labeled error bars for independent experiments and sample sizes. Unless justified, error bars should not be shown for technical replicates.
- if n<5, the individual data points from each experiment should be plotted. Any statistical test employed should be justified.
- Source Data should be included to report the data underlying figures according to the guidelines set out in the authorship guidelines on Data Presentation.

2. Captions
Each figure caption should contain the following information, for each panel where they are relevant:
- a specification of the experimental system investigated (eg cell line, species name).
- the assay(s) and method(s) used to carry out the reported observations and measurements.
- an explicit mention of the biological and chemical entity(ies) that are being measured.
- an explicit mention of the biological and chemical entity(ies) that are altered/varied/perturbed in a controlled manner.
- the exact sample size (n) for each experimental group/condition, given as a number, not a range;
- a description of the sample collection allowing the reader to understand whether the samples represent technical or biological replicates (including how many animals, litters, cultures, etc.).
- a statement of how many times the experiment shown was independently replicated in the laboratory.
- definitions of statistical methods and measures:
  - common tests, such as t-test (please specify whether paired vs. unpaired), simple χ2 tests, Wilcoxon and Mann-Whitney tests, can be unambiguously identified by name only, but more complex techniques should be described in the methods section;
  - are tests one-sided or two-sided?
  - are there adjustments for multiple comparisons?
  - exact statistical test results, e.g., P values = x but not P values < x;
  - definition of 'center values' as median or average;
  - definition of error bars as s.d. or s.e.m.

Please complete ALL of the questions below.
Select "Not Applicable" only when the requested information is not relevant for your study.

Materials

Newly Created Materials	Information included in the manuscript?	In which section is the information available? (Reagents and Tools Table, Materials and Methods, Figures, Data Availability Section)
New materials and reagents need to be available; do any restrictions apply?	Not Applicable	Appendix Tables, Materials and Methods

Antibodies	Information included in the manuscript?	In which section is the information available? (Reagents and Tools Table, Materials and Methods, Figures, Data Availability Section)
For **antibodies** provide the following information:<br>- Commercial antibodies: RRID (if possible) or supplier name, catalogue number and or/clone number<br>- Non-commercial: RRID or citation	Yes	Materials and Methods

DNA and RNA sequences	Information included in the manuscript?	In which section is the information available? (Reagents and Tools Table, Materials and Methods, Figures, Data Availability Section)
**Short novel DNA or RNA including primers, probes**: provide the sequences.	Yes	Appendix Tables

Cell materials	Information included in the manuscript?	In which section is the information available? (Reagents and Tools Table, Materials and Methods, Figures, Data Availability Section)
**Cell lines:** Provide species information, strain. Provide accession number in repository **OR** supplier name, catalog number, clone number, and/**OR** RRID.	Not Applicable	
**Primary cultures:** Provide species, strain, sex of origin, genetic modification status.	Not Applicable	
Report if the cell lines were recently **authenticated** (e.g., by STR profiling) and tested for mycoplasma contamination.	Not Applicable	

Experimental animals	Information included in the manuscript?	In which section is the information available? (Reagents and Tools Table, Materials and Methods, Figures, Data Availability Section)
**Laboratory animals or Model organisms:** Provide species, strain, sex, age, genetic modification status. Provide accession number in repository **OR** supplier name, catalog number, clone number, **OR** RRID.	Not Applicable	
**Animal observed in or captured from the field:** Provide species, sex, and age where possible.	Not Applicable	
Please detail **housing and husbandry conditions**.	Not Applicable	

Plants and microbes	Information included in the manuscript?	In which section is the information available? (Reagents and Tools Table, Materials and Methods, Figures, Data Availability Section)
**Plants:** provide species and strain, ecotype and cultivar where relevant, unique accession number if available, and source (including location for collected wild specimens).	Not Applicable	
**Microbes:** provide species and strain, unique accession number if available, and source.	Yes	Materials and Methods, Figures

Human research participants	Information included in the manuscript?	In which section is the information available? (Reagents and Tools Table, Materials and Methods, Figures, Data Availability Section)
If collected and within the bounds of privacy constraints report on age, sex and gender or ethnicity for all study participants.	Not Applicable	

Core facilities	Information included in the manuscript?	In which section is the information available? (Reagents and Tools Table, Materials and Methods, Figures, Data Availability Section)
If your work benefited from core facilities, was their service mentioned in the acknowledgments section?	Yes	Acknowledgements

Design

Study protocol	Information included in the manuscript?	In which section is the information available? (Reagents and Tools Table, Materials and Methods, Figures, Data Availability Section)
If study protocol has been **pre-registered, provide DOI in the manuscript**. For clinical trials, provide the trial registration number **OR** cite DOI.	Not Applicable	
Report the **clinical trial registration number** (at ClinicalTrials.gov or equivalent), where applicable.	Not Applicable	

Laboratory protocol	Information included in the manuscript?	In which section is the information available? (Reagents and Tools Table, Materials and Methods, Figures, Data Availability Section)
Provide DOI OR other citation details if **external detailed step-by-step protocols** are available.	Not Applicable	

Experimental study design and statistics	Information included in the manuscript?	In which section is the information available? (Reagents and Tools Table, Materials and Methods, Figures, Data Availability Section)
Include a statement about **sample size** estimate even if no statistical methods were used.	Not Applicable	
Were any steps taken to minimize the effects of subjective bias when allocating animals/samples to treatment (e.g. **randomization procedure**)? If yes, have they been described?	Not Applicable	
Include a statement about **blinding** even if no blinding was done.	Not Applicable	
Describe **inclusion/exclusion criteria** if samples or animals were excluded from the analysis. Were the criteria pre-established?<br><br>If sample or data points were omitted from analysis, report if this was due to attrition or intentional exclusion and provide justification.	Not Applicable	
For every figure, are **statistical tests** justified as appropriate? Do the data meet the assumptions of the tests (e.g., normal distribution)? Describe any methods used to assess it. Is there an estimate of variation within each group of data? Is the variance similar between the groups that are being statistically compared?	Yes	Normality test checked before ANOVA

Sample definition and in-laboratory replication	Information included in the manuscript?	In which section is the information available? (Reagents and Tools Table, Materials and Methods, Figures, Data Availability Section)
In the figure legends: state number of times the experiment was **replicated** in laboratory.	Yes	Figure legends
In the figure legends: define whether data describe **technical or biological replicates**.	Yes	Figure legends

Ethics

Ethics	Information included in the manuscript?	In which section is the information available? (Reagents and Tools Table, Materials and Methods, Figures, Data Availability Section)
Studies involving **human participants**: State details of **authority granting ethics approval** (IRB or equivalent committee(s), provide reference number for approval.	Not Applicable	
Studies involving **human participants**: Include a statement confirming that **informed consent** was obtained from all subjects and that the experiments conformed to the principles set out in the WMA Declaration of Helsinki and the Department of Health and Human Services Belmont Report.	Not Applicable	
Studies involving **human participants:** For publication of **patient photos**, include a statement confirming that consent to publish was obtained.	Not Applicable	
Studies involving experimental **animals**: State details of **authority granting ethics approva**l (IRB or equivalent committee(s), provide reference number for approval. Include a statement of compliance with ethical regulations.	Not Applicable	
Studies involving **specimen and field samples:** State if relevant **permits** obtained, provide details of authority approving study; if none were required, explain why.	Not Applicable	

Dual Use Research of Concern (DURC)	Information included in the manuscript?	In which section is the information available? (Reagents and Tools Table, Materials and Methods, Figures, Data Availability Section)
Could your study fall under dual use research restrictions? Please check biosecurity documents and list of **select agents and toxins** (CDC): https://www.selectagents.gov/sat/list.htm	Not Applicable	
If you used a select agent, is the security level of the lab appropriate and reported in the manuscript?	Not Applicable	
If a study is subject to dual use research of concern regulations, is the name of the **authority granting approval and reference number** for the regulatory approval provided in the manuscript?	Not Applicable	

Reporting

The MDAR framework recommends adoption of discipline-specific guidelines, established and endorsed through community initiatives. Journals have their own policy about requiring specific guidelines and recommendations to complement MDAR.

Adherence to community standards	Information included in the manuscript?	In which section is the information available? (Reagents and Tools Table, Materials and Methods, Figures, Data Availability Section)
State if relevant guidelines or checklists (e.g., **ICMJE, MIBBI, ARRIVE, PRISMA**) have been followed or provided.	Not Applicable	
For **tumor marker prognostic studies,** we recommend that you follow the **REMARK** reporting guidelines (see link list at top right). See author guidelines, under 'Reporting Guidelines'. Please confirm you have followed these guidelines.	Not Applicable	
For **phase II and III randomized controlled trials**, please refer to the **CONSORT** flow diagram (see link list at top right) and submit the CONSORT checklist (see link list at top right) with your submission. See author guidelines, under 'Reporting Guidelines'. Please confirm you have submitted this list.	Not Applicable	

Data Availability

Data availability	Information included in the manuscript?	In which section is the information available? (Reagents and Tools Table, Materials and Methods, Figures, Data Availability Section)
Have **primary datasets** been deposited according to the journal's guidelines (see 'Data Deposition' section) and the respective accession numbers provided in the Data Availability Section?	Yes	Data Availability Section
Were **human clinical and genomic datasets** deposited in a public access-controlled repository in accordance to ethical obligations to the patients and to the applicable consent agreement?	Not Applicable	
Are **computational models** that are central and integral to a study available without restrictions in a machine-readable form? Were the relevant accession numbers or links provided?	Not Applicable	
If publicly available data were reused, provide the respective **data citations in the reference list**.	Not Applicable	
